# C–Cl Oxidative Addition and C–C Reductive Elimination Reactions in the Context of the Rhodium-Promoted Direct Arylation

**DOI:** 10.1021/acs.organomet.1c00643

**Published:** 2022-03-17

**Authors:** Laura A. de las Heras, Miguel A. Esteruelas, Montserrat Oliván, Enrique Oñate

**Affiliations:** Departamento de Química Inorgánica—Instituto de Síntesis Química y Catálisis Homogénea (ISQCH)—Centro de Innovación en Química Avanzada (ORFEO-CINQA), Universidad de Zaragoza—CSIC, 50009 Zaragoza, Spain

## Abstract

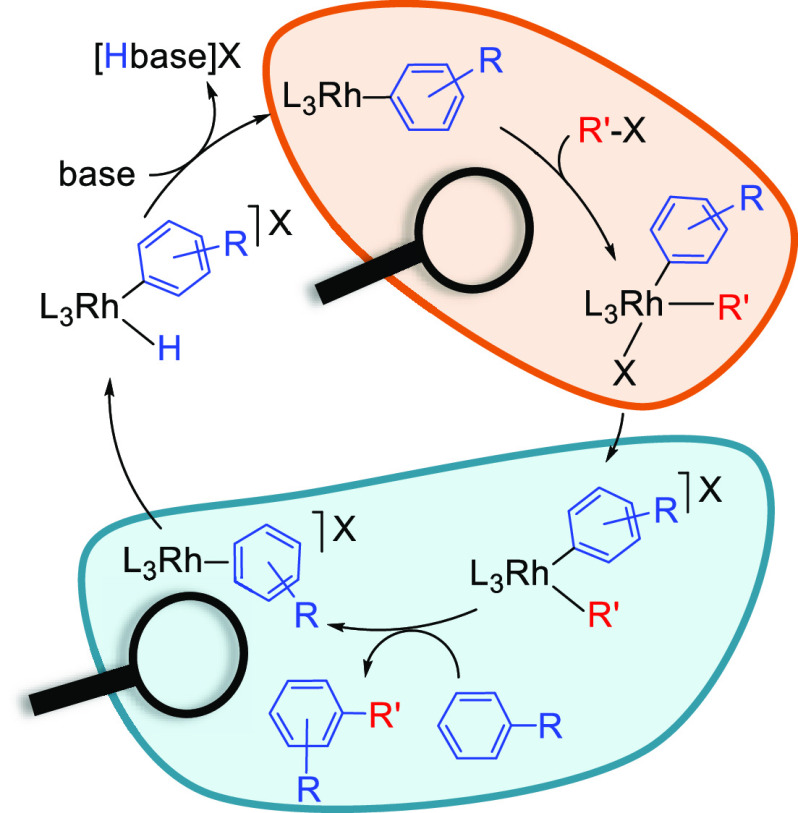

A cycle of stoichiometric elemental reactions defining the direct arylation promoted by a redox-pair Rh(I)–Rh(III) is reported. Starting from the rhodium(I)-aryl complex RhPh{κ^3^-P,O,P-[xant(P^i^Pr_2_)_2_]} (xant(P^i^Pr_2_)_2_ = 9,9-dimethyl-4,5-bis(diisopropylphosphino)xanthene), the reactions include C–Cl oxidative addition of organic chlorides, halide abstraction from the resulting six-coordinate rhodium(III) derivatives, C–C reductive coupling between the initial aryl ligand and the added organic group, oxidative addition of a C–H bond of a new arene, and deprotonation of the generated hydride-rhodium(III)-aryl species to form a new rhodium(I)-aryl derivative. In this context, the kinetics of the oxidative additions of 2-chloropyridine, chlorobenzene, benzyl chloride, and dichloromethane to RhPh{κ^3^-P,O,P-[xant(P^i^Pr_2_)_2_]} and the C–C reductive eliminations of biphenyl and benzylbenzene from [RhPh_2_{κ^3^-P,O,P-[xant(P^i^Pr_2_)_2_]}]BF_4_ and [RhPh(CH_2_Ph){κ^3^-P,O,P-[xant(P^i^Pr_2_)_2_]}]BF_4_, respectively, have been studied. The oxidative additions generally involve the cis addition of the C–Cl bond of the organic chloride to the rhodium(I) complex, being kinetically controlled by the C–Cl bond dissociation energy; the weakest C–Cl bond is faster added. The C–C reductive elimination is kinetically governed by the dissociation energy of the formed bond. The C(sp^3^)–C(sp^2^) coupling to give benzylbenzene is faster than the C(sp^2^)–C(sp^2^) bond formation to afford biphenyl. In spite of that a most demanding orientation requirement is needed for the C(sp^3^)–C(sp^2^) coupling than for the C(sp^2^)–C(sp^2^) bond formation, the energetic effort for the pregeneration of the C(sp^3^)–C(sp^2^) bond is lower. As a result, the weakest C–C bond is formed faster.

## Introduction

**Scheme 1:**
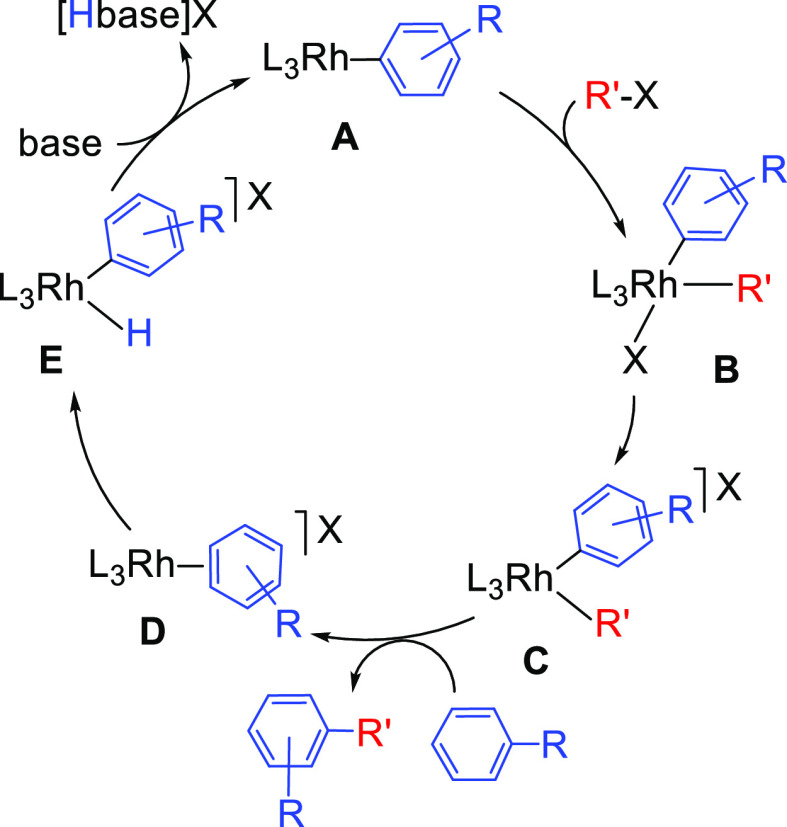
Elemental Steps for the Rhodium-Promoted C–H Arylation

Transition metal-catalyzed C–C cross-coupling reactions are among the industrial technologies of the highest significance.^1^ The direct C–H arylation with organic halides is especially appealing among the reactions of this family because it represents a powerful, valuable, and straightforward procedure for nonactivated C–H bond functionalization.^2^ In this context, without a shadow of a doubt, palladium(0) complexes dominate the scene, being the most used catalysts.^3^ However, examples proving the efficiency of rhodium derivatives have been also reported in recent years,^4^ particularly when alkyl halides are employed.^5^ Three fundamental reactions are the base of the process from the mechanism point of view: the oxidative additions of C–Cl^6^ and C–H^7^ bonds, one of each substrate, to an unsaturated d^*n*^-metal center in low oxidation state and the C–C reductive elimination from a d^*n*–2^-metal intermediate.^8^ For a rhodium catalyst, these reactions can be ordered according to the tentative cycle shown in Scheme 1. Thus, the design of the optimal catalyst requires sequencing the splitting of the σ-bonds and the C–C bond formation, in the metal coordination sphere, for which an adequate difference between the activation energies of such elemental steps is crucial. The success of the cross-coupling demands a deep knowledge of the factors governing such reactions.

Halides are versatile functional groups; organic halides are classified as core building blocks in organic synthesis.^9^ In the catalytic cycle shown in Scheme 1, a square-planar rhodium(I)-aryl complex **A** undergoes oxidative addition of a C–X (X = Cl, Br, and I) bond of an organic halide. The **A**-type complexes are hardly isolable and therefore their number is scarce,^10^ and as a consequence, the study of this first step of the cycle is a challenge, which as far as we know has not been addressed. Nevertheless, in agreement with the catalytic rhodium use in the C–H arylation, the oxidative addition of C–X bonds to other rhodium(I) complexes has attracted notable interest, in particular, the reactions involving C(sp^2^)–Cl^10d,10i,11^ and C(sp^3^)–Cl^12^ additions. Although the C–X bond strength decreases on going down to group 17, the organic chlorides are more interesting substrates than bromides and iodides by their lower cost and wider diversity. Once formed, the six-coordinate rhodium(III) intermediate **B**, bearing two single Rh–C bonds, the subsequent halide dissociation should allow an unsaturated five-coordinate species **C**, which would undergo C–C reductive elimination to generate the product of the catalysis. The latter is certainly the critical step in the cross-coupling process. However, in spite of its relevance, many basic questions about the factors that govern it remain largely unanswered. The importance of the five-coordinate intermediates is well-established for the C–C reductive elimination in platinum(IV) complexes, from both experimental^13^ and theoretical^14^ points of view. For a five-coordinate species, trigonal bipyramids or square pyramids are the usual polyhedrons defined by the donor atoms of the ligands around the metal center. The C–C reductive elimination is stereo-controlled; for the trigonal bipyramid arrangement, the coupling of two equatorial groups is favored with regard to the axial-equatorial coupling, while for the square pyramid disposition, the coupling of basal and axial ligands is favored with regard to the coupling of two basal groups. Thus, the electronic nature of the ligands along with their rigidity or flexibility, which ascertain the donor atom disposition around the metal center and constrain the interconversion between the polyhedrons, is a crucial factor for the C–C coupling, in particular, when pincer groups are used to stabilize the system.^15^ Because such five-coordinate species are the key for understanding the C–C coupling, their isolation and study should be an imperative target, but unfortunately, they display scarce stability and have been rarely isolated.^11a,16^ In the presence of an arene, the C–C reductive elimination should afford a rhodium(I)-arene derivative **D**, which would evolve to the hydride-rhodium(III)-aryl intermediate **E** by oxidative addition of one C(sp^2^)–H bond of the arene. Thus, the deprotonation of the metal center of **E** could regenerate the square-planar rhodium(I)-aryl complex **A**. The Brønsted–Lowry acid character of cationic transition metal-hydride compounds is well-known.^17^

Weller’s group has proved that POP diphosphines are hemilabile ligands.^18^ In 2010, we prepared 9,9-dimethyl-4,5-bis(diisopropylphosphino)xanthene (xant(P^i^Pr_2_)_2_), among other ether diphosphines.^19^ This ligand displays more coordinating flexibility than the classical POP diphosphines. Thus, species with the ligand bonded in the modes κ^2^-P,P-*cis* and κ^2^-P,P-*trans*, which prove the hemilabile character of the oxygen atom, are also known in this case.^20^ However, the pincer κ^3^-P,O,P-*mer* coordination is the most usual,^10g−10i,12g,21^ although complexes bearing the diphosphine κ^3^-P,O,P-*fac*-coordinated have been additionally reported.^20e,22^ Accordingly, diphosphine xant(P^i^Pr_2_)_2_ allows structural changes in its complexes, to adapt the metal coordination sphere to the needs of the reactions. As a result, a number of metal derivatives stabilized by this ligand have proven to be active catalysts for a range of interesting organic transformations,^10h,20a,20d,21b,21f−21h,23^ including cross-coupling reactions that involve elemental steps of σ-bond activation in both substrates such as the borylation^10g,24^ and silylation^25^ of arenes. As a part of the chemistry of the Rh-xant(P^i^Pr_2_)_2_ moiety, we have previously reported that the square-planar rhodium(I)-hydride complex RhH{κ^3^-P,O,P-[xant(P^i^Pr_2_)_2_]} activates C–H and C–Cl bonds of arenes to afford the corresponding rhodium(III) species RhH_2_(aryl){κ^3^-P,O,P-[xant(P^i^Pr_2_)_2_]} and RhH(aryl)Cl{κ^3^-P,O,P-[xant(P^i^Pr_2_)_2_]}, which eliminate H_2_ and HCl, respectively, to form a wide variety of square-planar derivatives Rh(aryl){κ^3^-P,O,P-[xant(P^i^Pr_2_)_2_]}.^10g,10i^ The coordinating flexibility of xant(P^i^Pr_2_)_2_, the success of some of its metal derivatives as catalysts for cross-coupling reactions formed by elemental steps involving σ-bond activation-coupling, and the easy accessibility to **A**-type complexes prompted us to study two key steps of the cycle shown in Scheme 1, the oxidative addition of C(sp^2^)–Cl and C(sp^3^)–Cl bonds to **A** and the C–C reductive elimination from **C** in the presence of an arene, for four substrates: 2-chloropyridine, chlorobenzene, benzyl chloride, and dichloromethane.

This paper shows a comparative study about the oxidative addition of the previously mentioned substrates to the aryl complex RhPh{κ^3^-P,O,P-[xant(P^i^Pr_2_)_2_]}, the transformation of the resulting six-coordinate derivatives into five-coordinate species, and the C–C reductive elimination from the unsaturated compounds, in the presence of fluorobenzene, also in a comparative manner.

## Results and Discussion

### Oxidative Addition Reactions

**Scheme 2:**
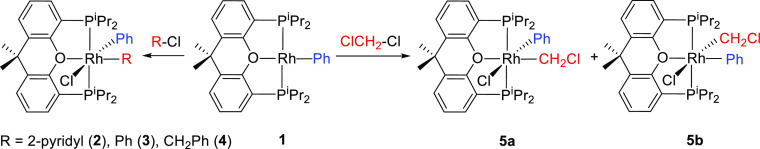
Oxidative Addition Reactions

The behavior of the square-planar rhodium(I) complex RhPh{κ^3^-P,O,P-[xant(P^i^Pr_2_)_2_]} (**1**) toward 2-chloropyridine, chlorobenzene, benzyl chloride, and dichloromethane is summarized in Scheme 2.

**Figure 1:**
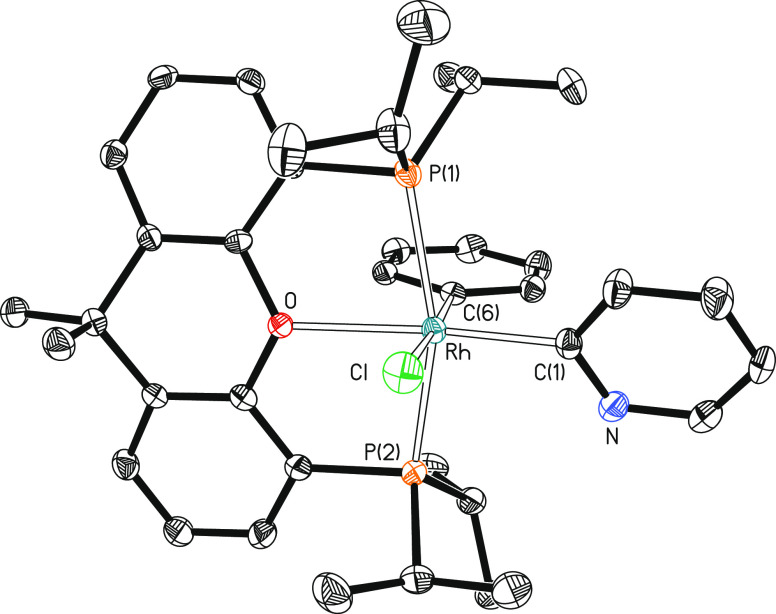
Molecular diagram of complex **2** (ellipsoids shown at 50% probability). All hydrogen atoms are omitted for clarity. Selected bond distances (Å) and angles (°): Rh–P(1) = 2.3541(7), Rh–P(2) = 2.3071(7), Rh–Cl = 2.4520(7), Rh–C(1) = 1.995(2), Rh–C(6) = 2.055(2), Rh–O = 2.2874(16); P(1)-Rh-P(2) = 161.39(2), Cl–Rh–C(1) = 88.01(7), Cl–Rh–C(6) = 177.12(7), O–Rh–C(1) = 173.56(8), and C(1)-Rh-C(6) = 93.67(10).

The reactions were not influenced by light neither by the presence of 5 mol % of hydroquinone. According to such findings, radicals do not appear to play any role during the processes. Stirring of **1** in 2-chloropyridine, at 50 °C, for 48 h gives rise to one rhodium(III) stereoisomer of those possible for the formula Rh(Ph)(2-pyridyl)Cl{κ^3^-P,O,P-[xant(P^i^Pr_2_)_2_]} (**2**). This species is generated as a result of the oxidative addition of the C(sp^2^)–Cl bond of the solvent to the metal center of **1**. Complex **2** was isolated as a yellow solid in 56% and characterized by X-ray diffraction analysis. In accordance with the stereochemistry depicted in Scheme 2 for **2**, the structure (Figure 1) reveals that the isolated isomer bears a pyridyl-*trans*-oxygen disposition (O–Rh–C(1) = 173.56(9)°). Thus, the polyhedron around the rhodium atom can be idealized as the expected octahedron with the ether-diphosphine *mer*-coordinated and the chloride ligand disposed trans to the phenyl group. The NMR spectra (Figures S31–S33) in benzene-*d*_6_ are consistent with this ligand disposition. In agreement with the equivalence of the P^i^Pr_2_ groups of the pincer, the ^31^P{^1^H} spectrum shows a doublet (^1^*J*_P–Rh_ = 119 Hz) at 27.7 ppm. In the ^13^C{^1^H} spectrum, the resonances corresponding to the metalated carbon atoms are observed at 143.7 (Ph) and 173.9 (pyridyl) ppm as doublets of triplets with C–Rh and C–P coupling constants of 34 and 40 Hz and 10 and 6 Hz, respectively.

**Figure 2:**
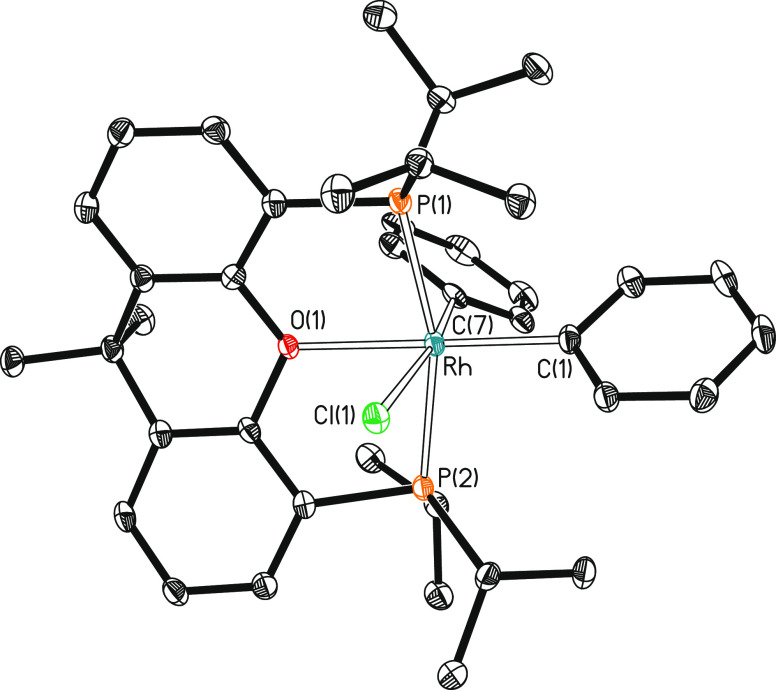
Molecular diagram of complex **3** (ellipsoids shown at 50% probability). All hydrogen atoms are omitted for clarity. Selected bond distances (Å) and angles (°): Rh–P(1) = 2.3164(6), Rh–P(2) = 2.3472(6), Rh–Cl(1) = 2.5058(6), Rh–C(1) = 2.0348(18), Rh–C(7) = 2.0382(19), Rh–O(1) = 2.2448(13); P(1)-Rh-P(2) = 162.736(18), Cl(1)-Rh-C(1) = 97.25(6), Cl(1)-Rh-C(7) = 169.20(5), O(1)-Rh-C(1) = 177.50(6), and C(1)-Rh-C(7) = 93.17(7).

The reaction of **1** with chlorobenzene was also performed in the neat organic halide as a solvent, in this case at 90 °C. Under these conditions, the oxidative addition product RhPh_2_Cl{κ^3^-P,O,P-[xant(P^i^Pr_2_)_2_]} (**3**) was obtained as a yellowish white solid in 76% yield, after 48 h. Its structure (Figure 2) resembles that of **2** with one of the phenyl ligands in the position of the pyridyl group, disposed trans to the oxygen atom of the diphosphine (O–Rh–C(1) = 177.50(7)°). In agreement with the presence of two inequivalent phenyl ligands in the complex, the ^13^C{^1^H} NMR spectrum (Figure S36) in benzene-*d*_6_ displays two doublets (^1^*J*_C–Rh_ = 39 and 33 Hz) of triplets (^2^*J*_C–P_ = 9 and 8 Hz) at 146.4 (trans to Cl) and 152.7 (trans to O) ppm. In accordance with **2**, the ^31^P{^1^H} NMR spectrum (Figure S35) shows a doublet (^1^*J*_P–Rh_ = 114 Hz) at 26.5 ppm, for the equivalent P^i^Pr_2_ groups of the pincer.

**Figure 3:**
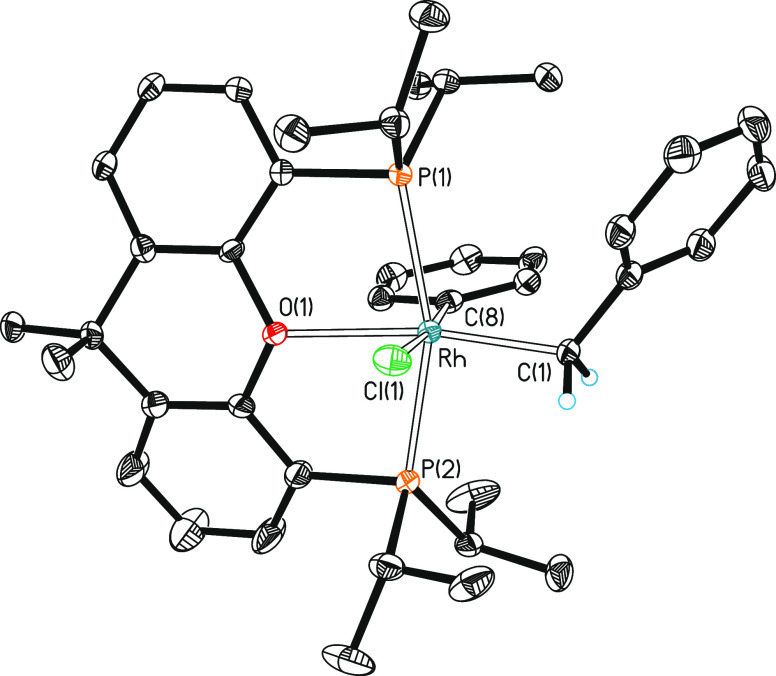
Molecular diagram of complex **4** (ellipsoids shown at 50% probability). All hydrogen atoms (except those of the CH_2_ moiety) are omitted for clarity. Selected bond distances (Å) and angles (°): Rh–P(1) = 2.3578(6), Rh–P(2) = 2.3206(6), Rh–Cl(1) = 2.4682(6), Rh–C(1) = 2.091(2), Rh–C(8) = 2.053(2), Rh–O(1) = 2.3217(15); P(1)-Rh-P(2) = 160.93(2), Cl(1)-Rh-C(1) = 90.71(7), Cl(1)-Rh-C(8) = 174.90(6), O(1)-Rh-C(1) = 169.64(7), and C(1)-Rh-C(8) = 93.00(9).

The C(sp^3^)–Cl oxidative additions to **1** seem to have activation barriers lower than the additions of a C(sp^2^)–Cl bond. In contrast to 2-chloropyridine and chlorobenzene, benzyl chloride instantaneously reacts with **1**, at room temperature, even using stoichiometric amounts of the reagents. The oxidative addition product, the benzyl-aryl complex RhPh(CH_2_Ph)Cl{κ^3^-P,O,P-[xant(P^i^Pr_2_)_2_]} (**4**), was isolated as a white solid in 80% yield and characterized by X-ray diffraction analysis. The structure (Figure 3) is consistent with that of **2**, showing that the generated benzyl ligand is disposed trans to the oxygen atom of the pincer (O–Rh–C(1) = 169.64(7)°). The ^1^H and ^13^C{^1^H} NMR spectra (Figures S37 and S39) in benzene-*d*_6_ are consistent with the presence of the benzyl ligand in the complex. Thus, the ^1^H spectrum shows a doublet (^2^*J*_H–Rh_ = 3.2 Hz) of triplets (^3^*J*_H–P_ = 3.6 Hz) at 5.02 ppm, which fits with other doublet (^1^*J*_C–Rh_ = 29 Hz) of triplets (^2^*J*_C–P_ = 5 Hz) at 16.8 ppm in the ^13^C{^1^H} spectrum, both due to the CH_2_ group. In accordance with **2** and **3**, the ^13^C{^1^H} spectrum also contains a doublet (^1^*J*_C–Rh_ = 33 Hz) of triplets (^2^*J*_C–P_ = 11 Hz) at 141.9 ppm, corresponding to the metalated carbon atom of the phenyl ligand, whereas the ^31^P{^1^H} spectrum (Figure S38) displays a doublet (^1^*J*_P–Rh_ = 118 Hz) at 22.4 ppm for the equivalent P^i^Pr_2_ groups of the diphosphine.

**Scheme 3:**
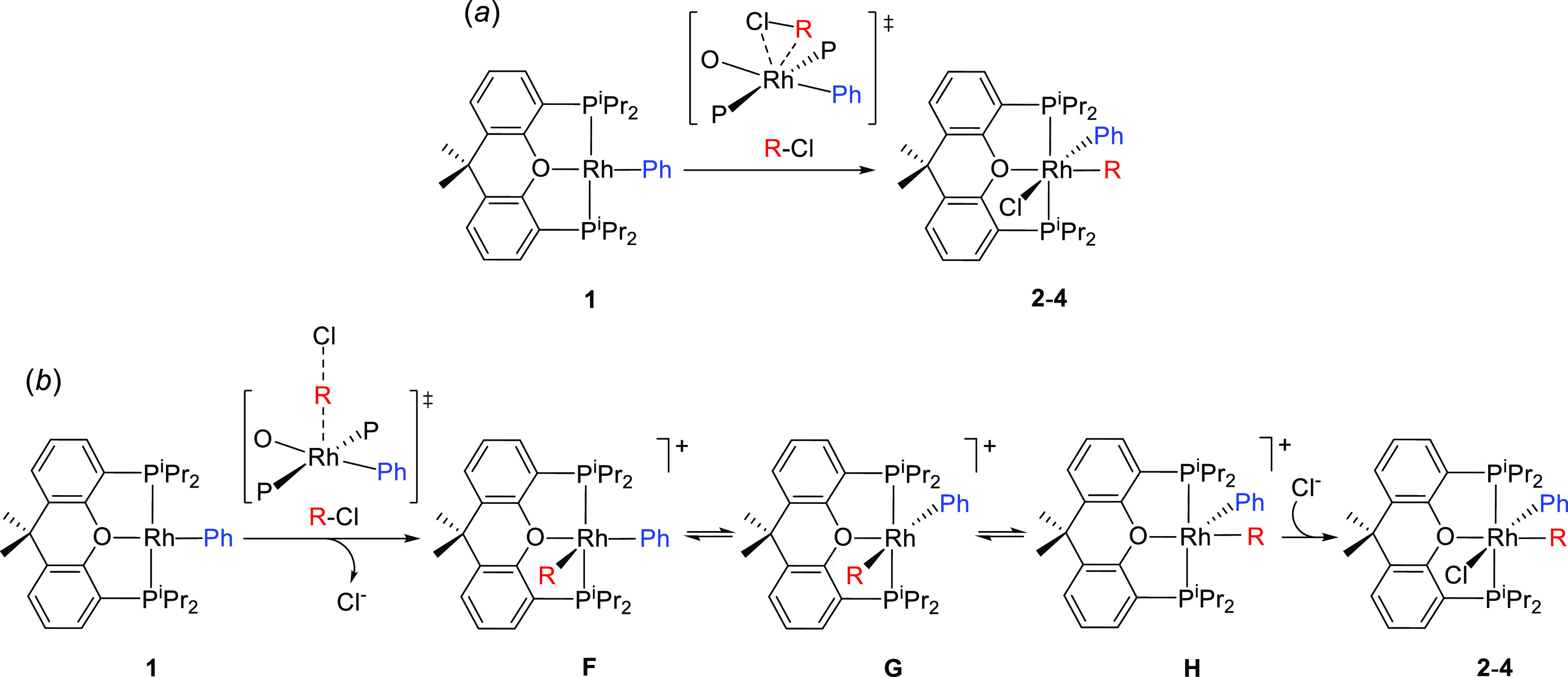
Plausible Mechanisms for the Formation of Complexes **2–4**

Complexes **2–4** are the result of a cis-addition of the C–Cl bond of the organic chlorides to **1**. Keeping the pincer skeleton, this addition could in principle take place in a direct manner or by steps (Scheme 3). The direct form involves a concerted addition along the O–Rh–Ph axis, with the chlorine substituent of the substrate above the oxygen atom of the diphosphine (a). The addition by steps should be initiated by an S_N_2-type rupture and requires a thermodynamic control of the stereochemistry (b); the five-coordinate intermediate resulting from the C–Cl rupture (**F**; R trans to the coordination vacancy) would undergo an isomerization process of a low activation barrier, which could involve a phenyl shift of 90° in the perpendicular plane to the P–Rh–P direction to afford a new five-coordinate square pyramidal intermediate **G**, with the diphosphine oxygen atom trans to the coordination vacancy, followed by an R shift of 90° to locate the added organic fragment trans to the oxygen atom and cis to the coordination vacancy. In this way, the entry of the chloride in the coordination vacancy of **H** could give the obtained compounds.

**Figure 4:**
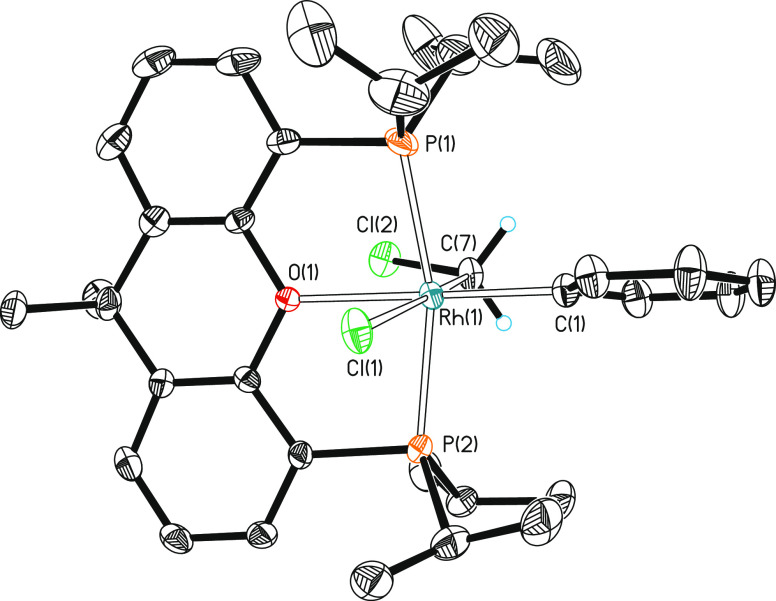
Molecular diagram of complex **5b** (ellipsoids shown at 50% probability). All hydrogen atoms (except those of the CH_2_ moiety) are omitted for clarity. Selected bond distances (Å) and angles (°): Rh(1)–P(1) = 2.3191(14), 2.3312(13), Rh(1)–P(2) = 2.3361(13), 2.3412(12), Rh(1)–Cl(1) = 2.4966(13), 2.4893(14), Rh(1)–C(1) = 2.028(5), 2.029(5), Rh(1)–C(7) = 2.042(5), 2.054(5), Rh(1)–O(1) = 2.222(3), 2.241(3); P(1)-Rh(1)-P(2) = 164.74(5), 163.76(5), Cl(1)-Rh(1)-C(1) = 98.25(15), 97.42(16), Cl(1)-Rh(1)-C(7) = 173.04(15), 174.19(16), O(1)-Rh(1)-C(1) = 176.50(17), 176.45(17), C(1)-Rh(1)-C(7) = 88.5(2), 88.4(2).

The oxidative addition of one of the C(sp^3^)–Cl bonds of dichloromethane to **1** shows significant differences with regard to the reaction with benzyl chloride. It must be performed in the halide as a solvent or using a great excess and leads to two different isomers of formula RhPh(CH_2_Cl)Cl{κ^3^-P,O,P-[xant(P^i^Pr_2_)_2_]} (**5a** and **5b**) in a 1.1:1 molar ratio. For one of them, **5b**, crystals suitable for X-ray diffraction analysis were obtained. Its structure (Figure 4) revealed a mutually trans disposition for the added fragments (Cl(1)-Rh(1)-C(7) = 173.04(15) and 174.19(16)°).^26^ The formation of two isomers is strongly supported by the NMR spectra (Figures S40–S42), in dichloromethane-*d*_2_, at room temperature. The ^1^H spectrum shows two CH_2_Cl resonances at 5.76 and 4.79 ppm, which are observed as doublets of triplets with H–Rh and H–P coupling constants of about 3 and 7 Hz, respectively. The resonance at the lower field was assigned to isomer **5a** (CH_2_Cl trans to O) on the base of the stronger *trans*-effect of ether regarding chloride.^27^ The ^13^C{^1^H} spectrum contains two sets of two doublets of triplets; one of them close to 141 ppm (^1^*J*_C–Rh_ ≈ 35 Hz, ^2^*J*_C–P_ = 10 Hz) due to the metalated carbon atom of the phenyl ligand and the other around 40 ppm (^1^*J*_C–Rh_ ≈ 35 Hz, ^2^*J*_C–P_ ≈ 7 Hz) corresponding to the CH_2_Cl group. Doublets at 27.5 (^1^*J*_P–Rh_ = 114 Hz) and 27.3 (^1^*J*_P–Rh_ = 110 Hz) ppm in the ^31^P{^1^H} spectrum are also features of these species. Once the mixture is formed, its composition does not change with the temperature, indicating that the isomerization between **5a** and **5b** is not kinetically accessible.

The previous observations in a qualitative manner point out that the activation barrier for the oxidative addition increases in the sequence benzyl chloride < dichloromethane < 2-chloropyridine < chlorobenzene and that the cis addition of the C–Cl bond is favored with regard to the trans one; thus, only in the dichloromethane case, both types of additions are observed. In addition, it should be noted that the chloride-*trans*-oxygen disposition is elusive. The presence of two π-donor groups on the same metal orbital most probably produces a decrease in the stability of such isomers with regard to those observed, which bear a chloride-*trans*-phenyl disposition. In order to quantitatively confirm the activation barrier sequence and to gain information of the intimate details of the additions, we studied the kinetics of the reactions of 2-chloropyridine, chlorobenzene, and dichloromethane, those occurring at rates that allow the study, by ^31^P{^1^H} NMR spectroscopy.

The oxidative additions of 2-chloropyridine and chlorobenzene to **1** in the neat organic halide as a solvent are pseudo-first-order processes, which fit to the expression shown in eq 1, where [**1**]_0_ is the initial concentration of **1** and [**1**] is the concentration at the time *t*. The values of the observed *k*_1_ in the temperature range studied are gathered in Table 1. The activation parameters obtained from the respective Eyring analysis (Figures S6 and S12) are Δ*H*^⧧^ = 11.6 ± 2.2 kcal mol^–1^, Δ*S*^⧧^ = −41.1 ± 6.5 cal K^–1^ mol^–1^, and Δ*G*_298_^⧧^ = 23.8 ± 4.1 kcal mol^–1^ for 2-chloropyridine and Δ*H*^⧧^ = 13.3 ± 1.6 kcal mol^–1^, Δ*S*^⧧^ = −44.0 ± 4.2 cal K^–1^ mol^–1^, and Δ*G*_298_^⧧^ = 26.4 ± 2.9 kcal mol^–1^ for chlorobenzene. The marked negative values of the activation entropy are consistent with a concerted addition along the O–Rh–Ph axis with the aromatic ring of the organic halide on the phenyl group (a in Scheme 3). Thus, π–π interactions between the aromatic rings could contribute to increase the order in the transition state.
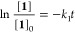
1

**Table 1:** Rate Constants (*k*_1_, s^–1^) for the Formation of Complexes **2** and **3**

complex **2**		complex **3**	
*T* (K)	*k*_1_ (s^–1^)	*T* (K)	*k*_1_ (s^–1^)
323	(9.6 ± 0.6) × 10^–5^	363	(1.8 ± 0.2) × 10^–5^
328	(1.5 ± 0.1) × 10^–4^	373	(3.6 ± 0.2) × 10^–5^
333	(1.9 ± 0.2) × 10^–4^	383	(4.6 ± 0.4) × 10^–5^
338	(2.3 ± 0.2) × 10^–4^	393	(8.7 ± 0.6) × 10^–5^
343	(3.1 ± 0.2) × 10^–4^	398	(1.0 ± 0.1) × 10^–4^

**Figure 5:**
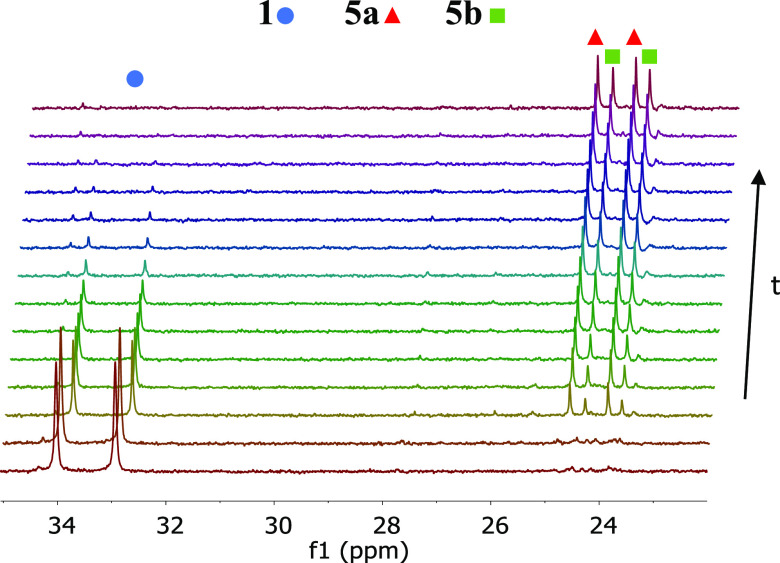
Stacked ^31^P{^1^H} NMR spectra (161.98 MHz, toluene-*d*_8_, 288 K) showing the reaction of **1** with dichloromethane as a function of time.

**Figure 6:**
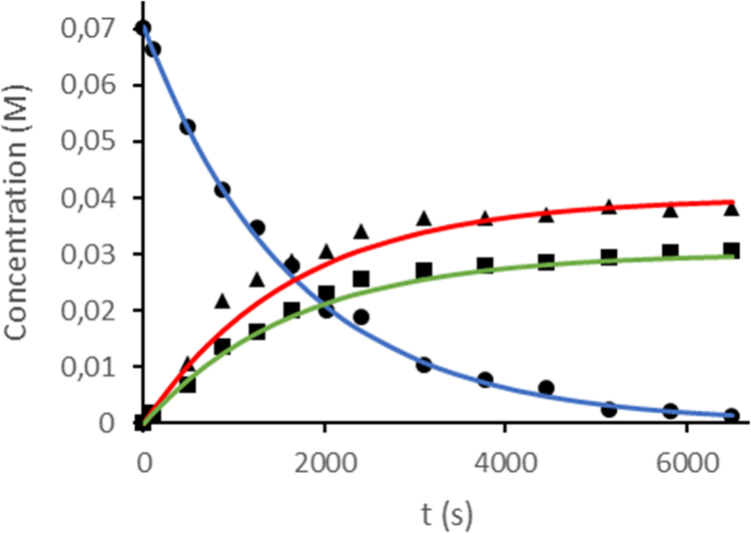
Composition of the mixture as a function of time for the reaction of **1** with dichloromethane at 288 K (**1**, black ●; **5a** black ▲; and **5b** black ■). Fits to eqs 2–4 are given in color.

Figure 5 shows the ^31^P{^1^H} NMR spectra of the addition of dichloromethane to **1**, as a function of time, under pseudo-first-order conditions (20 equiv CH_2_Cl_2_), at 288 K. The dependence of the concentrations of **1**, **5a**, and **5b** with time (Figure 6) fits to the expressions shown in eqs 2–4, respectively, which rationalize two parallel reactions^28^ in agreement with two different oxidative additions. The values of *k*_**5a**_ and *k*_**5b**_ in the temperature range studied are collected in Table 2. The activation parameters calculated from the corresponding Eyring analysis (Figures S18 and S19) are Δ*H*^⧧^ = 13.2 ± 1.2 kcal mol^–1^, Δ*S*^⧧^ = −28.4 ± 4.1 cal K^–1^ mol^–1^, and Δ*G*_298_^⧧^ = 21.7 ± 2.4 kcal mol^–1^ for **5a** and Δ*H*^⧧^ = 14.5 ± 1.2 kcal mol^–1^, Δ*S*^⧧^ = −24.5 ± 4.4 cal K^–1^ mol^–1^, and Δ*G*_298_^⧧^ = 21.8 ± 2.6 kcal mol^–1^ for **5b**.
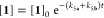
2
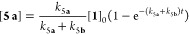
3
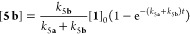
4

**Table 2:** Rate Constants *k*_**5a**_ and *k*_**5b**_ (s^–1^) for the Reaction of **1** with Dichloromethane

*T* (K)	*k*_**5a**_ (s^–1^)	*k*_**5b**_ (s^–1^)
268	(6.2 ± 0.3) × 10^–5^	(3.6 ± 0.3) × 10^–5^
273	(8.3 ± 0.5) × 10^–5^	(6.4 ± 0.6) × 10^–5^
278	(1.2 ± 0.6) × 10^–4^	(8.0 ± 0.5) × 10^–5^
288	(3.5 ± 0.2) × 10^–4^	(2.6 ± 0.3) × 10^–4^
298	(7.6 ± 0.9) × 10^–4^	(6.2 ± 0.9) × 10^–4^

The results of the kinetic analysis prove the existence of two independent oxidative additions of dichloromethane to **1** and confirm the activation barrier sequence qualitatively deduced. In this context, it should be mentioned that the activation energy sequence provided by this study agrees nicely with the sequence built with the C–Cl bond dissociation energies (kcal mol^–1^) previously reported for the employed organic halides:^29^ benzyl chloride (71.7 ± 1.1) < dichloromethane (80.8 ± 0.8) < 2-chloropyridine (90.5 ± 3.5) < chlorobenzene (95.5 ± 3.5). This suggests that the rate of the oxidative addition of organic chlorides to **1** significantly depends upon the strength of the C–Cl bond.

### Five-Coordinate Rhodium(III) Complexes

**Scheme 4:**
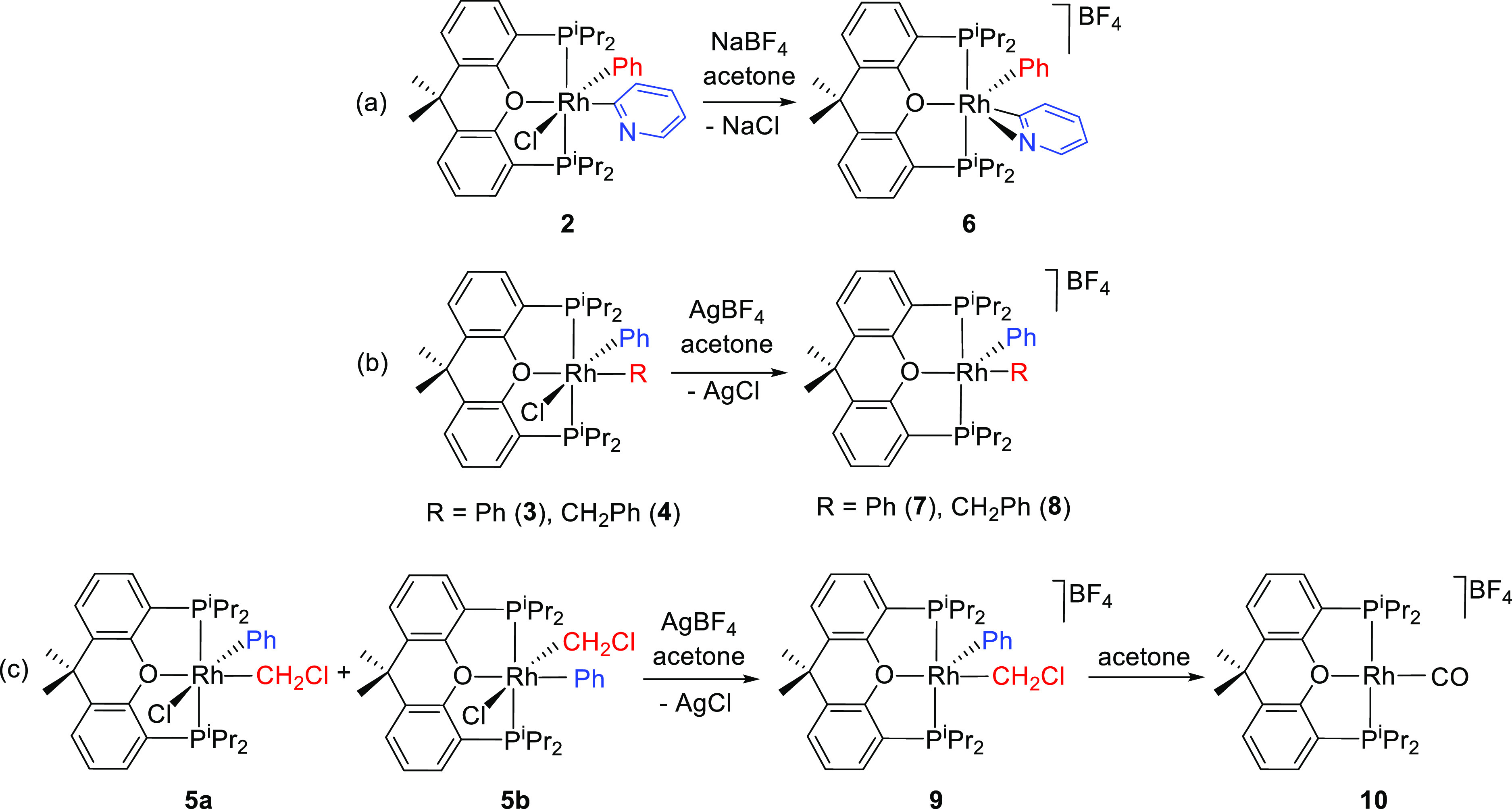
Abstraction of the Chloride Ligand

Complexes **2–5** are stable in fluorobenzene, at 80 °C, for at least 1 week. Reductive C–C elimination was not observed in any case, which can be in principle attributed to the six-coordinate character of these compounds and a low tendency to dissociate the chloride ligand.^11a,16^ In view of it, we decided its abstraction with NaBF_4_ in the case of **2** and AgBF_4_ for **3–5**, in acetone, at room temperature. In contrast to Ag^+^, the Na^+^ ion prevents pyridine–cation interactions that could complicate the abstraction. Three different behaviors are observed depending on the organic halide added to **1** (Scheme 4): (a) 2-chloropyridine (**2**), (b) chlorobenzene (**3**) and benzyl chloride (**4**), and (c) dichloromethane (**5**).

**Figure 7:**
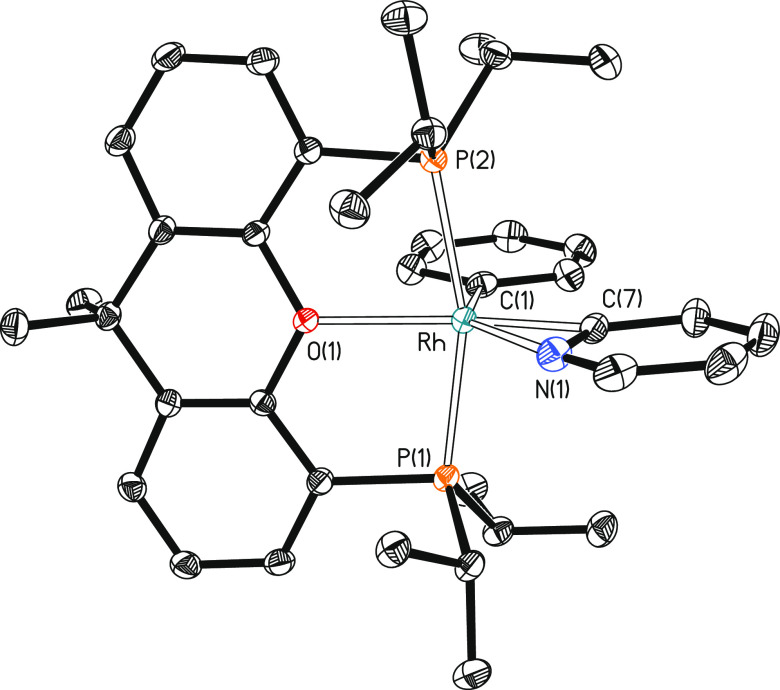
Molecular diagram of the cation of complex **6** (ellipsoids shown at 50% probability). All hydrogen atoms are omitted for clarity. Selected bond distances (Å) and angles (°): Rh–P(1) = 2.3177(11), Rh–P(2) = 2.3071(10), Rh–C(1) = 2.0133(17), Rh–C(7) = 1.9495(17), Rh–N(1) = 2.3483(16), Rh–O(1) = 2.2310(14); P(1)-Rh-P(2) = 163.201(16), O(1)-Rh-C(1) = 91.94(6), O(1)-Rh-C(7) = 160.07(6), C(1)-Rh-C(7) = 107.93(7), N(1)-Rh-C(7) = 34.49(6), O(1)-Rh-M = 141.21(6), C(1)-Rh-M = 126.82(6), where M is the midpoint of the C(7)–N(1) bond.

Treatment of the acetone solutions of **2** with 1.0 equiv of NaBF_4_ leads to the salt [RhPh{η^2^-C,N-(NC_5_H_4_)}{κ^3^-P,O,P-[xant(P^i^Pr_2_)_2_]}]BF_4_ (**6**), where the metal center of the cation saturates its electron deficiency by means of the coordination of the nitrogen atom of the pyridyl group. The salt was isolated as a white solid in 90% yield and characterized by X-ray diffraction analysis. The structure (Figure 7) proves the η^2^-*C*,*N*-coordination of the pyridyl group. Such a coordination mode is relatively usual for early metals^30^ but is very rarely observed in complexes of platinum group metals.^31^ It generates a 3e-donor ligand. Thus, the coordination polyhedron around the metal center can be described as a trigonal bipyramid with inequivalent angles of 91.94(6) (O(1)-Rh-C(1)), 126.82(6) (C(1)-Rh-M), and 141.21(6) (O(1)-Rh-M) in the Y-shaped equatorial plane, which is formed by the oxygen atom of the diphosphine (O(1)), the metalated carbon atom of the phenyl ligand (C(1)), and the midpoint of the pyridyl C(7)–N(1) bond (M). The NMR spectra of the cation (Figures S43–S45) in dichloromethane-*d*_2_ are consistent with the structure shown in Figure 7. The ^31^P{^1^H} spectrum shows a doublet (^1^*J*_P–Rh_ = 113 Hz) at 37.2 ppm, in agreement with the equivalence of the P^i^Pr_2_ groups. In the ^13^C{^1^H} spectrum, the resonances assigned to the metalated carbon atoms are observed at 158.0 (pyridyl) and 135.4 (Ph) ppm, as doublets of triplets with C–Rh and C–P coupling constants of 34 and 44 Hz and 8 and 9 Hz, respectively.

**Figure 8:**
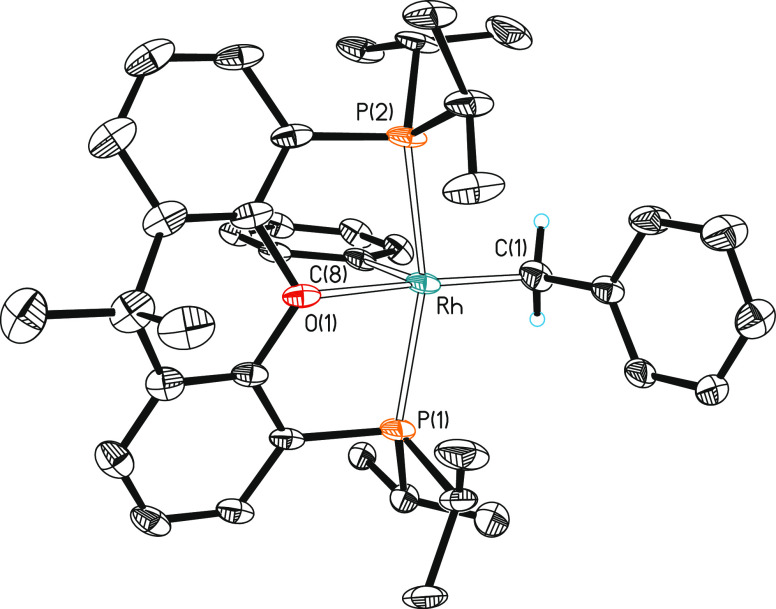
Molecular diagram of the cation of complex **8** (ellipsoids shown at 30% probability). All hydrogen atoms (except those of the CH_2_ moiety) are omitted for clarity. Selected bond distances (Å) and angles (°): Rh–P(1) = 2.3283(10), Rh–P(2) = 2.3351(10), Rh–C(1) = 2.058(5), Rh–C(8) = 2.021(4), Rh–O(1) = 2.307(3); P(1)-Rh-P(2) = 159.71(5), O(1)-Rh-C(1) = 170.28(13), O(1)-Rh-C(8) = 97.10(14), C(1)-Rh-C(8) = 92.62(17).

The abstraction of the chloride ligand of **3** and **4** with AgBF_4_ affords salts [RhPhR{κ^3^-P,O,P-[xant(P^i^Pr_2_)_2_]}]BF_4_ (R = Ph (**7**), CH_2_Ph (**8**)), which were isolated as yellow solids in almost quantitative yields. The five-coordinate unsaturated character of the cations, achieved in spite of the coordinating ability of the anion of the salts^32^ and the reaction solvent is noticeable. Particularly, remarkable is that of cation of **8**, which prefers to coordinate the benzyl group as κ^1^-C instead of the usual benzoallyl form for unsaturated centers.^33^ The unsaturated nature of the cation of **8** was confirmed by X-ray diffraction analysis. Figure 8 gives a view of the structure, which proves the κ^1^-C coordination of the benzyl group. The polyhedron around the rhodium atom can be described as a distorted square pyramid with the phenyl ligand, displaying the strongest trans influence,^27^ at the apex. The benzyl group lies at the base disposed trans to the oxygen atom of the diphosphine (C(1)-Rh-O(1) = 170.28(13)°). In solution, the cations only have a rigid structure at low temperatures. At room temperature, the C-donor ligands undergo a position exchange involving sequential shifts of about 90° in the perpendicular plane to the P–Rh–P direction (see b in Scheme 3). Thus, at room temperature, the ^13^C{^1^H} NMR spectrum of **7** in dichloromethane-*d*_2_ shows a doublet (^1^*J*_C–Rh_ = 41 Hz) of triplets (^2^*J*_C–P_ = 8 Hz) at 141.7 ppm, corresponding to the metalated carbon atoms of the phenyl groups (Figure S48). This signal splits into two resonances at 146 and 138 ppm in the spectrum at 183 K (Figure S49). In the ^13^C{^1^H} spectrum of **8**, at 233 K, the resonances due to metalated carbon atoms are observed at 131.1 (Ph) and 22.6 (CH_2_Ph) ppm (Figure S52).

The addition of 1.0 equiv of AgBF_4_ to acetone solutions of the isomeric mixture of **5a–5b** produces the abstraction of the chloride ligand to initially afford the salt [RhPh(CH_2_Cl){κ^3^-P,O,P-[xant(P^i^Pr_2_)_2_]}]BF_4_ (**9**), a chloromethyl counterpart of **7** and **8**. In a consistent manner with them, its ^13^C{^1^H} NMR spectrum (Figure S55) at 253 K displays two doublets of triplets at 135.1 (^1^*J*_C–Rh_ = 44 Hz, ^2^*J*_C–P_ = 9 Hz) and 45.5 (^1^*J*_C–Rh_ = 34 Hz, ^2^*J*_C–P_ = 7 Hz) ppm, corresponding to the metalated carbon atoms of the phenyl and chloromethyl ligands, respectively. However, in contrast to **7** and **8**, the cation of **9** is unstable in acetone, transforming into the carbonyl derivative [Rh(CO){κ^3^-P,O,P-[xant(P^i^Pr_2_)_2_]}]BF_4_ (**10**), as a consequence of the rhodium-promoted solvent decarbonylation. At 70 °C, the metal carbonylation is completed after 24 h. Salt **10** was isolated as a yellow solid in 87% yield. The presence of the carbonyl group at the cation is strongly supported by the IR, which contains a characteristic strong ν(CO) band at 1978 cm^–1^, and the ^13^C{^1^H} spectrum (Figure S58) shows the expected CO resonance at 191.5 ppm as a doublet of triplets with C–Rh and C–P coupling constants of 86 and 14 Hz. The metal-mediated decarbonylation of aldehydes is a well-known and trivial reaction,^34^ but the carbonyl abstraction from ketones is only rarely observed with very particular systems.^35^

### C–C Reductive Elimination Reactions

**Scheme 5:**
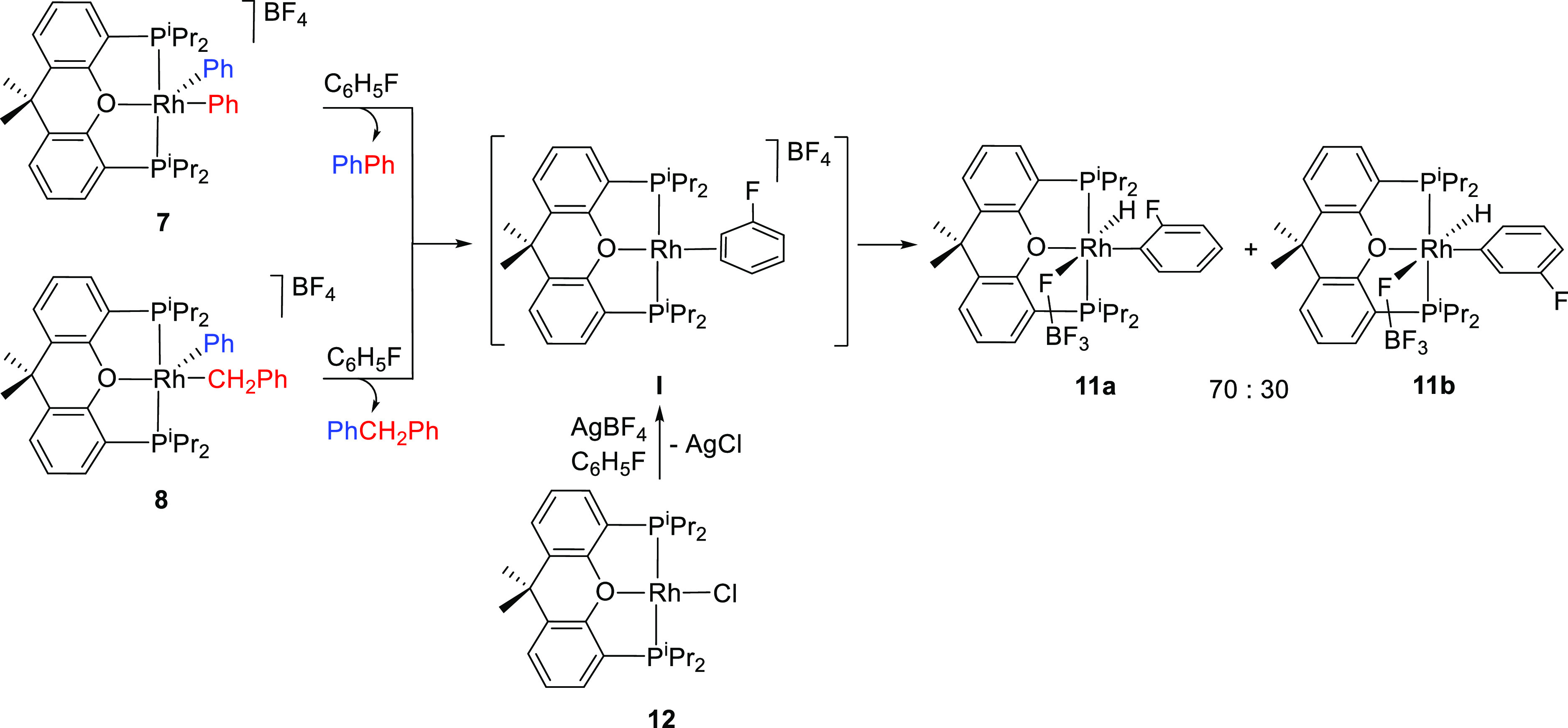
C–C Reductive Elimination Reactions

The electron saturation of the metal center of **6** prevents the reductive elimination of 2-phenylpyridine. Complex **6** is stable in fluorobenzene, at 80 °C, for at least 2 weeks. In contrast to the latter, the unsaturated compounds **7** and **8** eliminate biphenyl and benzylbenzene, respectively, under the same conditions. The resulting solvated fragment [Rh(η^2^-C_6_H_5_F){κ^3^-P,O,P-[xant(P^i^Pr_2_)_2_]}]BF_4_ (**I**) rapidly activates a C–H bond of the coordinated solvent^36^ to give a 7:3 mixture of the *ortho*- and *meta*-fluorophenyl isomers RhH(*o*-C_6_H_4_F)(κ^1^-FBF_3_){κ^3^-P,O,P-[xant(P^i^Pr_2_)_2_]} (**11a**) and RhH(*m*-C_6_H_4_F)(κ^1^-FBF_3_){κ^3^-P,O,P-[xant(P^i^Pr_2_)_2_]} (**11b**). The transformation of **7** into the mixture of **11a** and **11b** is quantitative after 5 days, whereas only 2 days are necessary to convert **8** into the isomeric mixture. On the other hand, the same mixture is also rapidly formed when the chloride ligand of the square-planar rhodium(I) complex RhCl{κ^3^-P,O,P-[xant(P^i^Pr_2_)_2_]} (**12**) is abstracted with AgBF_4_, in fluorobenzene, at room temperature (Scheme 5).

**Figure 9:**
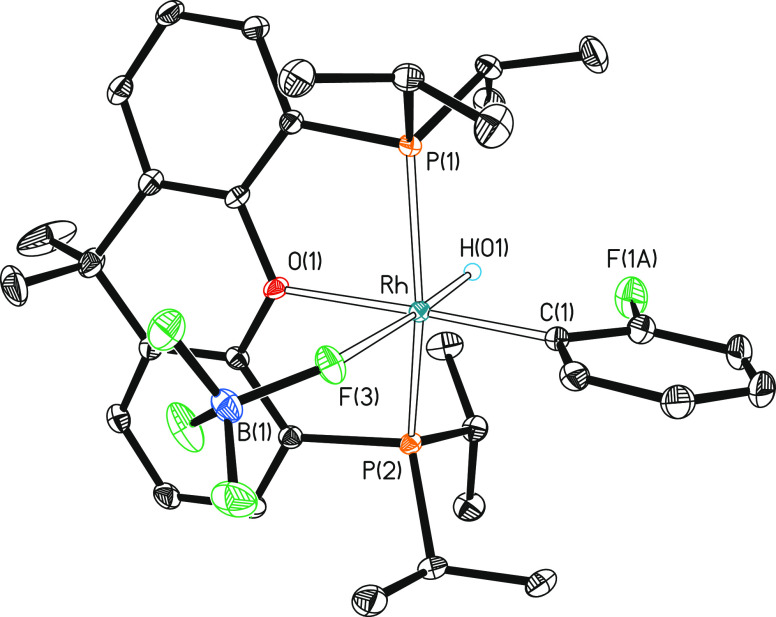
Molecular diagram of complex **11a** (ellipsoids shown at 50% probability). All hydrogen atoms (except the hydride) are omitted for clarity. Selected bond distances (Å) and angles (°): Rh–P(1) = 2.2964(4), Rh–P(2) = 2.2912(4), Rh–C(1) = 2.0009(13), Rh–O(1) = 2.2023(9), Rh–F(3) = 2.3551(9); P(1)-Rh-P(2) = 156.501(13), O(1)-Rh-C(1) = 177.90(5), H(01)-Rh-F(3) = 175.4(7), H(01)-Rh-C(1) = 84.6(6), C(1)-Rh-F(3) = 95.44(5).

The coordination of the [BF_4_]^−^ anion to the metal center of **11a** and **11b** in the solid state was revealed by the FT-IR–ATR of the mixture, which displays the characteristic absorptions for a BF_4_-group with *C*_*s*_ symmetry^32^ at 1095, 953, and 745 cm^–1^ and the X-ray structure of **11a**, which proves the coordination index of six for its metal center (Figure 9). Thus, the polyhedron around the rhodium atom can be idealized as an octahedron with the diphosphine disposed in *mer*-fashion and a perpendicular plane to the P–Rh–P direction containing the hydride ligand disposed trans to the monodentate [BF_4_]^−^ anion and the fluorophenyl group situated trans to the oxygen atom of the pincer. In acetone solution, both isomers dissociate the [BF_4_]^−^ anion. This is strongly supported by the ^19^F{^1^H} NMR spectrum (Figure S62), which contains only one [BF_4_]^−^ resonance at −151.4 ppm, whereas the ^1^H and ^31^P{^1^H} NMR spectra (Figures S59 and S60) do not display spin coupling with ^19^F. Thus, even at 193 K, the first of them shows the hydride resonances as doublets of triplets at −18.95 (^1^*J*_H–Rh_ = 30.6 Hz, ^2^*J*_H–P_ = 12.9 Hz) ppm for **11a** and at −19.92 (^1^*J*_H–Rh_ = 35.5 Hz, ^2^*J*_H–P_ = 13.4 Hz) ppm for **11b**. The second one, for its part, displays a single doublet for each isomer, at 43.6 (^1^*J*_P–Rh_ = 111 Hz) ppm for **11a** and at 40.9 (^1^*J*_P–Rh_ = 115 Hz) ppm for **11b**.

The extremely rapid formation of the isomeric mixture of **11a** and **11b**, by abstraction of the chloride ligand of **12** in fluorobenzene, indicates that the C–H bond activation of the coordinated fluorobenzene of **I** is much faster than C–C reductive elimination from the five-coordinate cations. This is consistent with the nonobservation of such an intermediate during the transformations of **7** and **8** into the isomeric mixture and points out that the C–C reductive elimination is the rate-determining step of the processes and therefore the step from which the activation parameters depend. Such transformations were followed by ^31^P{^1^H} NMR spectroscopy. Decreases in **7** and **8** are first-order reactions, which can be described according to eq 5, where [**M**]_0_ is the initial concentrations of the five-coordinate cations, whereas [**M**] represents the concentrations at the time *t*. The values of *k*_R_ in the temperature range studied are collected in Table 3. The activation parameters for the respective C–C reductive eliminations, obtained from the corresponding Eyring analysis (Figures S25 and S30), are Δ*H*^⧧^ = 24.2 ± 3.4 kcal mol^–1^, Δ*S*^⧧^ = −13.0 ± 9.9 cal K^–1^ mol^–1^, and Δ*G*_298_^⧧^ = 28.1 ± 6.4 kcal mol^–1^ for the reductive elimination of biphenyl and Δ*H*^⧧^ = 6.2 ± 1.5 kcal mol^–1^, Δ*S*^⧧^ = −31.4 ± 4.4 cal K^–1^ mol^–1^, and Δ*G*_298_^⧧^ = 15.6 ± 2.9 kcal mol^–1^ for the reductive elimination of benzylbenzene. The lower activation enthalpy for the C(sp^3^)–C(sp^2^) reductive elimination respecting the C(sp^2^)–C(sp^2^) coupling is consistent with the smaller dissociation energy of the C(sp^3^)–C(sp^2^) bond of benzylbenzene with regard to the dissociation energy of the C(sp^2^)–C(sp^2^) single bond of biphenyl (91.7 ± 2.0 vs 114.4 ± 1.5 kcal mol^–1^).^29^ The marked negative values of the activation entropies agree well with the concerted character of the reductive eliminations, occurring through geometrically highly oriented transition states; more oriented for the benzyl–phenyl coupling than for the phenyl–phenyl one, as a consequence of the higher directionality of the sp^3^ orbital of the benzyl group in relation to the phenyl sp^2^ orbital. The combination of both factors gives rise to a C(sp^3^)–C(sp^2^) reductive coupling faster than the C(sp^2^)–C(sp^2^) bond formation. Although a most demanding orientation requirement is needed for the C(sp^3^)–C(sp^2^) coupling than for the C(sp^2^)–C(sp^2^) bond formation, the energetic effort for the pregeneration of the C(sp^3^)–C(sp^2^) bond is smaller. These observations represent an inversion for the pair C(sp^2^)–C(sp^2^):C(sp^3^)–C(sp^2^) in the order C(sp^2^)–C(sp^2^) > C(sp^3^)–C(sp^2^) > C(sp^3^)–C(sp^3^), theoretically established for the reductive elimination preference.^14b,14c^ Previously, notable inversions had been observed for the pair C(sp^3^)–C(sp^2^):C(sp^3^)–C(sp^3^) in competitive experiments.^13a,13e,37^
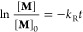
5

**Table 3:** Rate Constants (*k*_R_, s^–1^) for the C–C Reductive Elimination Processes from Complexes **7** and **8**

complex **7**		complex 8	
*T* (K)	*k*_R_ (s^–1^)	*T* (K)	*k*_R_ (s^–1^)
343	(3.6 ± 0.6) × 10^–6^	338	(1.1 ± 0.3) × 10^–5^
348	(6.1 ± 0.6) × 10^–6^	353	(1.8 ± 0.8) × 10^–5^
353	(1.1 ± 0.1) × 10^–5^	358	(1.9 ± 0.4) × 10^–5^
358	(1.9 ± 0.4) × 10^–5^	363	(2.3 ± 0.7) × 10^–5^
363	(2.6 ± 0.4) × 10^–5^		

### Reductive Elimination of Fluorobenzene and Deprotonation of the Isomeric Mixture

**Scheme 6:**
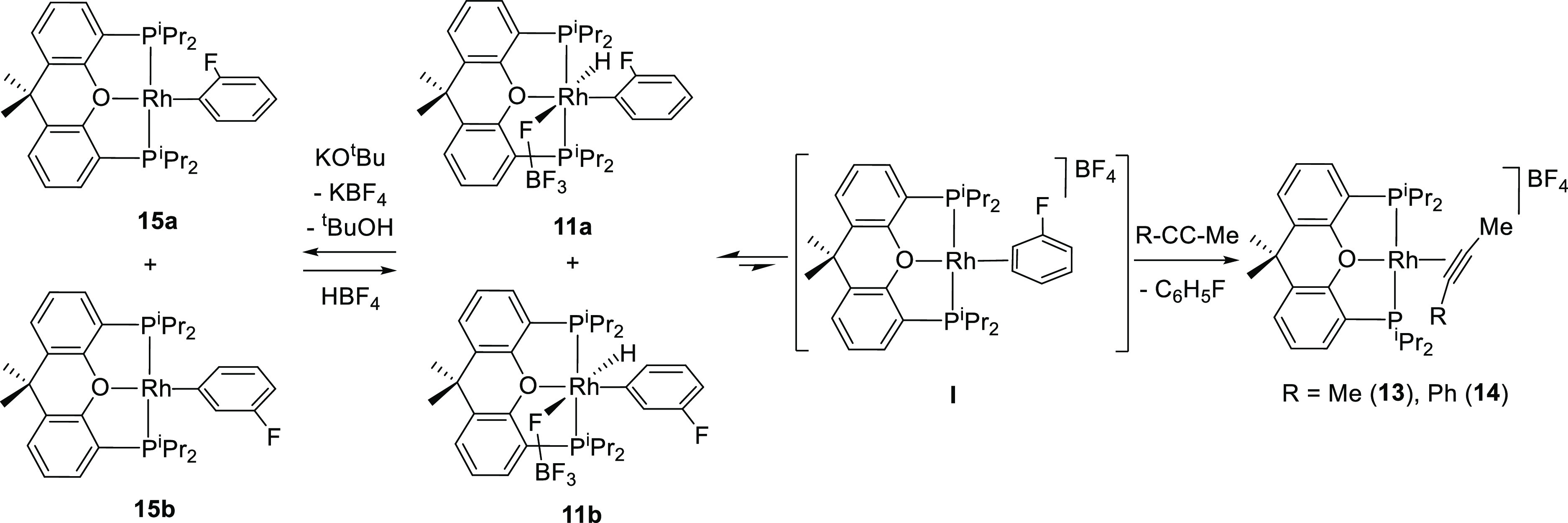
Reactions of **11a** and **11b**

The activation barrier for the intramolecular reductive elimination of fluorobenzene in **11a** and **11b** is not significantly different from the activation barrier for the C–H bond oxidative addition in **I**, since in solution hydride-rhodium(III)-aryl isomers are in equilibrium with spectroscopically nondetected amounts of their precursor intermediate. This is strongly supported by the reactions of the isomeric mixture with internal alkynes such as 2-butyne and 1-phenyl-1-propyne (Scheme 6). Such hydrocarbons do not undergo the insertion of the C–C triple bond into the Rh–H bond of the rhodium(III) isomers but provoke the displacement of fluorobenzene, to form the π-alkyne derivatives [Rh(η^2^-MeC≡CR){κ^3^-P,O,P-[xant(P^i^Pr_2_)_2_]}]BF_4_ (R = Me (**13**), Ph (**14**)). These compounds were isolated as yellow solids in almost quantitative yield. Their ^31^P{^1^H} and ^13^C{^1^H} NMR spectra (Figures S64–S68) in acetone-*d*_6_ reveal that the triple bond of the alkynes lies in a perpendicular plane to the P–Rh–P direction, in agreement with the X-ray structure previously reported for the related cation [Rh(η^2^-PhC≡CPh){κ^3^-P,O,P-[xant(PPh_2_)_2_]}]^+^.^18e^ Thus, the ^31^P{^1^H} spectra show doublets (^1^*J*_P–Rh_ ≈ 124 Hz) at about 35 ppm, for the equivalent P^i^Pr_2_ groups, whereas the ^13^C{^1^H} spectra display doublets (^1^*J*_C–Rh_ ≈ 16 Hz) of triplets (^2^*J*_C–P_ ≈ 4 Hz) for the C(sp)–carbon atoms, at 56.3 ppm for **13** and at 72.8 (CMe) and 61.2 (CPh) ppm for **14**.

The hydride ligand of **11a** and **11b** is fairly acidic, in agreement with the last step of the cycle shown in Scheme 1. Thus, the addition of 1.0 equiv of KO^*t*^Bu to acetone solutions of the isomeric mixture produces the abstraction of the hydride ligand and the formation of the corresponding mixture of the previously reported square-planar rhodium(I)-aryl derivatives Rh(*o*-C_6_H_4_F){κ^3^-P,O,P-[xant(P^i^Pr_2_)_2_]} (**15a**) and Rh(*m*-C_6_H_4_F){κ^3^-P,O,P-[xant(P^i^Pr_2_)_2_]} (**15b**).^10g,10i^ The reduction is reversible, and the addition of 1.0 equiv of HBF_4_·OEt_2_ to fluorobenzene solutions of the rhodium(I) isomeric mixture regenerates the rhodium(III) one.

## Concluding Remarks

This study has revealed that the oxidative addition of organic chlorides to the square-planar rhodium(I)-phenyl complex RhPh{κ^3^-P,O,P-[xant(P^i^Pr_2_)_2_]} in the majority of the cases involves a cis addition of the C–Cl bond. Only for some particular organic chlorides, such as dichloromethane, the trans addition is competitive. The formation of the resulting rhodium(III) species is kinetically controlled by the C–Cl bond dissociation energy.

The coordinatively saturated compounds generated from the oxidative additions are stable toward a subsequent C–C reductive elimination. The abstraction of the chloride from the metal center gives rise to unsaturated five-coordinate species, displaying square pyramid structures with the coordinated C-donor ligands at basal and axial positions. In contrast to the six-coordinate precursors, these compounds undergo C–C reductive coupling, with some noticeable exceptions as complexes bearing 2-pyridyl and methylchloride. The former backs to stabilize the metal center by coordination of the nitrogen atom, whereas the second one has the ability to promote the decomposition of the complex by means of the decarbonylation of solvents such as acetone. The activation energy of the reductive elimination depends upon the formed C–C bond. Thus, the C(sp^3^)–C(sp^2^) reductive couplings are faster than the C(sp^2^)–C(sp^2^) bond formation. In spite of that a most demanding orientation requirement is needed for the C(sp^3^)–C(sp^2^) coupling than for the C(sp^2^)–C(sp^2^) bond formation, the energetic effort for the pregeneration of the C(sp^3^)–C(sp^2^) bond is smaller. In fluorobenzene, the reductive coupling is followed by a fast oxidative addition of a C–H bond of the solvent, which generates a fairly acidic hydride-rhodium(III)-aryl derivative. The deprotonation of the latter affords a new square planar rhodium(I)-aryl complex.

The reactions performed in this study starting from a square-planar rhodium(I)-aryl complex include C–Cl oxidative addition of organic chlorides, halide abstraction from the resulting six-coordinate rhodium(III) derivative, C–C reductive coupling between the initial aryl ligand and the added organic group, oxidative addition of a C–H bond of a new arene, and deprotonation of the generated hydride-rhodium(III)-aryl species to form a new square planar rhodium(I)-aryl derivative. They constitute a cycle of stoichiometric elemental reactions, which defines the direct arylation promoted by a redox-pair Rh(I)–Rh(III). The results obtained suggest that the key steps of such arylation should be the C–Cl oxidative addition and the C–C reductive elimination. From a kinetic point of view, the former is controlled by the dissociation energy of the added bond, while the second one is governed by the dissociation energy of the formed bond. The weakest C–Cl bond is added faster, while the weakest C–C bond is also formed faster.

## Experimental Section

### General Information

All reactions were carried out with exclusion of air using Schlenk-tube techniques or in a glovebox. Instrumental methods and X-ray details are given in the Supporting Information. In the NMR spectra (Figures S31–S68), the chemical shifts (in ppm) are referenced to residual solvent peaks (^1^H, ^13^C{^1^H}) or external 85% H_3_PO_4_ (^31^P{^1^H}), while *J* and *N* (*N* = *J*_P–H_ + *J*_P′–H_ for ^1^H and *N* = *J*_P–C_ + *J*_P′–C_ for ^13^C{^1^H}) are given in hertz. RhPh{κ^3^-P,O,P-[xant(P^i^Pr_2_)_2_]} (**1**)^10g^ and RhCl{κ^3^-P,O,P-[xant(P^i^Pr_2_)_2_]} (**12**)^21a^ were prepared by the published methods.

#### Reaction of RhPh{κ^3^-P,O,P-[xant(P^i^Pr_2_)_2_]} (**1**) with 2-Chloropyridine: Preparation of Rh(Ph)(2-pyridyl)Cl{κ^3^-P,O,P-[xant(P^i^Pr_2_)_2_]} (**2**)

A solution of **1** (123 mg, 0.20 mmol) in 2-chloropyridine (3 mL) was stirred at 50 °C during 48 h. The resulting solution was evaporated to dryness to afford a yellowish residue. The addition of pentane (4 mL) afforded a white solid that was washed with pentane (2 × 2 mL) and dried in vacuo. Yield: 81 mg (56%). Anal. Calcd for C_38_H_49_ClNOP_2_Rh: C, 62.00; H, 6.71; N, 1.90. Found: C, 62.22; H, 6.42; N, 2.16. HRMS (electrospray, *m*/*z*): calcd for C_38_H_49_NOP_2_Rh [M – Cl]^+^, 700.2339; found, 700.2342. IR (cm^–1^): ν(C=N) 1562 (m), ν(C–O–C) 1192 (m). ^1^H NMR (300.13 MHz, C_6_D_6_, 298 K): δ 8.54 (d, ^3^*J*_H–H_ = 2.8, 1H, py), 8.44 (d, ^3^*J*_H–H_ = 7.9, 1H, py), 8.33 (d, ^3^*J*_H–H_ = 7.4, 1H, Ph), 7.31–6.59 (m, 11H, 3H Ph + 2H py + 6H CH-arom POP), 6.38 (t, ^3^*J*_H–H_ = 7.0, 1H, Ph), 3.46 (m, 2H, PC*H*(CH_3_)_2_), 2.63 (m, 2H, PC*H*(CH_3_)_2_), 1.48 (s, 3H, CH_3_), 1.40–1.14 (m, 15H, 12H PCH(C*H*_3_)_2_ + 3H CH_3_), 1.02 (dvt, ^3^*J*_H–H_ = 7.3, *N* = 14.7, 6H, PCH(C*H*_3_)_2_), 0.46 (dvt, ^3^*J*_H–H_ = 6.8, *N* = 13.8, 6H, PCH(C*H*_3_)_2_). ^13^C{^1^H}-apt NMR (75.48 MHz, C_6_D_6_, 298 K): δ 173.9 (dt, ^1^*J*_C–Rh_ = 40, ^2^*J*_C–P_ = 6, Rh–C py), 154.2 (vt, *N* = 12, C-arom POP), 146.0 (s, CH py), 143.7 (dt, ^1^*J*_C–Rh_ = 34, ^2^*J*_C–P_ = 10, Rh–C Ph), 141.6 (s, CH Ph), 136.8 (t, ^3^*J*_C–P_ = 4, CH py), 136.4 (s, CH Ph), 133.5 (s, CH-arom POP), 132.0 (vt, *N* = 5, C-arom POP), 130.9 (s, CH py), 128.1 (s, CH-arom POP), 127.9 (s, CH Ph), 125.7 (s, CH Ph), 124.4 (s, CH-arom POP), 123.8 (vt, *N* = 24.1, C-arom POP), 122.8 (s, CH Ph), 117.5 (s, CH py), 35.3 (s, C(*C*H_3_)_2_), 34.7 (s, *C*(CH_3_)_2_), 28.6 (s, C(*C*H_3_)_2_), 27.6 (vt, *N* = 21, P*C*H(CH_3_)_2_), 25.6 (dvt, *N* = 22, ^2^*J*_C–Rh_ = 2.0, P*C*H(CH_3_)_2_), 21.7, 21.3, 20.0, 19.7 (all s, PCH(*C*H_3_)_2_). ^31^P{^1^H} NMR (121.49 MHz, C_6_D_6_, 298 K): δ 27.7 (d, ^1^*J*_Rh–P_ = 119).

#### Reaction of RhPh{κ^3^-P,O,P-[xant(P^i^Pr_2_)_2_]} (**1**) with Chlorobenzene: Preparation of RhPh_2_Cl{κ^3^-P,O,P-[xant(P^i^Pr_2_)_2_]} (**3**)

A solution of **1** (100 mg, 0.16 mmol) in chlorobenzene (3 mL) was stirred at 90 °C during 48 h. The resulting solution was evaporated to dryness to afford a yellow residue. The addition of pentane (4 mL) afforded a yellowish white solid that was washed with pentane (2 × 2 mL) and dried in vacuo. Yield: 89.5 mg (76%). Anal. Calcd for C_39_H_50_ClOP_2_Rh: C, 63.72; H, 6.86. Found: C, 63.35; H, 7.06. HRMS (electrospray, *m*/*z*): calcd for C_39_H_50_OP_2_Rh [M – Cl]^+^, 699.2392; found, 699.2397. IR (cm^–1^): ν(C–O–C) 1187 (m). ^1^H NMR (400.16 MHz, C_6_D_6_, 298 K): δ 8.55 (d, ^3^*J*_H–H_ = 7.9, 2H, Ph), 8.25 (d, ^3^*J*_H–H_ = 7.1, 1H, Ph), 7.30–7.02 (m, 9H, 4H CH-arom POP + 5H Ph), 6.94–6.83 (m, 3H, 2H CH-arom POP + 1H Ph), 6.68 (dt, ^3^*J*_H–H_ = 1.4, ^3^*J*_H–H_ = 7.6, 1H, Ph), 3.47 (m, 2H, PC*H*(CH_3_)_2_), 2.49 (m, 2H, PC*H*(CH_3_)_2_), 1.39 (dvt, ^3^*J*_H–H_ = 7.3, *N* = 14.9, 6H, PCH(C*H*_3_)_2_), 1.33 (s, 3H, CH_3_), 1.23 (s, 3H, CH_3_), 1.18 (dvt, ^3^*J*_H–H_ = 7.3, *N* = 14.9, 6H, PCH(C*H*_3_)_2_), 0.66 (dvt, ^3^*J*_H–H_ = 6.9, *N* = 13.3, 6H, PCH(C*H*_3_)_2_), 0.60 (dvt, ^3^*J*_H–H_ = 7.0, *N* = 14.2, 6H, PCH(C*H*_3_)_2_). ^13^C{^1^H}-apt NMR (75.48 MHz, C_6_D_6_, 298 K): δ 155.4 (vt, *N* = 11, C-arom POP), 152.7 (dt, ^1^*J*_C–Rh_ = 33, ^2^*J*_C–P_ = 8, Rh–*C* Ph), 146.4 (dt, ^1^*J*_C–Rh_ = 39, ^3^*J*_C–P_ = 9, Rh–*C* Ph), 144.5, 142.5, 137.6 (all s, CH Ph), 133.2 (s, CH-arom POP), 132.7 (vt, *N* = 5, C-arom POP), 127.9 (s, CH-arom POP), 127.6, 125.7, 125.1 (all s, CH Ph), 124.3 (s, C-arom POP), 124.0 (s, CH-arom POP), 122.6 (s, CH Ph), 34.8 (s, *C*(CH_3_)_2_), 33.7, 27.8 (both s, C(*C*H_3_)_2_), 26.8 (vt, *N* = 21.7, P*C*H(CH_3_)_2_), 25.7 (vt, *N* = 18, P*C*H(CH_3_)_2_), 21.3, 20.4, 19.9, 19.6 (all s, PCH(*C*H_3_)_2_). ^31^P{^1^H} NMR (161.99 MHz, C_6_D_6_, 298 K): δ 26.5 (d, ^1^*J*_P–Rh_ = 114).

#### Reaction of RhPh{κ^3^-P,O,P-[xant(P^i^Pr_2_)_2_]} (**1**) with Benzyl Chloride: Preparation of RhPh(CH_2_Ph)Cl{κ^3^-P,O,P-[xant(P^i^Pr_2_)_2_]} (**4**)

A solution of **1** (105 mg, 0.17 mmol) in toluene (3 mL) was treated with benzyl chloride (19 μL, 0.17 mmol) and the resulting solution was stirred at room temperature for 5 min. After this time, it was evaporated to dryness to afford a yellowish residue. The addition of pentane (4 mL) afforded a white solid that was washed with pentane (2 × 2 mL) and dried in vacuo. Yield: 101 mg (80%). Anal. Calcd for C_40_H_52_ClOP_2_Rh: C, 64.13; H, 7.00. Found: C, 63.75; H, 7.12. HRMS (electrospray, *m*/*z*): calcd for C_40_H_52_OP_2_Rh [M – Cl]^+^, 713.2543; found, 713.2557. IR (cm^–1^): ν(C–O–C) 1192 (m). ^1^H NMR (300.13 MHz, C_6_D_6_, 298 K): δ 8.67–8.45 (m, 3H, 1H Ph + 2H CH_2_Ph), 7.34–6.74 (m, 11H, 2H Ph + 3H CH_2_Ph + 6H CH-arom POP), 6.66 (d, ^3^*J*_H–H_ = 7.4, 1H, Ph), 6.36 (t, ^3^*J*_H–H_ = 7.3, 1H, Ph), 5.02 (dt, ^2^*J*_H–Rh_ = 3.2, ^3^*J*_H–P_ = 3.6, 2H, RhC*H*_2_Ph), 3.39 (m, 2H, PC*H*(CH_3_)_2_), 2.22 (m, 2H, PC*H*(CH_3_)_2_), 1.44 (s, 3H, CH_3_), 1.26 (dvt, ^3^*J*_H–H_ = 7.1, *N* = 13.3, 6H, PCH(C*H*_3_)_2_), 1.20 (s, 3H, CH_3_), 1.17 (dvt, ^3^*J*_H–H_ = 6.2, *N* = 13.0, 6H, PCH(C*H*_3_)_2_), 0.85 (dvt, ^3^*J*_H–H_ = 7.5, *N* = 15.0, 6H, PCH(C*H*_3_)_2_), 0.13 (dvt, ^3^*J*_H–H_ = 6.8, *N* = 13.3, 6H, PCH(C*H*_3_)_2_). ^13^C{^1^H}-apt NMR (75.48 MHz, C_6_D_6_, 298 K): δ 155.1 (t, ^3^*J*_C–P_ = 4, C CH_2_Ph), 153.8 (vt, *N* = 10.5, C-arom POP), 141.9 (dt, ^1^*J*_C–Rh_ = 33, ^2^*J*_C–P_ = 11, Rh–C Ph), 140.4 (s, CH Ph), 137.4 (s, CH Ph), 133.2 (s, CH-arom POP), 132.0 (s, C-arom POP), 131.0 (s, CH CH_2_Ph), 128.1 (s, CH CH_2_Ph), 127.9 (s, CH-arom POP), 127.5 (s, CH Ph), 126.1 (s, CH Ph), 125.0 (s, CH CH_2_Ph), 124.2 (s, CH-arom POP), 123.4 (vt, *N* = 25.2, C-arom POP), 122.7 (s, CH Ph), 34.9 (s, C(*C*H_3_)_2_), 34.6 (s, *C*(CH_3_)_2_), 28.5 (s, C(*C*H_3_)_2_), 27.1 (vt, *N* = 19.2, P*C*H(CH_3_)_2_), 26.2 (vt, *N* = 19.7, P*C*H(CH_3_)_2_), 22.9, 20.4, 19.5, 18.2 (all s, PCH(*C*H_3_)_2_), 16.8 (dt, ^1^*J*_C–Rh_ = 29, ^2^*J*_C–P_ = 5, Rh–*C*H_2_Ph). ^31^P{^1^H} NMR (121.49 MHz, C_6_D_6_, 298 K): δ 22.4 (d, ^1^*J*_Rh–P_ = 118).

#### Reaction of RhPh{κ^3^-P,O,P-[xant(P^i^Pr_2_)_2_]} (**1**) with Dichloromethane: Preparation of RhPh(CH_2_Cl)Cl{κ^3^-P,O,P-[xant(P^i^Pr_2_)_2_]} (**5a–5b**)

Complex **1** (70 mg, 0.11 mmol) was dissolved in dichloromethane (3 mL), and the solution was stirred for 5 min at room temperature. The solution was evaporated to dryness to afford a yellow residue. The addition of pentane (4 mL) afforded a whitish solid that was washed with pentane (2 × 2 mL) and dried in vacuo. Yield: 55 mg (69%). Anal. Calcd for C_34_H_47_Cl_2_OP_2_Rh: C, 57.72; H, 6.70. Found: C, 57.31; H, 6.95. HRMS (electrospray, *m*/*z*): calcd for C_34_H_47_ClOP_2_Rh [M – Cl]^+^, 671.1846; found, 671.1855. IR (cm^–1^): ν(C=C) 1568 (w), ν(C–O–C) 1196 (m). ^1^H and ^31^P{^1^H} NMR spectra show the formation of **5a** and **5b** in a 1.1:1 ratio. ^1^H NMR both isomers (300.13 MHz, CD_2_Cl_2_, 298 K): δ 7.89 (d, ^3^*J*_H–H_ = 7.6, 1H, Ph), 7.74 (d, ^3^*J*_H–H_ = 6.6, 2H, Ph), 7.69–7.18 (m, 12H, CH-arom POP), 7.07 (t, ^3^*J*_H–H_ = 7.6, 1H, Ph), 6.92–6.80 (m, 3H, Ph), 6.72 (t, ^3^*J*_H–H_ = 7.0, 1H, Ph), 6.53 (d, ^3^*J*_H–H_ = 8.0, 1H, Ph), 6.38 (t, ^3^*J*_H–H_ = 7.1, 1H, Ph), 5.76 (dt, ^2^*J*_H–Rh_ = 3.2, ^3^*J*_H–P_ = 6.5, 2H, RhC*H*_2_Cl), 4.79 (dt, ^2^*J*_H–Rh_ = 2.0, ^3^*J*_H–P_ = 7.6, 2H, RhC*H*_2_Cl), 3.56, 3.25, 2.88, 2.50 (all m, 2H each, PC*H*(CH_3_)_2_), 1.82, 1.81, 1.62, 1.50 (all s, 3H each, CH_3_), 1.47–1.34 (m, 12H, PCH(C*H*_3_)_2_), 1.30–1.12 (m, 24H, PCH(C*H*_3_)_2_), 0.93 (dvt, ^3^*J*_H–H_ = 7.3, *N* = 14.6, 6H, PCH(C*H*_3_)_2_), 0.38 (dvt, ^3^*J*_H–H_ = 7.2, *N* = 14.1, 6H, PCH(C*H*_3_)_2_). ^13^C{^1^H}-apt NMR both isomers (75.48 MHz, CD_2_Cl_2_, 298 K): δ 154.1 (vt, *N* = 12.7, C-arom POP), 153.6 (vt, *N* = 11.9, C-arom POP), 142.3 (dt, ^1^*J*_C–Rh_ = 34, ^2^*J*_C–P_ = 10, Rh–C Ph), 140.4 (dt, ^1^*J*_C–Rh_ = 37, ^2^*J*_C–P_ = 10, Rh–C Ph), 138.6, 137.7, 136.2 (all s, CH Ph), 134.3 (s, CH-arom POP), 134.2 (s, CH-arom POP), 132.3 (vt, *N* = 6, C-arom POP), 132.2 (vt, *N* = 4, C-arom POP), 129.7, 129.6 (both s, CH-arom POP), 128.4 (s, CH Ph), 126.4 (s, CH Ph), 124.9 (vt, *N* = 5, CH-arom POP), 124.0 (vt, *N* = 5, CH-arom POP), 123.3 (vt, *N* = 22, C-arom POP), 123.0 (s, CH Ph), 122.9 (vt, *N* = 19, C-arom POP), 122.4 (s, CH Ph), 40.7 (dt, ^1^*J*_C–Rh_ = 34, ^2^*J*_C–P_ = 8, Rh–*C*H_2_Cl), 40.2 (dt, ^1^*J*_C–Rh_ = 37, ^2^*J*_C–P_ = 6, Rh–*C*H_2_Cl), 36.6 (s, C(*C*H_3_)_2_), 35.1 (s, *C*(CH_3_)_2_), 34.8, 32.2, 30.6 (all s, C(*C*H_3_)_2_), 28.2 (vt, *N* = 22.7, P*C*H(CH_3_)_2_), 26.6 (vt, *N* = 24, P*C*H(CH_3_)_2_), 26.0 (vt, *N* = 21, P*C*H(CH_3_)_2_), 25.0 (vt, *N* = 19, P*C*H(CH_3_)_2_), 21.8, 21.7, 21.2, 21.0, 20.6, 19.7, 19.6, 19.5 (all s, PCH(*C*H_3_)_2_). ^31^P{^1^H} NMR both isomers (161.99 MHz, CD_2_Cl_2_, 298 K): δ 27.5 (d, ^1^*J*_Rh–P_ = 114), 27.3 (d, ^1^*J*_Rh–P_ = 110).

#### Kinetic Analysis of the Reaction of **1** with 2-Chloropyridine

In the glovebox, an NMR tube was charged with a solution of **1** (20 mg, 0.03 mmol) in 2-chloropyridine (0.5 mL), and a capillary tube filled with a solution of the internal standard (PCy_3_) in toluene-*d*_8_ was placed in the NMR tube. The tube was immediately introduced into an NMR probe preheated at the desired temperature (323, 328, 333, 338, and 343 K), and the reaction was monitored by ^31^P{^1^H} NMR spectroscopy (a delay of 25 s was used) at different intervals of time. The experiments were performed in duplicate. Rate constants were obtained by plotting eq 1. Errors were calculated using the standard deviation data provided by Microsoft Excel.

#### Kinetic Analysis of the Reaction of **1** with Chlorobenzene

In the glovebox, an NMR tube was charged with a solution of **1** (20 mg, 0.03 mmol) in chlorobenzene (0.5 mL), and a capillary tube filled with a solution of the internal standard (PCy_3_) in toluene-*d*_8_ was placed in the NMR tube. The tube was immediately introduced into an NMR probe preheated at the desired temperature (363, 373, 383, 393, and 398 K), and the reaction was monitored by ^31^P{^1^H} NMR spectroscopy (a delay of 25 s was used) at different intervals of time. The experiments were performed in duplicate. Rate constants were obtained by plotting eq 1. Errors were calculated using the standard deviation data provided by Microsoft Excel.

#### Kinetic Analysis of the Reaction of **1** with Dichloromethane

In the glovebox, an NMR tube was charged with a solution of **1** (20 mg, 0.03 mmol) and dichloromethane (41 μL, 0.64 mmol) in toluene-*d*_8_ (0.5 mL), and a capillary tube filled with a solution of the internal standard (PCy_3_) in toluene-*d*_8_ was placed in the NMR tube. The tube was immediately introduced into an NMR probe at the desired temperature (268, 273, 278, 288, and 298 K), and the reaction was monitored by ^31^P{^1^H} NMR spectroscopy (a delay of 25 s was used) at different intervals of time. The experiments were performed in duplicate. Rate constants were obtained from eqs 2–4. Errors were calculated using the standard deviation data provided by Microsoft Excel.

#### Reaction of Rh(Ph)(2-pyridyl)Cl{κ^3^-P,O,P-[xant(P^i^Pr_2_)_2_]} (**2**) with NaBF_4_: Preparation of [RhPh{η^2^-C,N-(NC_5_H_4_)}{κ^3^-P,O,P-[xant(P^i^Pr_2_)_2_]}]BF_4_ (**6**)

A solution of **2** (100 mg, 0.13 mmol) in acetone (3 mL) was treated with NaBF_4_ (15 mg, 0.13 mmol), and the resulting mixture was stirred at room temperature for 1 h. After this time, it was evaporated to dryness to afford a light brown residue and methylene chloride (4 mL) was added. The resulting suspension was filtered through Celite to remove the sodium salts and the solution obtained was evaporated to dryness to afford a yellow residue. The addition of diethyl ether (4 mL) afforded a white solid that was washed with diethyl ether (2 × 2 mL) and dried in vacuo. Yield: 96 mg (90%). Anal. Calcd for C_38_H_49_ BF_4_NOP_2_Rh: C, 57.96; H, 6.27; N, 1.78. Found: C, 57.57; H, 6.41; N, 1.73. HRMS (electrospray, *m*/*z*): calcd for C_38_H_49_NOP_2_Rh [M]^+^, 700.2339; found, 700.2315. IR (cm^–1^): ν(C=N) 1551 (m), ν(C–O–C) 1189 (m), ν(B–F) 1055 (vs). ^1^H NMR (400.13 MHz, CD_2_Cl_2_, 233 K): 8.30 (d, ^3^*J*_H–H_ = 4.5, 1H, py), 8.19 (d, ^3^*J*_H–H_ = 7.9, 1H, py), 8.09 (d, ^3^*J*_H–H_ = 7.7, 1H, Ph), 7.87 (d, ^3^*J*_H–H_ = 7.1, 2H, CH-arom POP), 7.73 (t, ^3^*J*_H–H_ = 7.2, 1H, py), 7.48 (t, ^3^*J*_H–H_ = 7.6, 2H, CH-arom POP), 7.32 (m, 2H, CH-arom POP), 7.01 (m, 2H, 1H py + 1H Ph), 6.75 (t, ^3^*J*_H–H_ = 7.1, 1H, Ph), 6.37 (t, ^3^*J*_H–H_ = 7.1, 1H, Ph), 5.70 (d, ^3^*J*_H–H_ = 7.9, 1H, Ph), 2.64 (m, 2H, PC*H*(CH_3_)_2_), 2.01 (s, 3H, CH_3_), 1.69 (m, 2H, PC*H*(CH_3_)_2_), 1.51 (s, 3H, CH_3_), 1.06–0.95 (m, 12H, PCH(C*H*_3_)_2_), 0.91 (dvt, ^3^*J*_H–H_ = 7.6, *N* = 15.9, 6H, PCH(C*H*_3_)_2_), −0.04 (dvt, ^3^*J*_H–H_ = 7.8, *N* = 15.3, 6H, PCH(C*H*_3_)_2_). ^13^C{^1^H}-apt NMR (100.62 MHz, CD_2_Cl_2_, 233 K): δ 158.0 (dt, ^1^*J*_C–Rh_ = 34, ^2^*J*_C–P_ = 8, Rh–C py), 154.3 (vt, *N* = 11, C-arom POP), 142.2 (s, CH py), 139.4 (s, CH Ph), 138.6 (s, CH py), 135.4 (dt, ^1^*J*_C–Rh_ = 44, ^2^*J*_C–P_ = 9, Rh–C Ph), 132.5 (s, CH-arom POP), 132.4 (s, C-arom POP), 132.3 (s, CH Ph), 130.5 (s, CH-arom POP), 127.9 (s, CH Ph), 127.8 (s, CH Ph), 126.8 (s, CH-arom POP), 123.9 (s, CH Ph), 122.5 (s, CH py), 119.5 (s, CH py), 116.7 (vt, *N* = 30, C-arom POP), 36.2 (s, C(*C*H_3_)_2_), 34.8 (s, *C*(CH_3_)_2_), 27.1 (s, C(*C*H_3_)_2_), 25.3 (vt, *N* = 22, P*C*H(CH_3_)_2_), 23.7 (vt, *N* = 26, P*C*H(CH_3_)_2_), 18.4, 16.2, 16.1 (all s, PCH(*C*H_3_)_2_), 16.8 (vt, *N* = 8, PCH(*C*H_3_)_2_). ^31^P{^1^H} NMR (161.98 MHz, CD_2_Cl_2_, 233 K): δ 37.2 (d, ^1^*J*_Rh–P_ = 113). ^19^F{^1^H} NMR (282.38 MHz, CD_2_Cl_2_, 298 K): δ −153.5 (s, BF_4_).

#### Reaction of RhPh_2_Cl{κ^3^-P,O,P-[xant(P^i^Pr_2_)_2_]} (**3**) with AgBF_4_: Preparation of [RhPh_2_{κ^3^-P,O,P-[xant(P^i^Pr_2_)_2_]}]BF_4_ (**7**)

A solution of **3** (100 mg, 0.14 mmol) in acetone (3 mL) was treated with AgBF_4_ (27 mg, 0.14 mmol), and the resulting mixture was stirred at room temperature in the absence of light for 1 h. After this time, it was filtered through Celite to remove the silver salts and was evaporated to dryness to afford a yellow residue. The addition of diethyl ether (4 mL) afforded a yellow solid that was washed with diethyl ether (2 × 2 mL) and dried in vacuo. Yield: 102 mg (95%). Anal. Calcd for C_39_H_50_BF_4_OP_2_Rh: C, 59.56; H, 6.41. Found: C, 59.12; H, 6.43. HRMS (electrospray, *m*/*z*): calcd for C_39_H_50_OP_2_Rh [M]^+^, 699.2386; found, 699.2379. IR (cm^–1^): ν(C–O–C) 1183 (m), ν(B–F) 1053 (vs). ^1^H NMR (300.13 MHz, CD_2_Cl_2_, 298 K): δ 7.97 (dd, ^3^*J*_H–H_ = 7.5, ^3^*J*_H–H_ = 1.3, 2H, CH-arom POP), 7.71–7.47 (m, 4H, CH-arom POP), 7.36 (br, 4H, Ph), 7.01 (m, 6H, Ph), 2.73 (m, 4H, PC*H*(CH_3_)_2_), 1.88 (s, 6H, CH_3_), 0.95 (dvt, ^3^*J*_H–H_ = 7.4, *N* = 16.6, 12H, PCH(C*H*_3_)_2_), 0.81 (dvt, ^3^*J*_H–H_ = 6.7, *N* = 14.2, 12H, PCH(C*H*_3_)_2_). ^13^C{^1^H}-apt NMR (75.48 MHz, CD_2_Cl_2_, 298 K): δ 153.4 (vt, *N* = 9.3, C-arom POP), 141.7 (dt, ^1^*J*_C–Rh_ = 41, ^2^*J*_C–P_ = 8, Rh–C Ph), 133.1 (s, CH-arom POP), 132.8 (s, C-arom POP, inferred from the HMBC spectrum), 132.5 (s, CH Ph), 131.4 (s, CH-arom POP), 127.9 (s, CH Ph), 127.0 (s, CH-arom POP), 124.7 (s, CH Ph), 117.4 (vt, *N* = 28.4, C-arom POP), 34.6 (s, *C*(*C*H_3_)_2_), 32.8 (s, C(*C*H_3_)_2_), 24.8 (vt, *N* = 23, P*C*H(CH_3_)_2_), 18.0, 17.1 (both s, PCH(*C*H_3_)_2_). ^13^C{^1^H}-apt NMR (100.62, CD_2_Cl_2_, 183 K): δ 152.7 (vt, *N* = 9, C-arom POP), 146.6 (broad doublet, ^1^*J*_C–Rh_ = 42, Rh–C Ph), 138.7 (broad doublet, ^1^*J*_C–Rh_ = 44, Rh–C Ph), 138.2 (s, CH Ph), 132.8 (s, CH-arom POP), 131.5 (s, C-arom POP), 131.4 (s, CH-arom POP), 128.5 (s, CH Ph), 128.2 (s, CH Ph), 127.4 (s, CH Ph), 126.8 (s, CH Ph), 126.5 (s, CH-arom POP), 124.2 (s, CH Ph), 123.6 (s, CH Ph), 116.1 (vt, *N* = 29, C-arom POP), 35.3 (s, C(*C*H_3_)_2_), 34.1 (s, *C*(CH_3_)_2_), 29.8 (s, C(*C*H_3_)_2_), 25.2 (vt, *N* = 29, P*C*H(CH_3_)_2_), 22.6 (vt, *N* = 22, P*C*H(CH_3_)_2_), 18.4, 16.4 (both s, PCH(*C*H_3_)_2_). ^31^P{^1^H} NMR (121.50 MHz, CD_2_Cl_2_, 298 K): δ 31.2 (d, ^1^*J*_P–Rh_ = 119). ^19^F{^1^H} NMR (282.38 MHz, CD_2_Cl_2_, 298 K): δ −153.3 (s, BF_4_).

#### Reaction of RhPh(CH_2_Ph)Cl{κ^3^-P,O,P-[xant(P^i^Pr_2_)_2_]} (**4**) with AgBF_4_: Preparation of [RhPh(CH_2_Ph){κ^3^-P,O,P-[xant(P^i^Pr_2_)_2_]}]BF_4_ (**8**)

A solution of **4** (100 mg, 0.13 mmol) in acetone (3 mL) was treated with AgBF_4_ (27 mg, 0.14 mmol), and the resulting mixture was stirred at room temperature in the absence of light for 1 h. After this time, the mixture was filtered through Celite to remove the silver salts and the solution obtained was evaporated to dryness to afford a yellow residue. The addition of diethyl ether (4 mL) afforded a yellow solid that was washed with diethyl ether (2 × 2 mL) and dried in vacuo. Yield: 105 mg (98%). Anal. Calcd for C_40_H_52_BF_4_OP_2_Rh: C, 60.01; H, 6.55. Found: C, 59.64; H, 6.77. HRMS (electrospray, *m*/*z*): calcd for C_40_H_52_OP_2_Rh [M]^+^, 713.2543; found, 713.2555. IR (cm^–1^): ν(C–O–C) 1183 (m), ν(B–F) 1053 (vs). ^1^H NMR (300.13 MHz, CD_2_Cl_2_, 233 K): δ 7.83 (d, ^3^*J*_H–H_ = 7.4, 2H, CH-arom POP), 7.71 (d, ^3^*J*_H–H_ = 7.3, 2H, CH_2_Ph), 7.58–7.19 (m, 8H, 4 CH-arom POP + 1H Ph + 3H CH_2_Ph), 6.95 (t, ^3^*J*_H–H_ = 8.1, 1H, Ph), 6.71 (t, ^3^*J*_H–H_ = 7.1, 1H, Ph), 6.15 (t, ^3^*J*_H–H_ = 7.7, 1H, Ph), 5.50 (d, ^3^*J*_H–H_ = 8.4, 1H, Ph), 4.82 (m, 2H, Rh–C*H*_2_Ph), 2.89 (m, 2H, PC*H*(CH_3_)_2_), 2.66 (m, 2H, PC*H*(CH_3_)_2_), 2.03 (s, 3H, CH_3_), 1.56 (dvt, ^3^*J*_H–H_ = 8.47, *N* = 16.6, 6H, PCH(C*H*_3_)_2_), 1.24 (s, 3H, CH_3_), 0.97 (dvt, ^3^*J*_H–H_ = 5.9, *N* = 11.9, 6H, PCH(C*H*_3_)_2_), 0.31 (dvt, ^3^*J*_H–H_ = 8.0, *N* = 16.4, 6H, PCH(C*H*_3_)_2_), 0.04 (dvt, ^3^*J*_H–H_ = 7.4, *N* = 15.1, 6H, PCH(C*H*_3_)_2_). ^13^C{^1^H}-apt NMR (75.48 MHz, CD_2_Cl_2_, 233 K): δ 155.2 (s, C-arom POP), 141.5 (s, C CH_2_Ph), 134.6 (s, CH Ph), 133.4 (s, C-arom POP), 132.8 (s, CH-arom POP), 131.1 (dt, ^1^*J*_C–Rh_ = 41, ^2^*J*_C–P_ = 8, Rh–C Ph), 130.3 (s, CH CH_2_Ph), 130.2 (s, CH Ph), 129.6 (s, CH CH_2_Ph), 129.1 (s, CH-arom POP), 128.6 (s, CH Ph), 128.3 (s, CH CH_2_Ph), 127.8 (s, CH Ph), 126.7 (s, CH-arom POP), 124.7 (s, CH Ph), 115.1 (vt, *N* = 31, C-arom POP), 35.0 (s, C(*C*H_3_)_2_), 34.6 (s, *C*(CH_3_)_2_), 26.9 (vt, *N* = 20, P*C*H(CH_3_)_2_), 23.5 (vt, *N* = 23, P*C*H(CH_3_)_2_), 23.1 (s, C(*C*H_3_)_2_), 22.6 (d, ^1^*J*_C–Rh_ = 28, Rh–*C*H_2_Ph), 19.1, 17.6, 16.7, 16.1 (all s, PCH(*C*H_3_)_2_). ^31^P{^1^H} NMR (121.50 MHz, CD_2_Cl_2_, 298 K): δ 27.8 (d, ^1^*J*_P–Rh_ = 121). ^19^F{^1^H} NMR (282.38 MHz, CD_2_Cl_2_, 298 K): δ −153.5 (s, BF_4_).

#### Reaction of RhPh(CH_2_Cl)Cl{κ^3^-P,O,P-[xant(P^i^Pr_2_)_2_]} (**5a–5b**) with AgBF_4_

A solution of **5a–5b** (100 mg, 0.14 mmol) in acetone (3 mL) was treated with AgBF_4_ (28 mg, 0.14 mmol), and the resulting mixture was stirred at room temperature in the absence of light for 1 h. After this time, the mixture was filtered through Celite to remove the silver salts and the solution obtained was evaporated to dryness to afford a yellow residue. The addition of diethyl ether (4 mL) afforded a yellow solid. According to the ^1^H and ^31^P{^1^H} NMR spectra, the solid is a mixture from which [RhPh(CH_2_Cl){κ^3^-P,O,P-[xant(P^i^Pr_2_)_2_]}]BF_4_ (**9**) and [Rh(CO){κ^3^-P,O,P-[xant(P^i^Pr_2_)_2_]}]BF_4_ (**10**, vide infra) were identified.

##### Spectroscopic Data of [RhPh(CH_2_Cl){κ^3^-P,O,P-[xant(P^i^Pr_2_)_2_]}]BF_4_ (**9**)

HRMS (electrospray, *m*/*z*): calcd for C_34_H_47_ClOP_2_Rh [M]^+^, 671.1840; found, 671.1868. ^1^H NMR (400.13 MHz, acetone-*d*_6_, 243 K): δ 8.19 (d, ^3^*J*_H–H_ = 7.6, 2H, CH-arom POP), 7.82–7.63 (m, 4H, CH-arom POP), 7.29 (m, 1H, Ph), 6.98 (t, ^3^*J*_H–H_ = 7.5, 1H, Ph), 6.81 (t, ^3^*J*_H–H_ = 7.7, 1H, Ph), 6.41 (t, ^3^*J*_H–H_ = 6.9, 1H, Ph), 5.96 (m, 3H C*H*_2_Cl + 1H Ph), 3.19 (m, 2H, PC*H*(CH_3_)_2_), 2.98 (m, 2H, PC*H*(CH_3_)_2_), 2.12 (s, 3H, CH_3_), 1.58 (s, 3H, CH_3_), 1.47 (dvt, ^3^*J*_H–H_ = 8.7, *N* = 16.3, 6H, PCH(C*H*_3_)_2_), 1.25 (dvt, ^3^*J*_H–H_ = 6.2, *N* = 11.8, 6H, PCH(C*H*_3_)_2_), 0.98 (dvt, ^3^*J*_H–H_ = 7.8, *N* = 15.4, 6H, PCH(C*H*_3_)_2_), 0.03 (dvt, ^3^*J*_H–H_ = 7.6, *N* = 14.4, 6H, PCH(C*H*_3_)_2_). ^13^C{^1^H}-apt NMR (100.62 MHz, acetone-*d*_6_, 253 K): 155.1 (vt, *N* = 11, C-arom POP), 137.1 (s, CH Ph), 135.1 (dt, ^1^*J*_C–Rh_ = 44, ^2^*J*_C–P_ = 9, Rh–C Ph), 134.0 (s, CH-arom POP), 133.4 (vt, *N* = 5, C-arom POP), 132.1 (s, CH-arom POP), 130.7, 129.5, 128.7 (all s, CH Ph), 128.0 (vt, *N* = 6, CH-arom POP), 125.3 (s, CH-arom Ph), 116.5 (vt, *N* = 31, C-arom POP), 45.5 (dt, ^1^*J*_C–Rh_ = 34, ^2^*J*_C–P_ = 7, Rh–*C*H_2_Cl), 36.1 (s, C(*C*H_3_)_2_), 35.3 (s, *C*(CH_3_)_2_), 27.5 (s, C(*C*H_3_)_2_), 26.4 (vt, *N* = 21, P*C*H(CH_3_)_2_), 24.4 (dvt, ^1^*J*_C–Rh_ = 2, *N* = 26, P*C*H(CH_3_)_2_), 20.0, 16.8, 16.6, 16.5 (all s, PCH(*C*H_3_)_2_). ^31^P{^1^H} NMR (161.98 MHz, acetone-*d*_6_, 243 K): δ 31.2 (d, ^1^*J*_Rh–P_ = 113).

#### Preparation of [Rh(CO){κ^3^-P,O,P-[xant(P^i^Pr_2_)_2_]}]BF_4_ (**10**)

A solution of **5a–5b** (94 mg, 0.13 mmol) in acetone (3 mL) was treated with AgBF_4_ (26 mg, 0.13 mmol), and the resulting mixture was stirred at room temperature in the absence of light for 1 h. After this time, the mixture was filtered through Celite to remove the silver salts and the solution obtained was evaporated to dryness to afford a yellow residue. This residue was dissolved in acetone (3 mL), was stirred at 70 °C for 24 h, and was evaporated to dryness, and the addition of diethyl ether (4 mL) afforded a yellow solid that was washed with diethyl ether (2 × 2 mL) and dried in vacuo. Yield: 76 mg (87%). Anal. Calcd C_28_H_40_BF_4_O_2_P_2_Rh: C, 50.93; H, 6.11. Found: C, 51.32; H, 6.32. HRMS (electrospray, *m*/*z*): calcd for C_28_H_40_O_2_P_2_Rh [M]^+^, 573.1553; found, 573.1621. IR (cm^–1^): ν(CO) 1978 (s), ν(C–O–C) 1190 (m), ν(B–F) 1054 (vs). ^1^H NMR (300.13 MHz, acetone-*d*_6_, 273 K): δ 8.07 (dd, ^3^*J*_H–H_ = 7.8, ^4^*J*_H–H_ = 1.4, 2H, CH-arom POP), 7.93 (m, 2H, CH-arom POP), 7.62 (t, ^3^*J*_H–H_ = 7.6, 2H, CH-arom POP), 3.00 (m, 4H, PC*H*(CH_3_)_2_), 1.78 (s, 6H, CH_3_), 1.41 (dvt, ^3^*J*_H–H_ = 11.8, *N* = 17.0, 12H, PCH(C*H*_3_)_2_), 1.22 (dvt, ^3^*J*_H–H_ = 9.9, *N* = 17.0, 12H, PCH(C*H*_3_)_2_). ^13^C{^1^H}-apt NMR (75.48 MHz, acetone-*d*_6_, 273 K): δ 191.5 (dt, ^1^*J*_C–Rh_ = 86, ^2^*J*_C–P_ = 14, Rh–CO), 156.7 (vt, *N* = 16, C-arom POP), 133.5 (s, CH-arom POP), 133.4 (s, CH-arom POP), 132.5 (vt, *N* = 6, C-arom POP), 128.2 (vt, *N* = 6, CH-arom POP), 118.4 (dvt, ^2^*J*_C–Rh_ = 1, *N* = 29, C-arom POP), 34.8 (vt, *N* = 1, *C*(CH_3_)_2_), 33.4 (s, C(*C*H_3_)_2_), 27.4 (dvt, ^1^*J*_C–Rh_ = 2, *N* = 14, P*C*H(CH_3_)_2_), 19.8 (vt, *N* = 6.0, PCH(*C*H_3_)_2_), 19.2 (s, PCH(*C*H_3_)_2_). ^31^P{^1^H} NMR (121.50 MHz, acetone-*d*_6_, 273 K): δ 64.0 (d, ^1^*J*_Rh–P_ = 114).

#### Reaction of [RhPh_2_{κ^3^-P,O,P-[xant(P^i^Pr_2_)_2_]}]BF_4_ (**7**) with Fluorobenzene

An NMR tube was charged with a solution of **7** (5 mg, 6.3 × 10^–3^ mmol) in fluorobenzene (2 mL) and it is introduced in an oil bath preheated at 80 °C, and it was periodically checked by ^31^P{^1^H} NMR spectroscopy. After 5 days, the ^31^P{^1^H} NMR spectrum showed quantitative conversion to RhH(*o*-C_6_H_4_F)(κ^1^-FBF_3_){κ^3^-P,O,P-[xant(P^i^Pr_2_)_2_]} (**11a**) and RhH(*m*-C_6_H_4_F)(κ^1^-FBF_3_){κ^3^-P,O,P-[xant(P^i^Pr_2_)_2_]} (**11b**), while the GC–MS spectrum showed the formation of biphenyl.

#### Reaction of [RhPh(CH_2_Ph){κ^3^-P,O,P-[xant(P^i^Pr_2_)_2_]}]BF_4_ (**8**) with Fluorobenzene

An NMR tube was charged with a solution of **8** (5 mg, 6.2 × 10^–3^ mmol) in fluorobenzene (2 mL) and it is introduced in an oil bath preheated at 80 °C. ^31^P{^1^H} NMR spectra were recorded periodically and after 2 days showed quantitative conversion to RhH(*o*-C_6_H_4_F)(κ^1^-FBF_3_){κ^3^-P,O,P-[xant(P^i^Pr_2_)_2_]} (**11a**) and RhH(*m*-C_6_H_4_F)(κ^1^-FBF_3_){κ^3^-P,O,P-[xant(P^i^Pr_2_)_2_]} (**11b**), while in the ^1^H NMR spectrum, a singlet at 4.00 ppm, assigned to benzylbenzene,^38^ is observed.

#### Kinetic Analysis of the Reaction of **7** with Fluorobenzene

In the glovebox, an NMR tube was charged with a solution of **7** (5 mg, 6.3 × 10^–3^ mmol) in fluorobenzene (2 mL), and a capillary tube filled with a solution of the internal standard (PCy_3_) in toluene-*d*_8_ was placed in the NMR tube. The tube was introduced into a thermostatic bath at 343, 348, 353, 358, or 363 K and the reaction was monitored by ^31^P{^1^H} NMR spectroscopy (a delay of 25 s was used) at different intervals of time. The experiments were performed in duplicate. Rate constants were obtained by plotting eq 5. Errors were calculated using the standard deviation data provided by Microsoft Excel.

#### Kinetic Analysis of the Reaction of **8** with Fluorobenzene

In the glovebox, an NMR tube was charged with a solution of **8** (5 mg, 6.2 × 10^–3^ mmol) in fluorobenzene (2 mL), and a capillary tube filled with a solution of the internal standard (PCy_3_) in toluene-*d*_8_ was placed in the NMR tube. The tube was introduced into a thermostatic bath at 338, 353, 358, or 363 K and the reaction was monitored by ^31^P{^1^H} NMR spectroscopy (a delay of 25 s was used) at different intervals of time. The experiments were performed in duplicate. Rate constants were obtained by plotting eq 5. Errors were calculated using the standard deviation data provided by Microsoft Excel.

#### Reaction of RhCl{κ^3^-P,O,P-[xant(P^i^Pr_2_)_2_]} (**12**) with AgBF_4_ in Fluorobenzene: Preparation of RhH(*o*-C_6_H_4_F)(κ^1^-FBF_3_){κ^3^-P,O,P-[xant(P^i^Pr_2_)_2_]} (**11a**) and RhH(*m*-C_6_H_4_F)(κ^1^-FBF_3_){κ^3^-P,O,P-[xant(P^i^Pr_2_)_2_]} (**11b**)

A solution of **12** (100 mg, 0.17 mmol) in fluorobenzene (3 mL) was treated with AgBF_4_ (34 mg, 0.17 mmol), and the resulting mixture was stirred at room temperature in the absence of light for 1 h. After this time, the mixture was filtered through Celite to remove the silver salts and the solution obtained was evaporated to dryness to afford a light yellow residue. The addition of diethyl ether (4 mL) afforded a yellow solid that was washed with diethyl ether (2 × 2 mL) and dried in vacuo. Yield: 67 mg (53%). The ^31^P{^1^H} NMR spectra in acetone-*d*_6_ show the formation of an isomeric mixture of RhH(*o*-C_6_H_4_F)(κ^1^-FBF_3_){κ^3^-P,O,P-[xant(P^i^Pr_2_)_2_]} (**11a**) and RhH(*m*-C_6_H_4_F)(κ^1^-FBF_3_){κ^3^-P,O,P-[xant(P^i^Pr_2_)_2_]} (**11b**) in a ratio 70:30. Anal. Calcd for C_33_H_45_BF_5_OP_2_Rh: C, 54.41; H, 6.23. Found: C, 54.39; H, 6.25. HRMS (electrospray, *m*/*z*): calcd for C_33_H_45_FOP_2_Rh [M]^+^, 641.1979; found, 641.1986. IR (cm^–1^): ν(C–O–C) 1188 (m), ν(B–F) 1095 (s), 953 (s), 745 (s).

##### NMR Data for RhH(o-C_6_H_4_F)(κ^1^-FBF_3_){κ^3^-P,O,P-[xant(P^i^Pr_2_)_2_]} (**11a**)

^1^H NMR (400.13 MHz, acetone-*d*_6_, 273 K): δ 8.08 (br, 1H, C_6_H_4_-2-F), 8.01 (dd, ^3^*J*_H–H_ = 7.8, ^4^*J*_H–H_ = 1.3, 2H, CH-arom POP), 7.80 (m, 2H, CH-arom POP), 7.50 (t, ^3^*J*_H–H_ = 7.6, 2H, CH-arom POP), 6.96 (m, 2H, C_6_H_4_-2-F), 6.83 (t, ^3^*J*_H–H_ = 8.6, 1H, C_6_H_4_-2-F), 3.00 (m, 2H, PC*H*(CH_3_)_2_), 2.68 (m, 2H, PC*H*(CH_3_)_2_), 1.77 (s, 3H, CH_3_), 1.76 (s, 3H, CH_3_), 1.17 (dvt, ^3^*J*_H–H_ = 7.5, *N* = 14.4, 6H, PCH(C*H*_3_)_2_), 1.08 (dvt, ^3^*J*_H–H_ = 7.2, *N* = 14.4, 6H, PCH(C*H*_3_)_2_), 1.01 (dvt, ^3^*J*_H–H_ = 9.3, *N* = 16.5, 6H, PCH(C*H*_3_)_2_), 0.86 (dvt, ^3^*J*_H–H_ = 9.5, *N* = 16.5, 6H, PCH(C*H*_3_)_2_), −18.95 (dt, ^1^*J*_H–Rh_ = 30.6, ^2^*J*_H–H_ = 12.9, 1H, Rh–H). ^13^C{^1^H}-apt NMR (100.63 MHz, acetone-*d*_6_, 273 K): δ 166.2 (broad d, ^1^*J*_C–F_ = 230, C–F C_6_H_4_F), 154.3 (vt, *N* = 13, C-arom POP), 145.7 (broad d, ^1^*J*_C–Rh_ = 34, Rh–C C_6_H_4_F), 136.3 (broad d, *J*_C–F_ = 10, CH C_6_H_4_F), 132.8 (s, CH-arom POP), 132.6 (s, CH-arom POP), 132.3 (dvt, *J*_C–Rh_ = 3, *N* = 20, C-arom POP), 127.2 (vt, *N* = 6, CH arom POP), 124.9 (d, *J*_C–F_ = 8, CH C_6_H_4_F), 123.6 (s, CH C_6_H_4_F), 120.4 (vt, *N* = 28, C-arom POP), 114.3 (d, *J*_C–F_ = 30, CH, C_6_H_4_F), 34.9 (s, *C*(CH_3_)_2_), 34.4, 33.2 (both s, C(*C*H_3_)_2_), 27.9 (vt, *N* = 29, P*C*H(CH_3_)_2_), 26.2 (vt, *N* = 23, P*C*H(CH_3_)_2_), 19.0, 17.6 (both s, PCH(*C*H_3_)_2_), 17.5 (vt, *N* = 5, PCH(*C*H_3_)_2_). ^31^P{^1^H} NMR (121.4 MHz, acetone-*d*_6_, 298 K): δ 43.6 (d, ^1^*J*_Rh–P_ = 111.0). ^19^F{^1^H} NMR (376.46 MHz, acetone-*d*_6_, 273 K): δ −88.3 (d, *J*_F–Rh_ = 21.7, C_6_H_4_F), −151.4 (s, BF_4_).

##### Characteristic NMR Data for RhH(*m*-C_6_H_4_F)(κ^1^-FBF_3_){κ^3^-P,O,P-[xant(P^i^Pr_2_)_2_]} (**11b**)

^1^H NMR (400.13 MHz, acetone-*d*_6_, 273 K): δ −19.92 (dt, ^1^*J*_H–Rh_ = 35.5, ^2^*J*_H–P_ = 13.4, 1H, Rh–H). ^31^P{^1^H} NMR (121.4 MHz, acetone-*d*_6_, 298 K): δ 40.9 (d, ^1^*J*_Rh–P_ = 115). ^19^F{^1^H} NMR (376.46 MHz, acetone-*d*_6_, 273 K): δ −115.7 (s, C_6_H_4_F), −151.4 (s, BF_4_).

#### Reaction of RhH(*o*-C_6_H_4_F)(κ^1^-FBF_3_){κ^3^-P,O,P-[xant(P^i^Pr_2_)_2_]} (**11a**) and RhH(*m*-C_6_H_4_F)(κ^1^-FBF_3_){κ^3^-P,O,P-[xant(P^i^Pr_2_)_2_]} (**11b**) with 2-Butyne: Preparation of [Rh(η^2^-MeC≡CMe){κ^3^-P,O,P-[xant(P^i^Pr_2_)_2_]}]BF_4_ (**13**)

A solution of **11a–11b** (80 mg, 0.11 mmol) in fluorobenzene (3 mL) was treated with 2-butyne (9 μL, 0.11 mmol) and the resulting mixture was stirred at room temperature for 24 h. After this time, the solution was evaporated to dryness to afford a yellow residue. The addition of diethyl ether (4 mL) afforded a yellow solid that was washed with diethyl ether (2 × 2 mL) and dried in vacuo. Yield: 75 mg (98%). Anal. Calcd for C_31_H_46_BF_4_OP_2_Rh: C, 54.25; H, 6.76. Found: C, 54.17; H, 6.89. HRMS (electrospray, *m*/*z*): calcd for C_31_H_46_OP_2_Rh [M]^+^, 599.2073; found, 599.2048. IR (cm^–1^): ν(C≡C) 1994 (w), ν(C–O–C) 1187 (m), ν(B–F) 1051 (vs). ^1^H NMR (300.13 MHz, acetone-*d*_6_, 298 K): δ 7.96 (dd, ^2^*J*_H–H_ = 7.7, ^3^*J*_H–H_ = 1.4, 2H, CH-arom POP), 7.67 (m, 2H, CH-arom POP), 7.51 (t, ^3^*J*_H–H_ = 15.2, 2H, CH-arom POP), 2.70 (m, 4H, PC*H*(CH_3_)_2_), 2.34 (d, ^3^*J*_H–Rh_ = 1.9, 6H, =CC*H*_3_), 1.77 (s, 6H, CH_3_), 1.33 (dvt, ^3^*J*_H–H_ = 9.4, *N* = 16.8, 12H, PCH(C*H*_3_)_2_), 1.25 (dvt, ^3^*J*_H–H_ = 7.5, *N* = 14.7, 12H, PCH(C*H*_3_)_2_). ^13^C{^1^H}-apt NMR (75.48 MHz, acetone-*d*_6_, 298 K): δ 156.9 (vt, *N* = 14, C-arom POP), 133.0 (s, CH-arom POP), 132.8 (s, CH-arom POP), 132.0 (vt, *N* = 5, C-arom POP), 127.4 (vt, *N* = 5, CH-arom POP), 119.1 (vt, *N* = 24, C-arom POP), 56.3 (dt, ^1^*J*_C–Rh_ = 16, ^2^*J*_C–P_ = 3, ≡*C*CH_3_), 34.8 (s, *C*(CH_3_)_2_), 33.9 (s, C(*C*H_3_)_2_), 25.0 (vt, *N* = 22, P*C*H(CH_3_)_2_), 18.4 (vt, *N* = 6, PCH(*C*H_3_)_2_), 10.1 (d, ^2^*J*_C–Rh_ = 1, ≡C*C*H_3_). ^31^P{^1^H} NMR (121.49 MHz, acetone-*d*_6_, 298 K): δ 35.4 (d, ^1^*J*_Rh–P_ = 125). ^19^F{^1^H} NMR (282.38 MHz, acetone-*d*_6_, 298 K): δ −151.8 (s, BF_4_).

#### Reaction of RhH(*o*-C_6_H_4_F)(κ^1^-FBF_3_){κ^3^-P,O,P-[xant(P^i^Pr_2_)_2_]} (**11a**) and RhH(*m*-C_6_H_4_F)(κ^1^-FBF_3_){κ^3^-P,O,P-[xant(P^i^Pr_2_)_2_]} (**11b**) with 1-Phenyl-1-propyne: Preparation of [Rh(η^2^-PhC≡CMe){κ^3^-P,O,P-[xant(P^i^Pr_2_)_2_]}]BF_4_ (**14**)

A solution of **11a–11b** (80 mg, 0.11 mmol) in fluorobenzene (3 mL) was treated with 1-phenyl-1-propyne (14 μL, 0.11 mmol), and the resulting mixture was stirred at room temperature for 24 h. After this time, the solution was evaporated to dryness to afford a yellow residue. The addition of diethyl ether (4 mL) afforded a yellow solid that was washed with diethyl ether (2 × 2 mL) and dried in vacuo. Yield: 77 mg (94%). Anal. Calcd for C_36_H_48_BF_4_OP_2_Rh: C, 57.77; H, 6.46. Found: C, 57.70; H, 6.27. HRMS (electrospray, *m*/*z*): calcd for C_36_H_48_OP_2_Rh [M]^+^, 661.2230; found, 661.2237. IR (cm^–1^): ν(C–O–C) 1186 (m), ν(B–F) 1051–1027 (vs). ^1^H NMR (300.13 MHz, acetone-*d*_6_, 298 K): δ 8.09 (dd, ^2^*J*_H–H_ = 8.0, ^3^*J*_H–H_ = 1.6, 2H, Ph), 8.01 (dd, ^2^*J*_H–H_ = 7.7, ^3^*J*_H–H_ = 1.3, 2H, CH-arom POP), 7.66 (m, 2H, CH-arom POP), 7.53 (t, ^3^*J*_H–H_ = 15.2, 2H, CH-arom POP), 7.50–7.38 (m, 3H, Ph), 2.80–2.60 (m, 5H, 3H ≡CC*H*_3_, 2H PC*H*(CH_3_)_2_), 2.48 (m, 2H, PC*H*(CH_3_)_2_), 1.81 (s, 3H, CH_3_), 1.80 (s, 3H, CH_3_), 1.37 (dvt, ^3^*J*_H–H_ = 9.2, *N* = 17.0, 6H, PCH(C*H*_3_)_2_), 1.26 (dvt, ^3^*J*_H–H_ = 6.9, *N* = 13.8, 6H, PCH(C*H*_3_)_2_), 1.06 (dvt, ^3^*J*_H–H_ = 8.3, *N* = 15.7, 6H, PCH(C*H*_3_)_2_), 1.02 (dvt, ^3^*J*_H–H_ = 9.4, *N* = 16.6, 6H, PCH(C*H*_3_)_2_). ^13^C{^1^H}-apt NMR (75.48 MHz, acetone-*d*_6_, 298 K): δ 156.8 (vt, *N* = 13, C-arom POP), 133.0 (s, CH-arom POP), 132.9 (s, CH-arom POP), 132.3 (d, *J*_Rh–C_ = 2, CH Ph), 132.1 (vt, *N* = 5, C-arom POP), 129.3 (s, CH Ph), 128.8 (s, CH Ph), 127.6 (vt, *N* = 5, CH-arom POP), 126.9 (s, C Ph), 119.0 (vt, *N* = 25, C-arom POP), 72.8 (dt, ^1^*J*_C–Rh_ = 16, ^2^*J*_C–P_ = 5, ≡*C*CH_3_), 61.2 (dt, ^1^*J*_C–Rh_ = 18, ^2^*J*_C–P_ = 3, Ph*C*≡), 34.9 (s, *C*(CH_3_)_2_), 34.1 (s, C(*C*H_3_)_2_), 33.7 (s, C(*C*H_3_)_2_), 25.5 (vt, *N* = 21, P*C*H(CH_3_)_2_), 24.4 (vt, *N* = 23, P*C*H(CH_3_)_2_), 19.0 (vt, *N* = 6, PCH(*C*H_3_)_2_), 18.1, 17.5 (both s, PCH(*C*H_3_)_2_), 17.7 (vt, *N* = 6, PCH(*C*H_3_)_2_), 11.5 (s, ≡C*C*H_3_). ^31^P{^1^H} NMR (121.49 MHz, acetone-*d*_6_, 298 K): δ 35.6 (d, ^1^*J*_Rh–P_ = 122). ^19^F{^1^H} NMR (282.38 MHz, acetone-*d*_6_, 298 K): δ −151.8 (s, BF_4_).

#### Reaction of the Isomeric Mixture of **11a** and **11b** with K^*t*^OBu

A solution of the isomeric mixture of **11a** and **11b** (32 mg, 0.044 mmol) in acetone was treated with KO^*t*^Bu (5 mg, 0.044 mmol), and the resulting mixture was stirred at room temperature for 1 h. After this time, the solution was evaporated to dryness, toluene was added, and the resulting suspension was filtered through Celite to remove the potassium salts. The solution obtained was evaporated to dryness to afford a red residue. ^31^P{^1^H} and ^19^F{^1^H} NMR spectroscopies show the quantitative formation of the previously reported Rh(*o*-C_6_H_4_F){κ^3^-P,O,P-[xant(P^i^Pr_2_)_2_]} (**15a**)^10g^ and Rh(*m*-C_6_H_4_F){κ^3^-P,O,P-[xant(P^i^Pr_2_)_2_]} (**15b**)^10i^ a ratio 7:3. ^31^P{^1^H} NMR (121.49 MHz, benzene-*d*_6_, 298 K): δ 39.7 (d, ^1^*J*_Rh–P_ = 168, **15a**), 37.1 (d, ^1^*J*_Rh–P_ = 174, **15b**). ^19^F{^1^H} NMR (282.38 MHz, benzene-*d*_6_, 298 K): δ −85.4 (dt, ^3^*J*_Rh–F_ = 19.8, ^4^*J*_P–F_ = 4, **15a**), −118.4 (s, **15b**).

#### Protonation of the Isomeric Mixture of **15a** and **15b** with HBF_4_

A solution of the isomeric mixture of **15a** and **15b** (200 mg, 0.31 mmol) in fluorobenzene (3 mL) was treated with HBF_4_·OEt_2_ (43 μL, 0.31 mmol), and the solution was stirred at room temperature for 1 h. After this time, it was evaporated to dryness to afford a light yellow residue. The addition of diethyl ether (4 mL) afforded a white solid that was washed with diethyl ether (2 × 2 mL) and dried in vacuo. Yield: 189 mg (83%). The ^31^P{^1^H} NMR spectrum in acetone-*d*_6_ showed the regeneration of the isomeric mixture of **11a** and **11b**.

## Supporting Information Available

General information for the experimental section, kinetic plots, structural analysis, and NMR spectra (PDF)

## Accession Codes

CCDC 2121578–2121584 contain the supplementary crystallographic data for this paper. These data can be obtained free of charge via www.ccdc.cam.ac.uk/data_request/cif, or by emailing data_request@ccdc.cam.ac.uk, or by contacting The Cambridge Crystallographic Data Centre, 12 Union Road, Cambridge CB2 1EZ, UK; fax: +44 1223 336033.
