# Supplementary material for: C–Cl Oxidative Addition and C–C Reductive Elimination Reactions in the Context of the Rhodium-Promoted Direct Arylation
Source: Organometallics. 2022 Mar 17;41(6):716–32. doi: 10.1021/acs.organomet.1c00643 (PMC8966374; doi:10.1021/acs.organomet.1c00643)
Supplement: Supplementary file 1 — om1c00643_si_001.pdf [file om1c00643_si_001.pdf]

SUPPORTING INFORMATION

C-Cl Oxidative Addition and C-C Reductive Elimination Reactions in the Context of the Rhodium-Promoted Direct Arylation

Laura A. de las Heras, Miguel A. Esteruelas,* Montserrat Oliván, and Enrique Oñate

Departamento de Química Inorgánica – Instituto de Síntesis Química y Catálisis Homogénea (ISQCH) – Centro de Innovación en Química Avanzada (ORFEO-CINQA), Universidad de Zaragoza – CSIC, 50009 Zaragoza, Spain

* e-mail: maester@unizar.es

Contents:

- General Information.	S2
- Kinetic Plots	S3
- Structural Analysis of Complexes **2**, **3**, **4**, **5b**, **6**, **8** and **11a**	S14
- NMR spectra	S17

• General Information

All reactions were carried out with exclusion of air using Schlenk-tube techniques or in a drybox. Pentane, diethyl ether and dichloromethane were obtained oxygen- and water-free from an MBraun solvent purification apparatus, while 2-chloropyridine, chlorobenzene, benzyl chloride, acetone and fluorobenzene were dried and distilled under argon prior to use. ^{1}H, $^{13}C\{^{1}H\}$, $^{31}P\{^{1}H\}$, and ^{19}F NMR spectra were recorded on Bruker 300 ARX, Bruker Avance 300 MHz or Bruker Avance 400 MHz instruments. Chemical shifts (expressed in ppm) are referenced to residual solvent peaks (^{1}H, $^{13}C\{^{1}H\}$), external 85% H_3PO_4 ($^{31}P\{^{1}H\}$), or $CFCl_3$ (^{19}F). Coupling constant J and N ($N = J_{P\text{-}H} + J_{P'\text{-}H}$ for ^{1}H and $N = J_{P\text{-}C} + J_{P'\text{-}C}$ for $^{13}C\{^{1}H\}$) are given in hertz. Attenuated total reflection infrared spectra (ATR-IR) of solid samples were run on a PerkinElmer Spectrum 100 FT-IR spectrometer. C, H, and N analyses were carried out in a PerkinElmer 2400 CHNS/O analyzer. High-resolution electrospray mass spectra were acquired using a MicroTOF-Q hybrid quadrupole time-of-flight spectrometer (Bruker Daltonics, Bremen, Germany). RhPh{κ^3-P,O,P-[xant(P^iPr_2)$_2$]} (**1**)[1] and RhCl{κ^3-P,O,P-[xant(P^iPr_2)$_2$]} (**12**)[2] were prepared by the published methods.

• **Kinetic Plots**

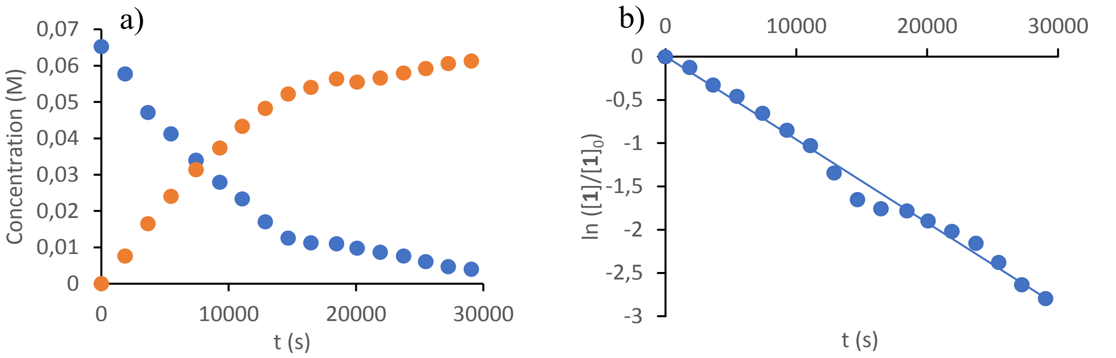

Figure S1. (a) Concentration *versus* time plot for the reaction of **1** (blue circles) with 2-chloropyridine to give **2** (orange circles) at 323 K. (b) Linearized fit for the disappearance of **1**.

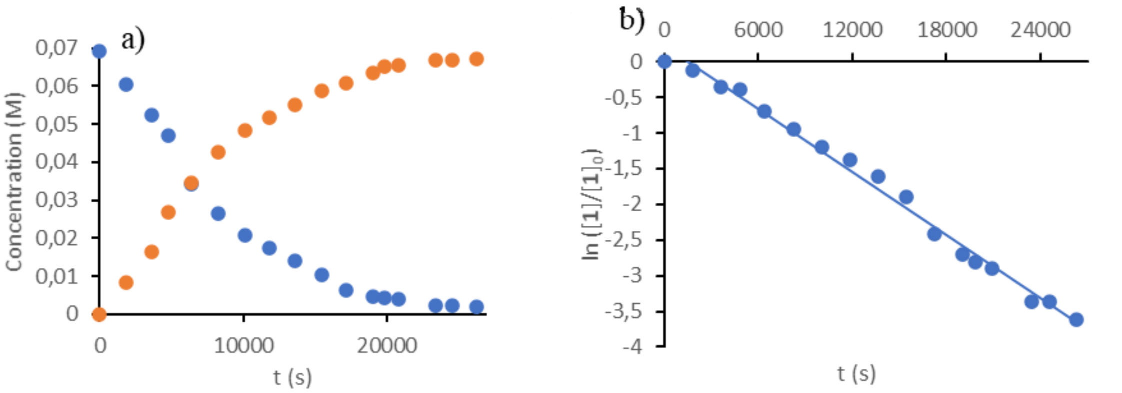

Figure S2. (a) Concentration *versus* time plot for the reaction of **1** (blue circles) with 2-chloropyridine to give **2** (orange circles) at 328 K. (b) Linearized fit for the disappearance of **1**.

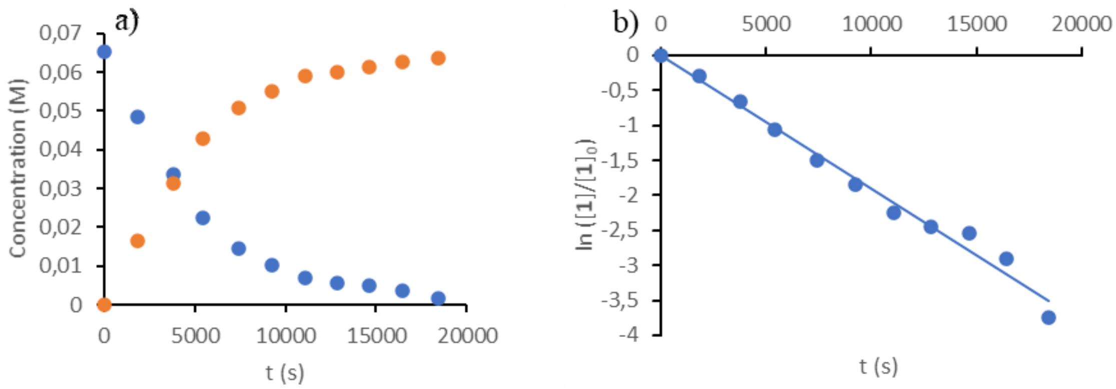

Figure S3. (a) Concentration *versus* time plot for the reaction of **1** (blue circles) with 2-chloropyridine to give **2** (orange circles) at 333 K. (b) Linearized fit for the disappearance of **1**.

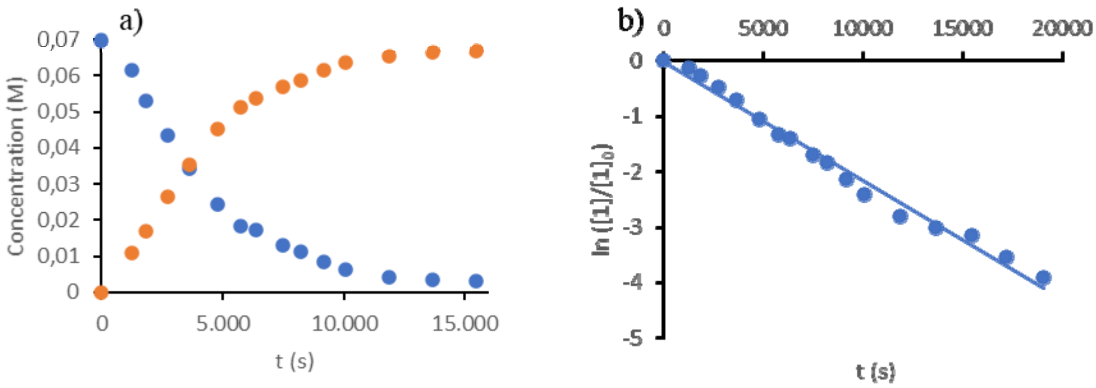

Figure S4. (a) Concentration *versus* time plot for the reaction of **1** (blue circles) with 2-chloropyridine to give **2** (orange circles) at 338 K. (b) Linearized fit for the disappearance of **1**.

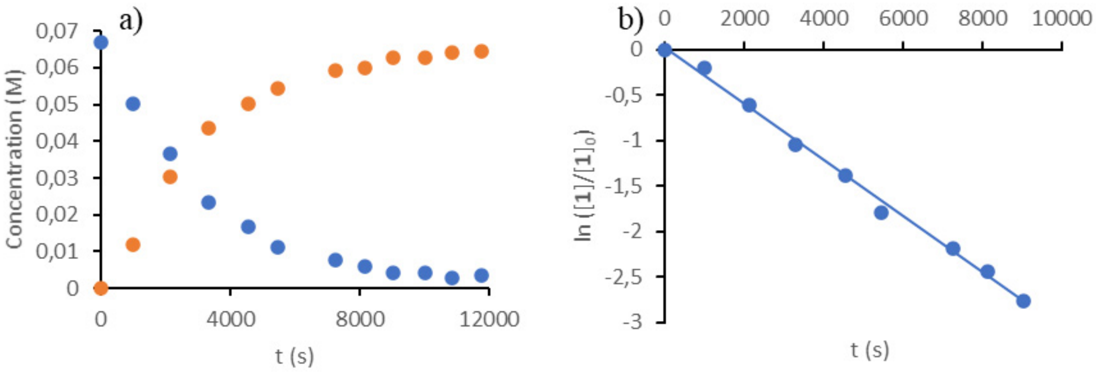

Figure S5. (a) Concentration *versus* time plot for the reaction of **1** (blue circles) with 2-chloropyridine to give **2** (orange circles) at 343 K. (b) Linearized fit for the disappearance of **1**.

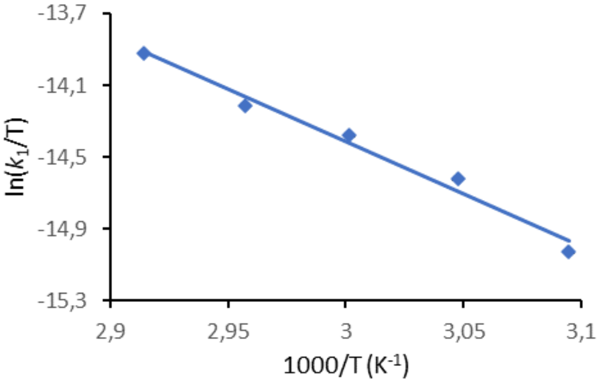

Figure S6. Eyring plot for the formation of **2**.

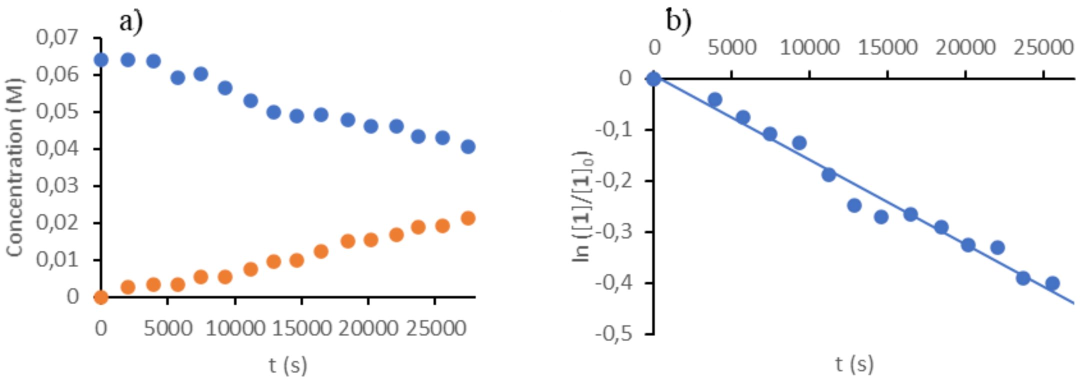

Figure S7. (a) Concentration *versus* time plot for the reaction of **1** (blue circles) with chlorobenzene to give **3** (orange circles) at 363 K. (b) Linearized fit for the disappearance of **1**.

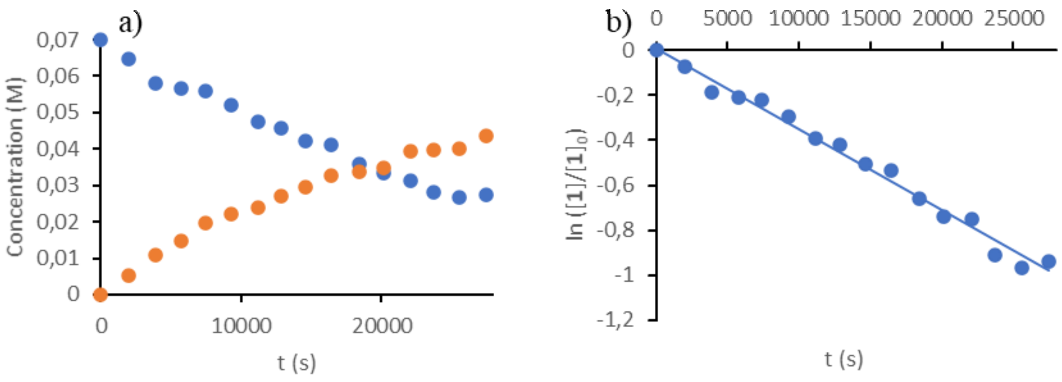

Figure S8. (a) Concentration *versus* time plot for the reaction of **1** (blue circles) with chlorobenzene to give **3** (orange circles) at 373 K. (b) Linearized fit for the disappearance of **1**.

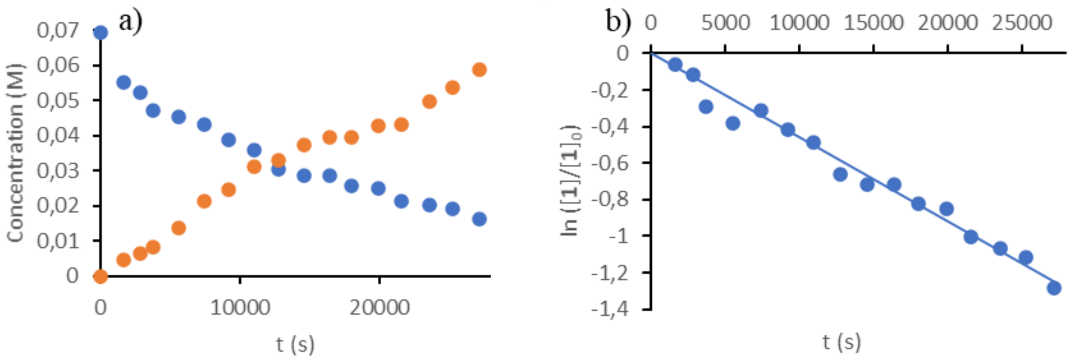

Figure S9. (a) Concentration *versus* time plot for the reaction of **1** (blue circles) with chlorobenzene to give **3** (orange circles) at 383 K. (b) Linearized fit for the disappearance of **1**.

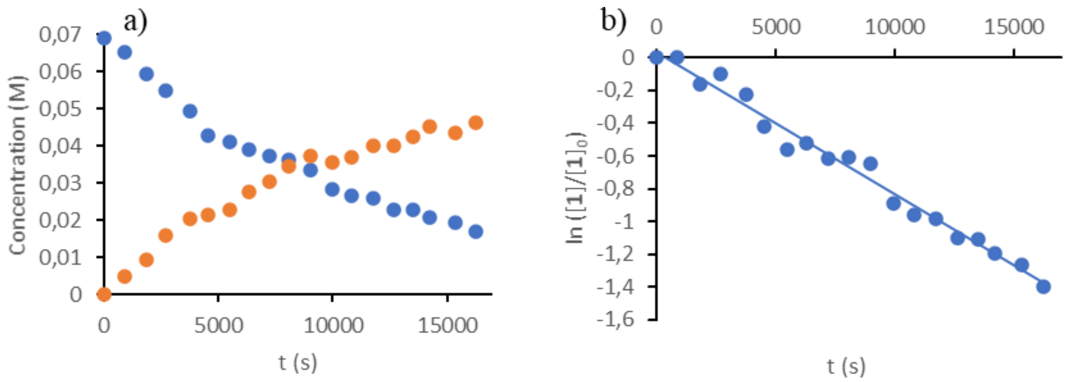

Figure S10. (a) Concentration *versus* time plot for the reaction of **1** (blue circles) with chlorobenzene to give **3** (orange circles) at 393 K. (b) Linearized fit for the disappearance of **1**.

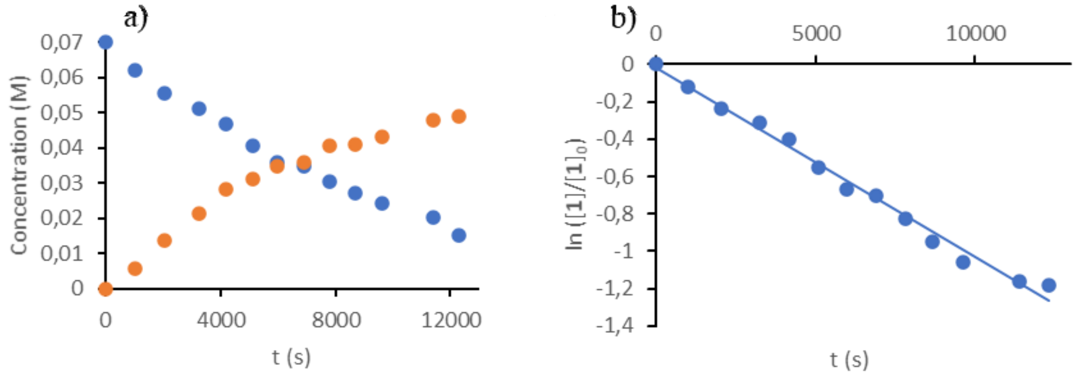

Figure S11. (a) Concentration *versus* time plot for the reaction of **1** (blue circles) with chlorobenzene to give **3** (orange circles) at 398 K. (b) Linearized fit for the disappearance of **1**.

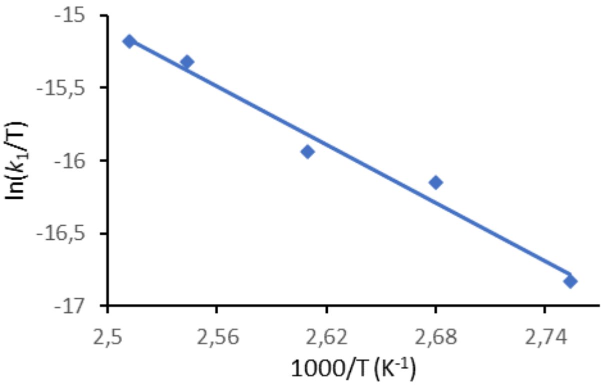

Figure S12. Eyring plot for the formation of **3**.

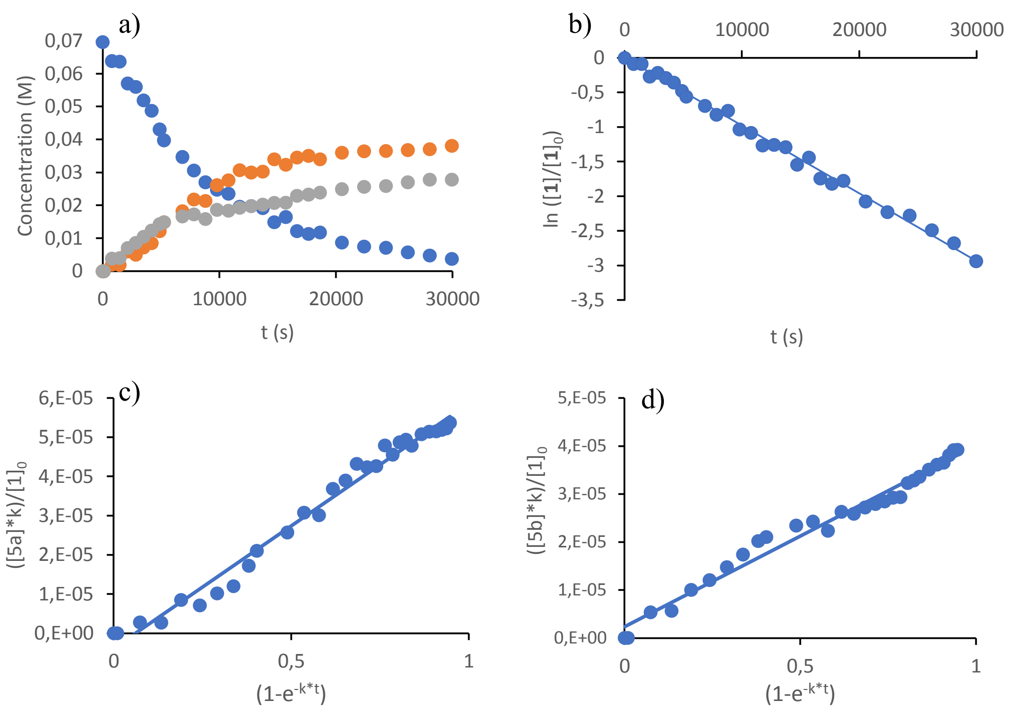

Figure S13. (a) Concentration *versus* time plot for the reaction of **1** (blue circles) with dichlorometane to give **5a** (orange circles) and **5b** (grey circles) at 268 K. (b) Linearized fit for the disappearance of **1**. (c) Linearized fit for the formation of **5a**. (d) Linearized fit for the formation of **5b**.

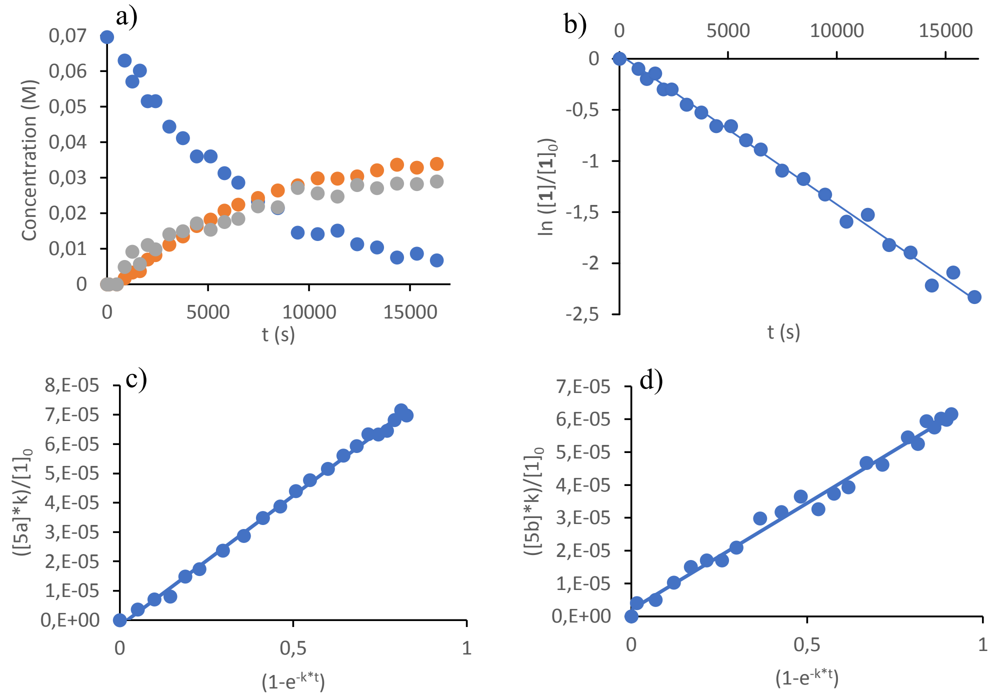

Figure S14. (a) Concentration *versus* time plot for the reaction of **1** (blue circles) with dichlorometane to give **5a** (orange circles) and **5b** (grey circles) at 273 K. (b) Linearized fit for the disappearance of **1**. (c) Linearized fit for the formation of **5a**. (d) Linearized fit for the formation of **5b**.

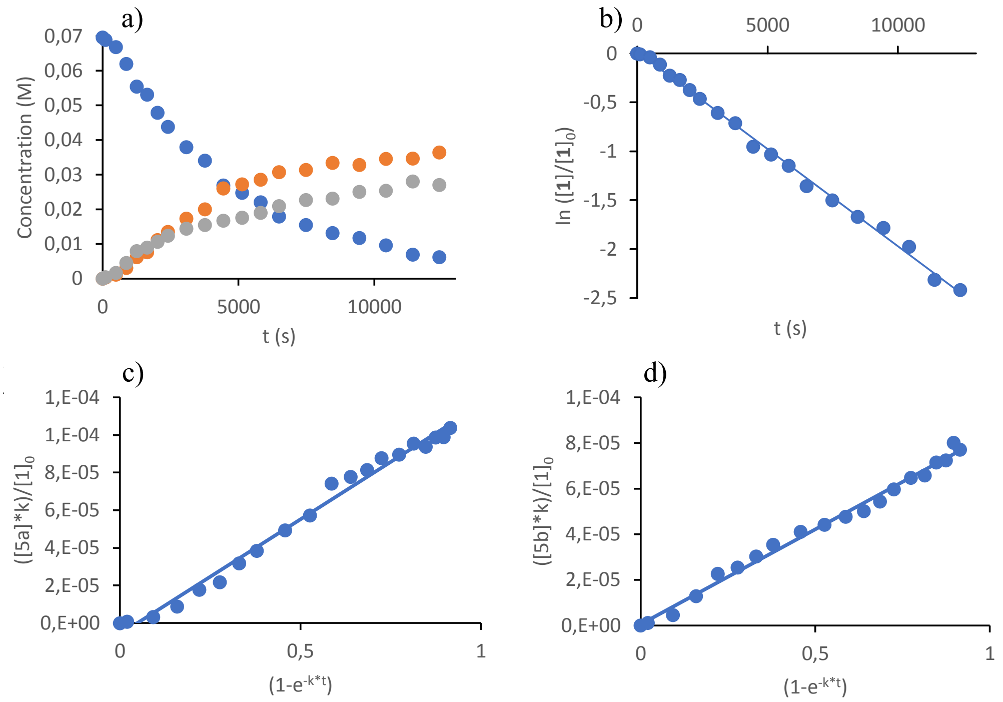

Figure S15. (a) Concentration *versus* time plot for the reaction of **1** (blue circles) with dichlorometane to give **5a** (orange circles) and **5b** (grey circles) at 278 K. (b) Linearized fit for the disappearance of **1**. (c) Linearized fit for the formation of **5a**. (d) Linearized fit for the formation of **5b**.

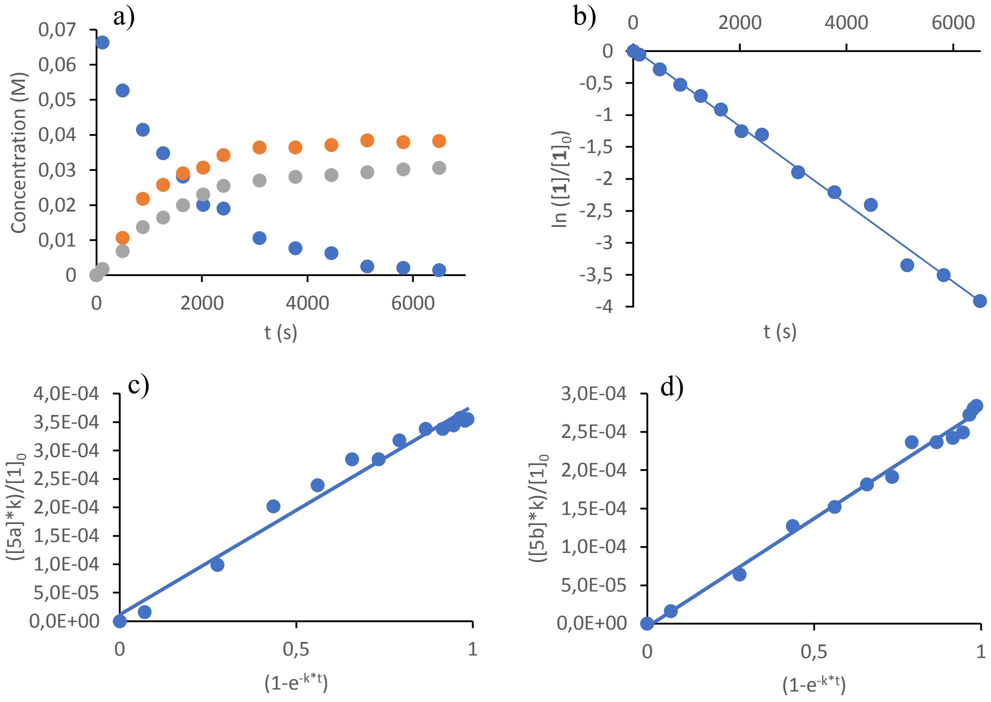

Figure S16. (a) Concentration *versus* time plot for the reaction of **1** (blue circles) with dichlorometane to give **5a** (orange circles) and **5b** (grey circles) at 288 K. (b) Linearized fit for the disappearance of **1**. (c) Linearized fit for the formation of **5a**. (d) Linearized fit for the formation of **5b**.

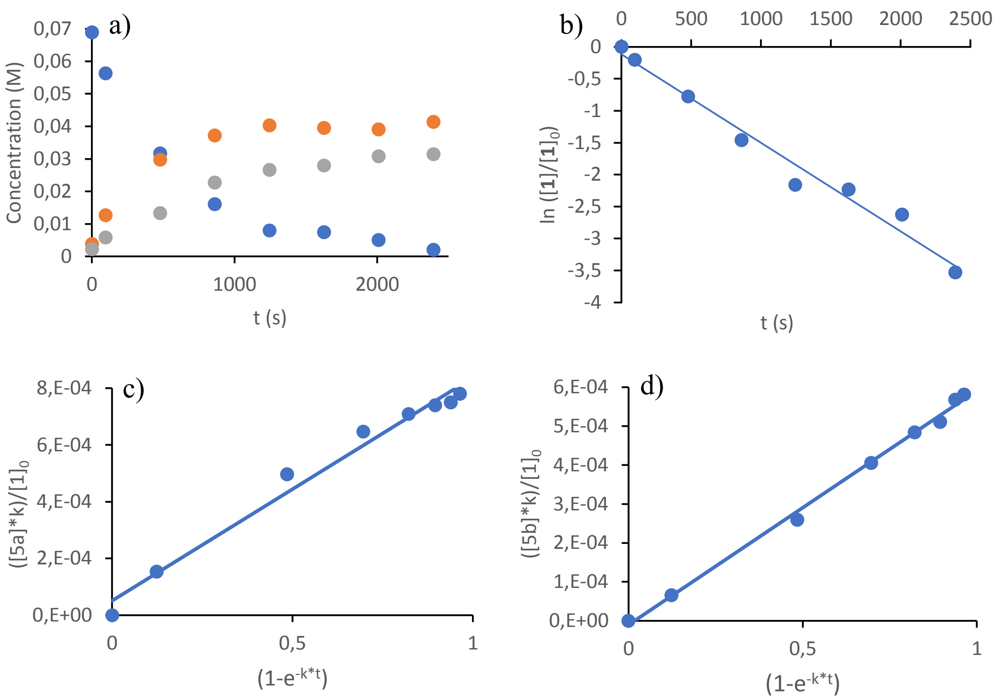

Figure S17. (a) Concentration *versus* time plot for the reaction of **1** (blue circles) with dichloromethane to give **5a** (orange circles) and **5b** (grey circles) at 298 K. (b) Linearized fit for the disappearance of **1**. (c) Linearized fit for the formation of **5a**. (d) Linearized fit for the formation of **5b**.

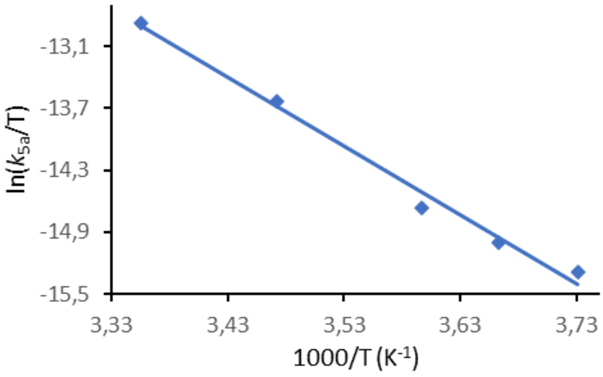

Figure S18. Eyring plot for the formation of **5a**.

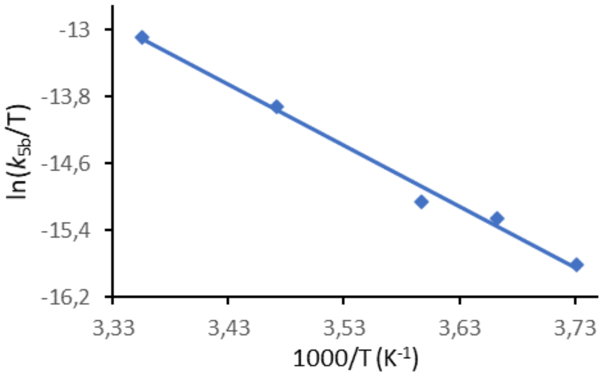

Figure S19. Eyring plot for the formation of **5b**.

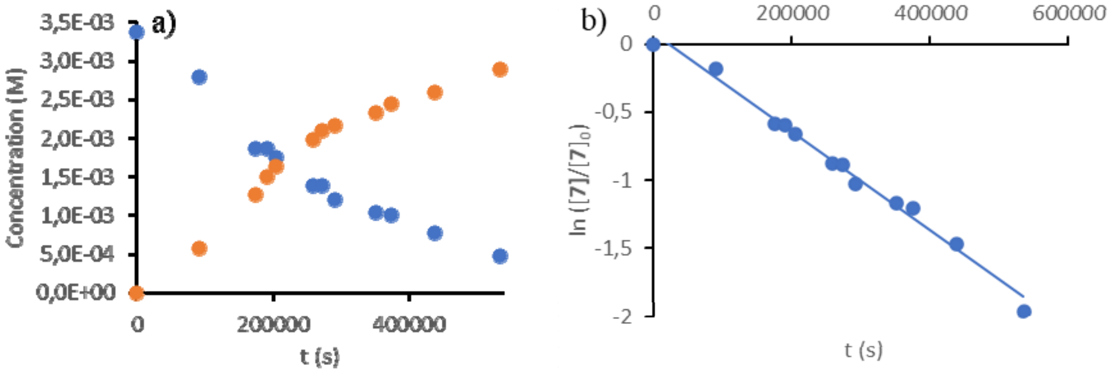

Figure S20. (a) Concentration *versus* time plot for the reaction of **7** (blue circles) with fluorobenzene to give **11** (orange circles) at 343 K. (b) Linearized fit for the disappearance of **7**.

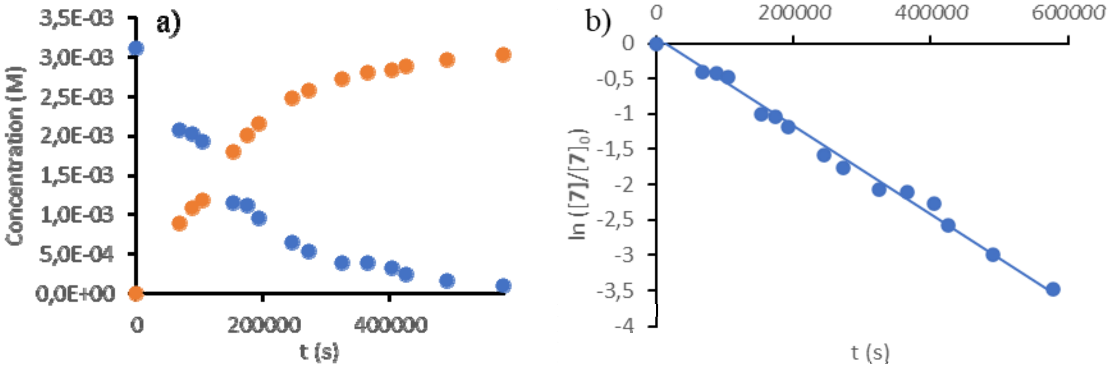

Figure S21. (a) Concentration *versus* time plot for the reaction of **7** (blue circles) with fluorobenzene to give **11** (orange circles) at 348 K. (b) Linearized fit for the disappearance of **7**.

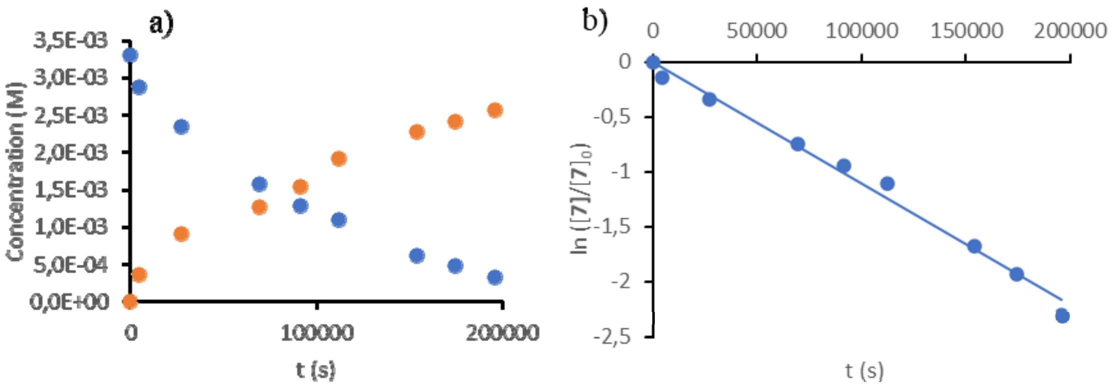

Figure S22. (a) Concentration *versus* time plot for the reaction of **7** (blue circles) with fluorobenzene to give **11** (orange circles) at 353 K. (b) Linearized fit for the disappearance of **7**.

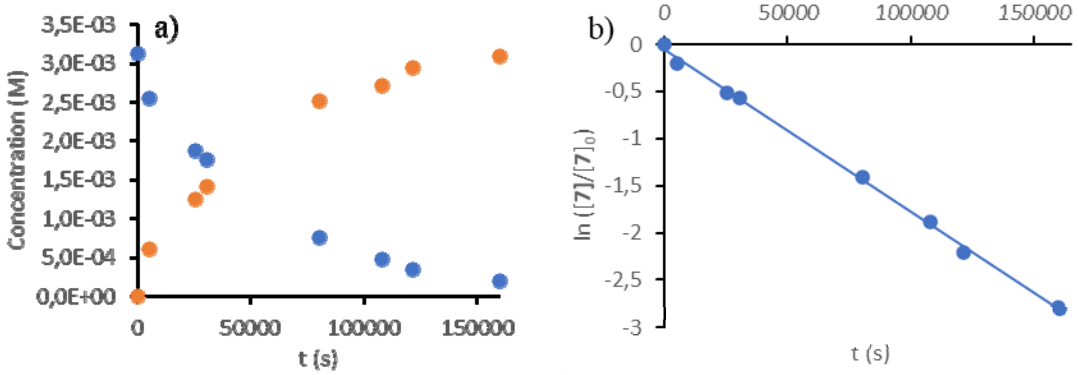

Figure S23. (a) Concentration *versus* time plot for the reaction of **7** (blue circles) with fluorobenzene to give **11** (orange circles) at 358 K. (b) Linearized fit for the disappearance of **7**.

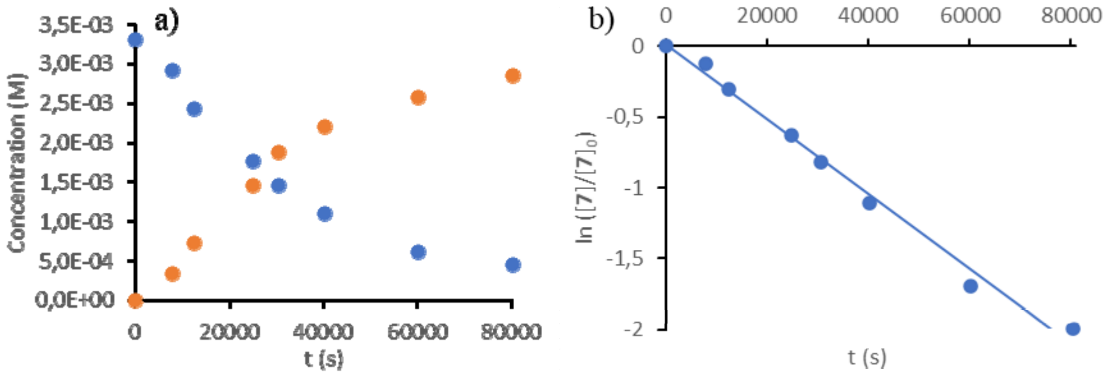

Figure S24. (a) Concentration *versus* time plot for the reaction of **7** (blue circles) with fluorobenzene to give **11** (orange circles) at 363 K. (b) Linearized fit for the disappearance of **7**.

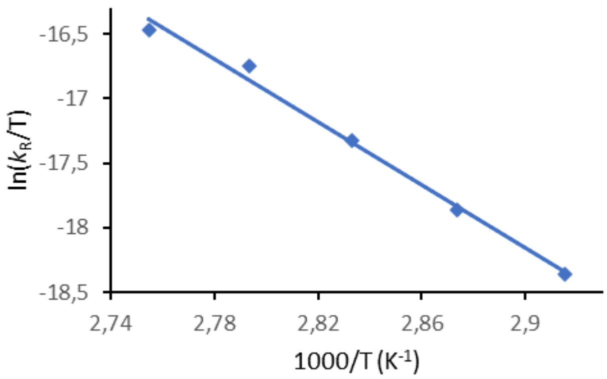

Figure S25. Eyring plot for the transformation of **7** into **11**.

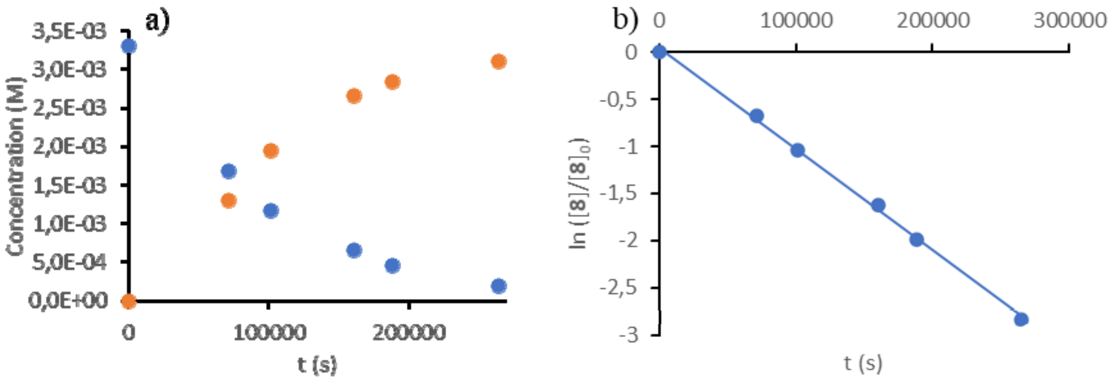

Figure S26. (a) Concentration *versus* time plot for the reaction of **8** (blue circles) with fluorobenzene to give **11** (orange circles) at 338 K. (b) Linearized fit for the disappearance of **8**.

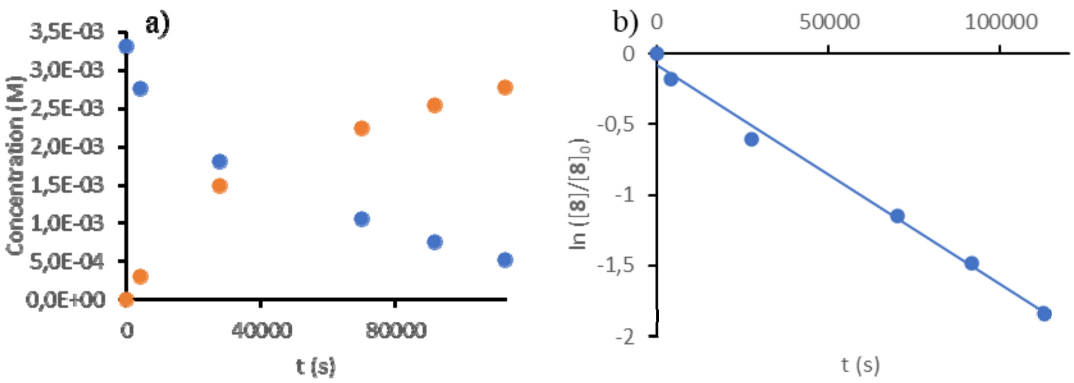

Figure S27. (a) Concentration *versus* time plot for the reaction of **8** (blue circles) with fluorobenzene to give **11** (orange circles) at 353 K. (b) Linearized fit for the disappearance of **8**.

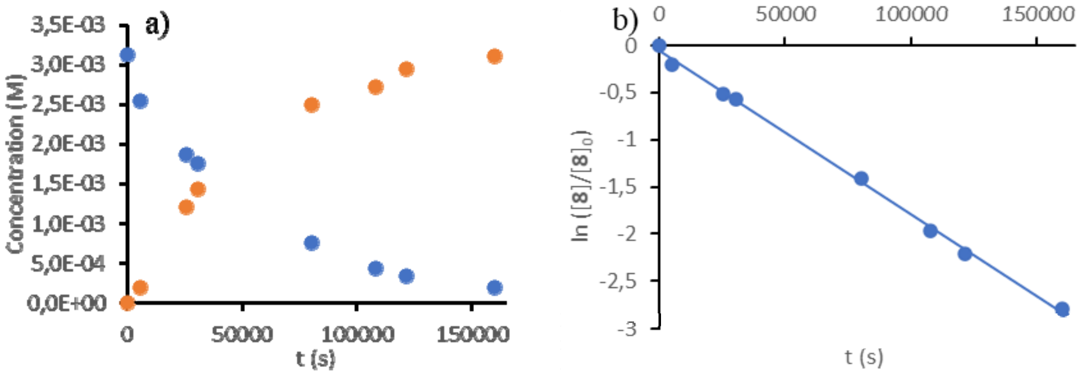

Figure S28. (a) Concentration *versus* time plot for the reaction of **8** (blue circles) with fluorobenzene to give **11** (orange circles) at 358 K. (b) Linearized fit for the disappearance of **8**.

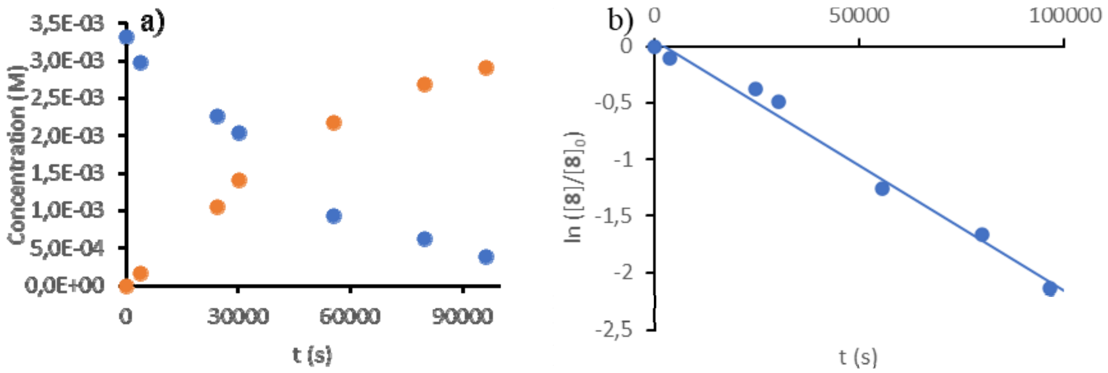

Figure S29. (a) Concentration *versus* time plot for the reaction of **8** (blue circles) with fluorobenzene to give **11** (orange circles) at 363 K. (b) Linearized fit for the disappearance of **8**.

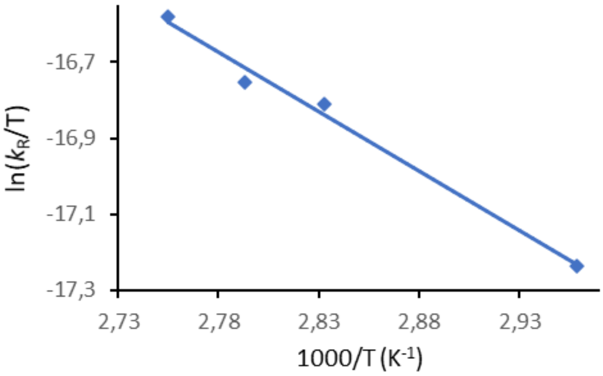

Figure S30. Eyring plot for the transformation of **8** into **11**.

• Structural Analysis of Complexes 2, 3, 4, 5b, 6, 8 and 11a.

X-ray data were collected on Bruker APEX CCD (**2**, **3**, **4**, **8**), DUO CCD (**5b**) and D8 Venture (**6**, **11a**) diffractometers (Mo radiation, λ = 0.71073 Å). The crystals were cooled with a nitrogen flow from Oxford Cryosystems systems. Data were corrected for absorption by using a multiscan method applied with the SADABS program.[3] The structures were solved by Patterson or direct methods and refined by full-matrix least squares on F^2 with SHELXL2016,[4] including isotropic and subsequently anisotropic displacement parameters. The hydrogen atoms were observed in the last Fourier Maps or calculated, and refined freely or using a restricted riding model. The hydride ligand on **11a** was located in the last Fourier Maps and refined with restrained rhodium-hydride distance (1.59 Å).

Crystal data for **2** (CCDC 2121582): $C_{38}H_{49}ClNOP_2Rh$, M_W 736.08, colorless, irregular block (0.241 x 0.100 x 0.013 mm^3), monoclinic, space group $P2_1/c$, *a*: 10.0850(7) Å, *b*: 15.1457(10) Å, *c*: 22.9077(15) Å, *β*: 95.7680(10)°, *V* = 3481.3(4) $Å^3$, *Z* = 4, *Z'* = 1, D_{calc}: 1.404 g cm^{-3}, F(000): 1536, T = 100(2) K, μ 0.690 mm^{-1}. 45009 measured reflections (2θ: 3-57°, ω scans 0.3°), 8518 unique (R_{int} = 0.0580); min./max. transm. factors 0.770/0.862. Final agreement factors were R^1 = 0.0358 (6046 observed reflections, I > 2σ(I)) and wR^2 = 0.0892; data/restraints/parameters 8518/0/410; GoF = 1.002. Largest peak and hole 0.716 (close to rhodium atoms) and -0.868 e/ $Å^3$.

Crystal data for **3** (CCDC 2121581): $C_{48}H_{59}ClOP_2Rh$, M_W 852.25, colorless, irregular block (0.300 x 0.211 x 0.067 mm^3), monoclinic, space group $P2_1/c$, *a*: 20.836(4) Å, *b*: 10.605(2) Å, *c*: 20.991(4) Å, *β*: 117.859(2)°, *V* = 4100.7(15) $Å^3$, *Z* = 4, *Z'* = 1, D_{calc}: 1.380 g cm^{-3}, F(000): 1788, T = 100(2) K, μ 0.596 mm^{-1}. 89218 measured reflections (2θ: 3-57°, ω scans 0.3°), 10023 unique (R_{int} = 0.0501); min./max. transm. factors 0.716/0.862. Final agreement factors were R^1 = 0.0298 (8326 observed reflections, I > 2σ(I)) and wR^2 = 0.0748; data/restraints/parameters 10023/0/488; GoF = 1.013. Largest peak and hole 1.037 (close to rhodium atoms) and -0.778 e/ $Å^3$.

Crystal data for **4** (CCDC 2121578): $C_{40}H_{52}ClOP_2Rh$, M_W 749.11, yellow, irregular block (0.158 x 0.117 x 0.065 mm^3), monoclinic, space group $P2_1/c$, *a*: 10.7509(6) Å, *b*: 14.7695(8) Å, *c*: 22.6636(12) Å, *β*: 92.5560(10)°, *V* = 3595.1(3) $Å^3$, *Z* = 4, *Z'* = 1, D_{calc}:

1.384 g cm^{-3}, F(000): 1568, T = 100(2) K, μ 0.669 mm^{-1}. 31463 measured reflections (2θ: 3-57°, ω scans 0.3°), 8648 unique (R_{int} = 0.0378); min./max. transm. factors 0.740/0.862. Final agreement factors were R^1 = 0.0347 (7361 observed reflections, I > 2σ(I)) and wR^2 = 0.0767; data/restraints/parameters 8648/0/422; GoF = 1.064. Largest peak and hole 0.511 (close to rhodium atoms) and -0.705 e/ Å^3.

Crystal data for **5b** (CCDC 2121579): $C_{34}H_{47}Cl_2OP_2Rh$, M_W 707.46, yellow, irregular block (0.168 x 0.161 x 0.155 mm^3), orthorhombic, space group Pna2_1, *a*: 12.2527(10) Å, *b*: 28.601(2) Å, *c*: 18.5546(15) Å, *V* = 6502.3(9) Å^3, *Z* = 8, *Z'* = 2, D_{calc}: 1.445 g cm^{-3}, F(000): 2944, T = 120(2) K, μ 0.814 mm^{-1}. 72689 measured reflections (2θ: 3-57°, ω scans 0.3°), 18702 unique (R_{int} = 0.0541); min./max. transm. factors 0.753/0.862. Final agreement factors were R^1 = 0.0421 (15751 observed reflections, I > 2σ(I)) and wR^2 = 0.0950; Flack parameter -0.01(3); data/restraints/parameters 18702/1/ 742; GoF = 1.018. Largest peak and hole 1.216 (close to rhodium atoms) and -0.621 e/ Å^3.

Crystal data for **6** (CCDC 2121584): $C_{38}H_{49}NOP_2Rh$, BF_4, C_3H_6O, M_W 845.52, colorless, irregular block (0.118 x 0.098 x 0.095 mm^3), triclinic, space group P-1, *a*: 11.909(5) Å, *b*: 12.939(6) Å, *c*: 14.516(5) Å, *α*: 92.238(15)°, *β*: 109.634(11) °, *γ*: 100.571(15) °, *V* = 2058.7(16) Å^3, *Z* = 2, *Z'* = 1, D_{calc}: 1.364 g cm^{-3}, F(000): 880, T = 100(2) K, μ 0.545 mm^{-1}. 135165 measured reflections (2θ: 3-57°, ω scans 0.3°), 12506 unique (R_{int} = 0.0286); min./max. transm. factors 0.830/0.862. Final agreement factors were R^1 = 0.0291 (12077 observed reflections, I > 2σ(I)) and wR^2 = 0.0778; data/restraints/parameters 12506/0/481; GoF = 1.093. Largest peak and hole 1.458 (close to rhodium atoms) and -0.444 e/ Å^3.

Crystal data for **8** (CCDC 2121580): $C_{40}H_{52}OP_2Rh$ x BF_4 x 0.5(C_3H_6O), M_W 829.51, yellow, irregular block (0.178 x 0.152 x 0.091 mm^3), triclinic, space group P-1, *a*: 10.5782(7) Å, *b*: 11.4356(8) Å, *c*: 16.3989(11) Å, *α*: 89.1520(10)°, *β*: 80.5180(10) °, *γ*: 87.5790(10)°, *V* = 1954.8(2) Å^3, *Z* = 2, *Z'* = 1, D_{calc}: 1.409 g cm^{-3}, F(000): 864, T = 100(2) K, μ 0.571 mm^{-1}. 34428 measured reflections (2θ: 3-57°, ω scans 0.3°), 9326 unique (R_{int} = 0.0476); min./max. transm. factors 0.765/0.862. Final agreement factors were R^1 = 0.0553 (6853 observed reflections, I > 2σ(I)) and wR^2 = 0.1437;

data/restraints/parameters 9326/36/ 465; GoF = 1.079. Largest peak and hole 1.312 (close to rhodium atoms) and -1.163 e/ Å^3.

Crystal data for **11a** (CCDC 2121583): $C_{33}H_{45}BF_5OP_2Rh$ x C_6H_5F, M_W 824.45, colourless, irregular block (0.100 x 0.085 x 0.045 mm^3), monoclinic, space group C2/c, *a*: 41.197(2) Å, *b*: 10.1572(5) Å, *c*: 18.4963(10) Å, *β*: 103.688(2)°, *V* = 7519.8(7) Å^3, *Z* = 8, *Z'* = 1, D_{calc}: 1.456 g cm^{-3}, F(000): 3408, T = 100(2) K, μ 0.600 mm^{-1}. 116634 measured reflections (2θ: 3-57°, ω scans 0.3°), 11507 unique (R_{int} = 0.0416); min./max. transm. factors 0.810/0.862. Final agreement factors were R^1 = 0.0259 (10503 observed reflections, I > 2σ(I)) and wR^2 = 0.0657; data/restraints/parameters 11507/1/482; GoF = 1.013. Largest peak and hole 1.129 (close to rhodium atoms) and -0.603 e/ Å^3.

(1) Esteruelas, M. A.; Oliván, M.; Vélez, A. POP-Rhodium-Promoted C-H and B-H Bond Activation and C-B-Bond Formation. *Organometallics* **2015**, *34*, 1911-1924.

(2) Esteruelas, M. A.; Oliván, M.; Vélez, A. Xantphos-Type Complexes of Group 9: Rhodium versus Iridium. *Inorg. Chem.* **2013**, *52*, 5339-5349.

(3) Blessing, R. H. *Acta Crystallogr.* **1995**, *A51*, 33. SADABS: Area-detector absorption correction; Bruker- AXS, Madison, WI, 1996.

(4) SHELXL-2016/6. Sheldrick, G. M. *Acta Cryst.* **2008**, *A64*, 112-122.

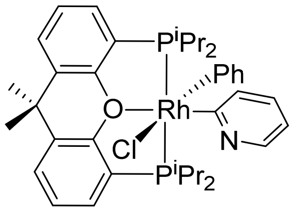

2

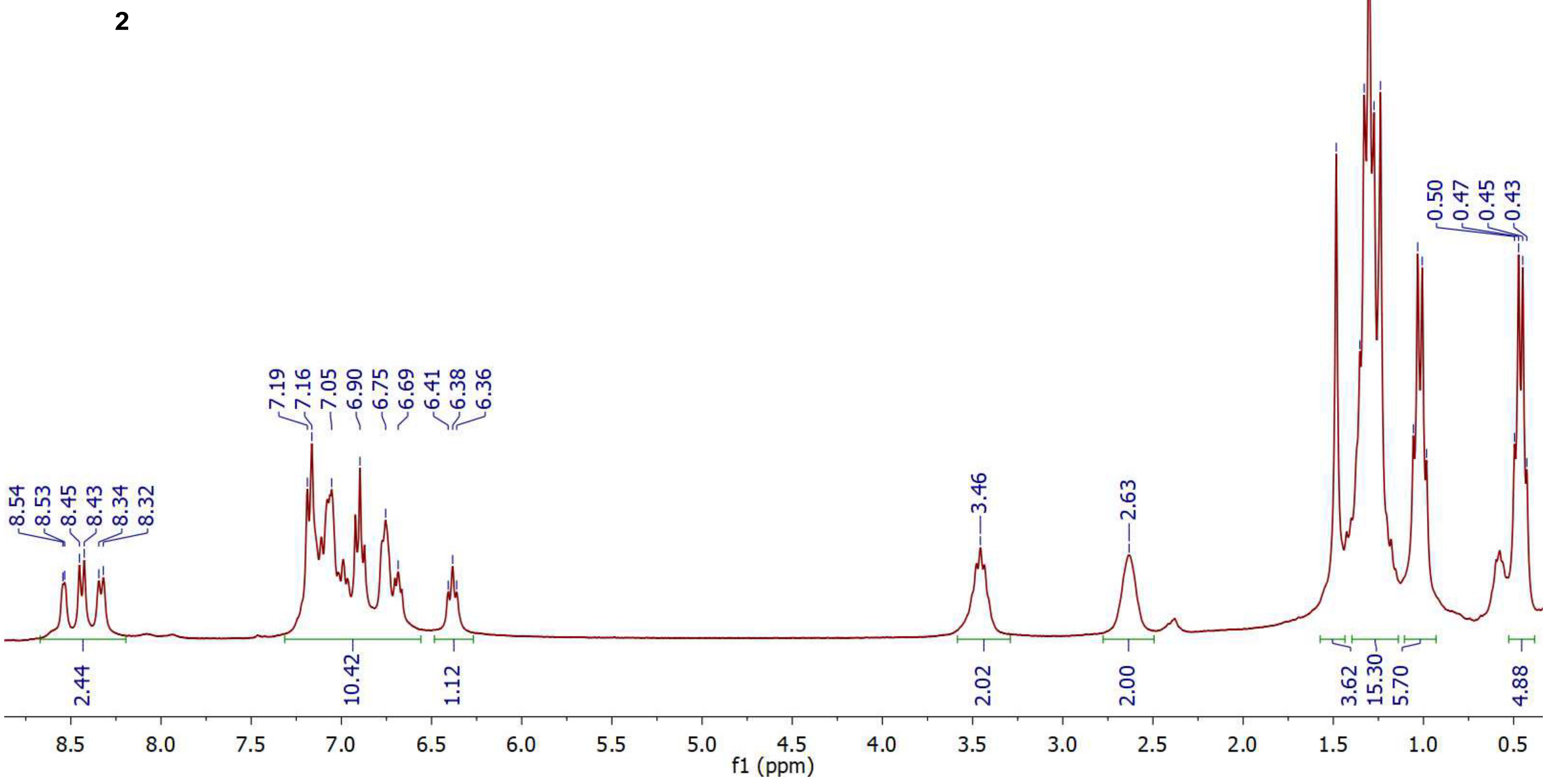

Figure S31. ^{1}H NMR spectrum (300.13 MHz, C_6D_6, 298 K) of Rh(Ph)(2-pyridyl)Cl{κ^3-P,O,P-[xant(P^iPr_2)$_2$]} (**2**).

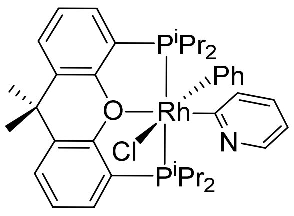

2

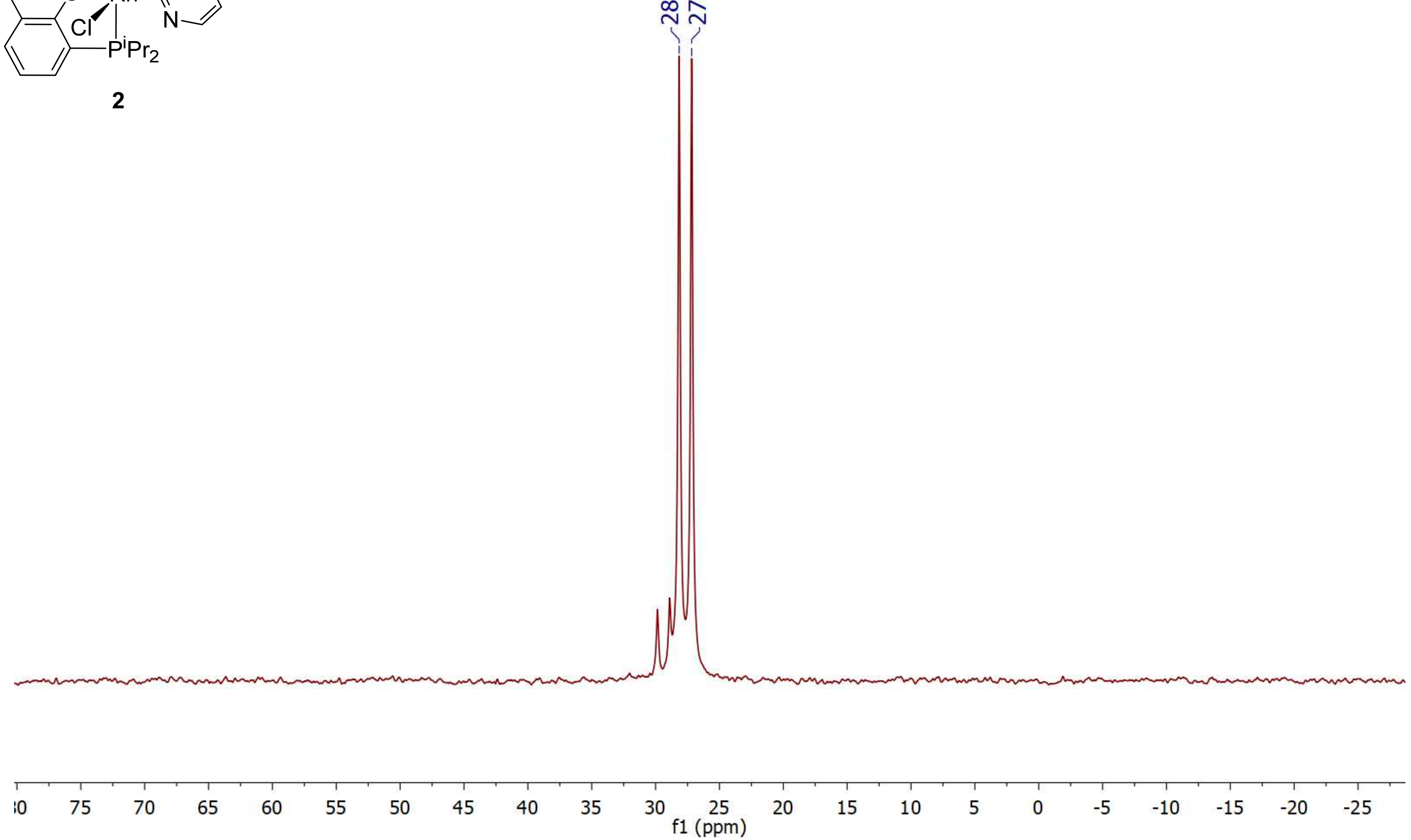

Figure S32. $^{31}P\{^1H\}$ NMR (121.49 MHz, C_6D_6, 298 K) of Rh(Ph)(2-pyridyl)Cl{κ^3-P,O,P-[xant(P^{i}Pr$_2$)$_2$]} (**2**).

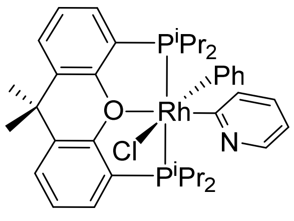

2

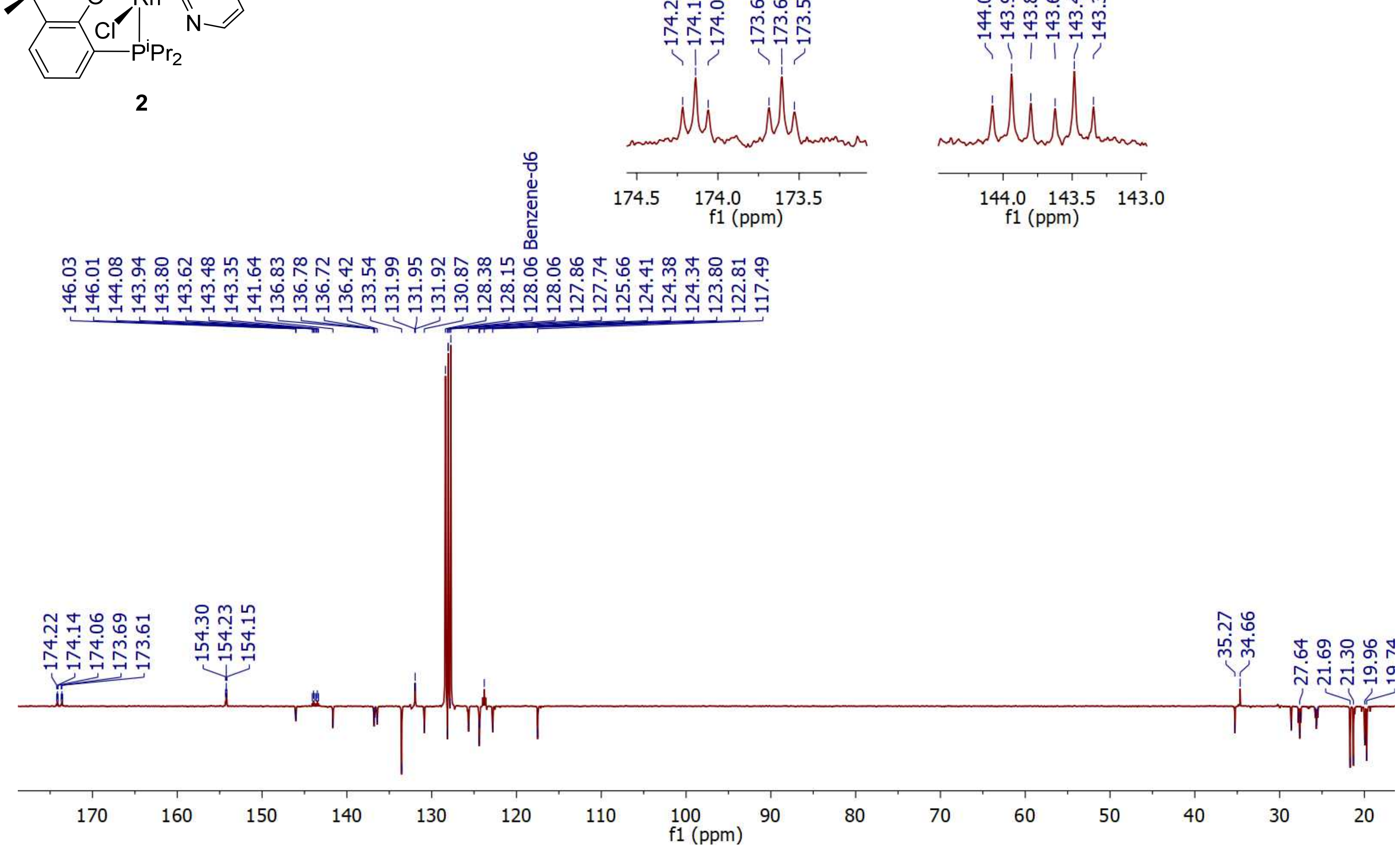

Figure S33. $^{13}C\{^1H\}$-apt NMR (75.48 MHz, C_6D_6, 298 K) of Rh(Ph)(2-pyridyl)Cl{κ^3-P,O,P-[xant(P^{i}Pr$_2$)$_2$]} (**2**).

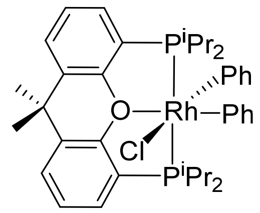

3

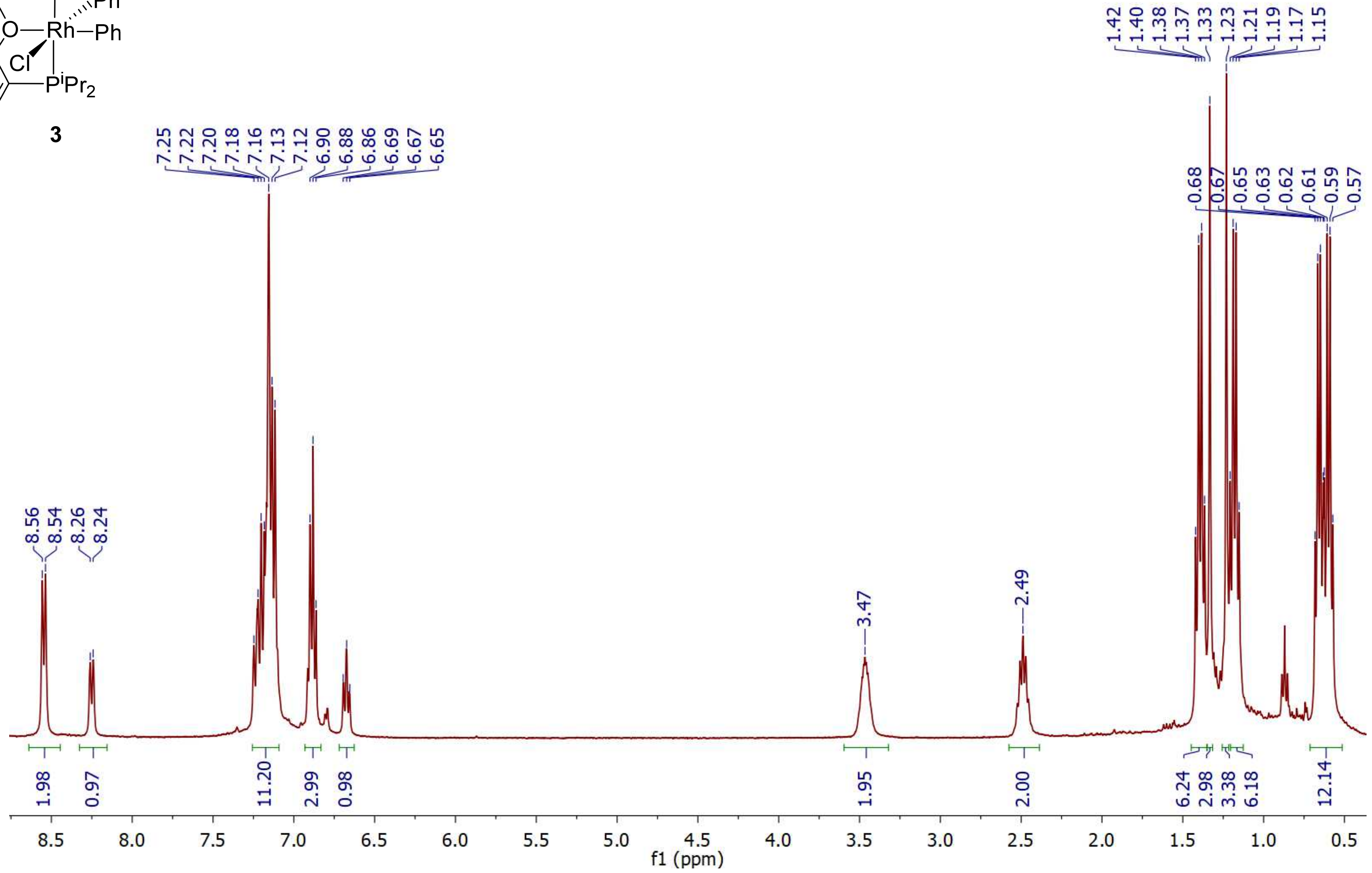

Figure S34. ^{1}H NMR spectrum (400.16 MHz,MHz, C_6D_6, 298 K) of $RhPh_2Cl\{\kappa^3$-P,O,P-[xant(P^iPr_2)$_2$]$\}$ (**3**).

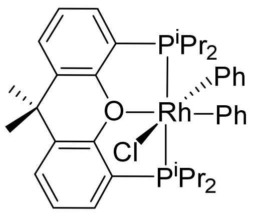

3

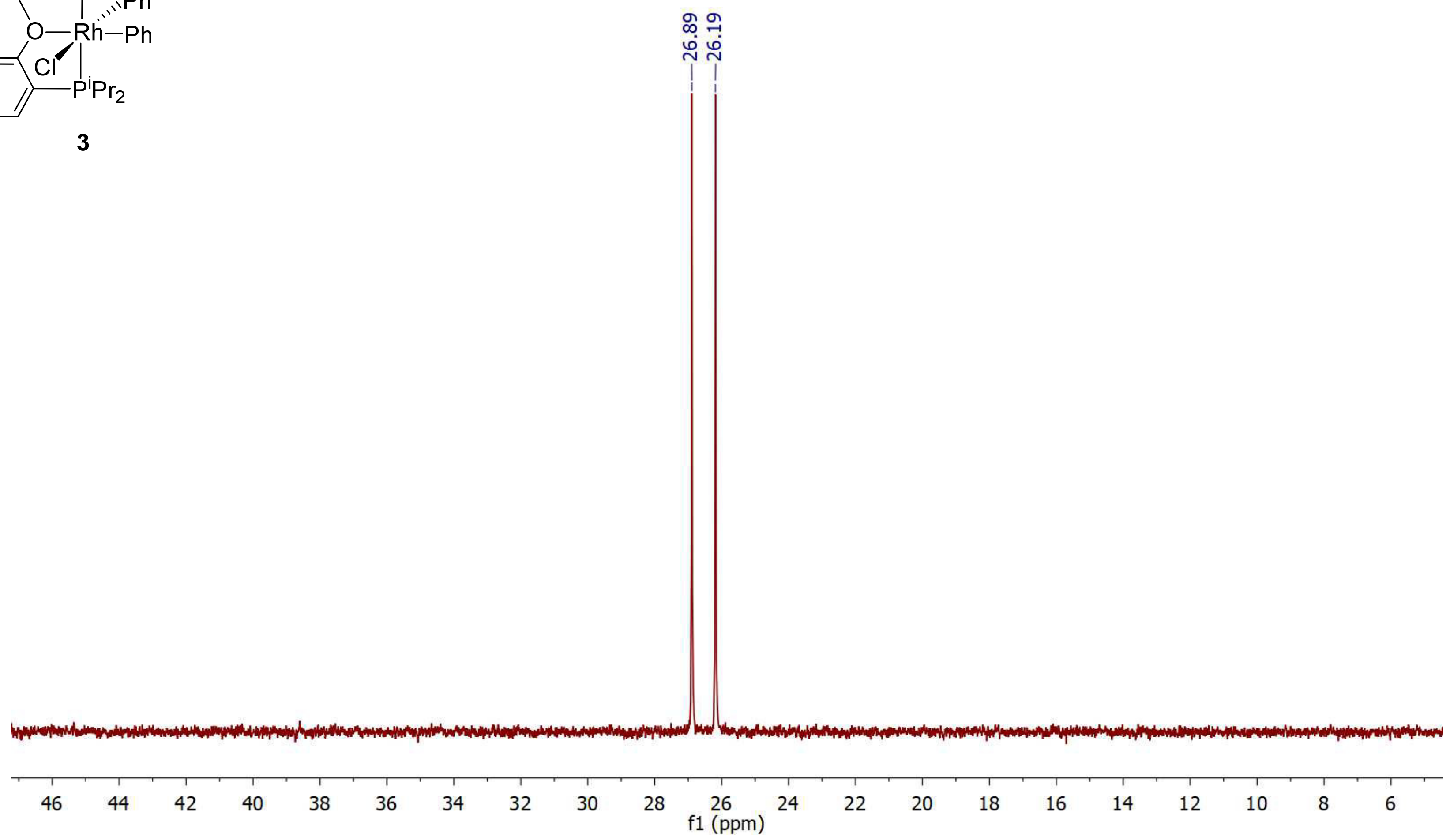

Figure S35. $^{31}P\{^{1}H\}$ NMR (161.99 MHz, C_6D_6, 298 K) of $RhPh_2Cl\{\kappa^3$-P,O,P-[xant($P^iPr_2)_2$]} (**3**).

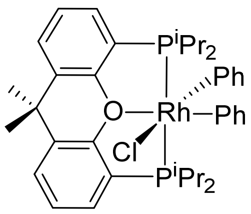

3

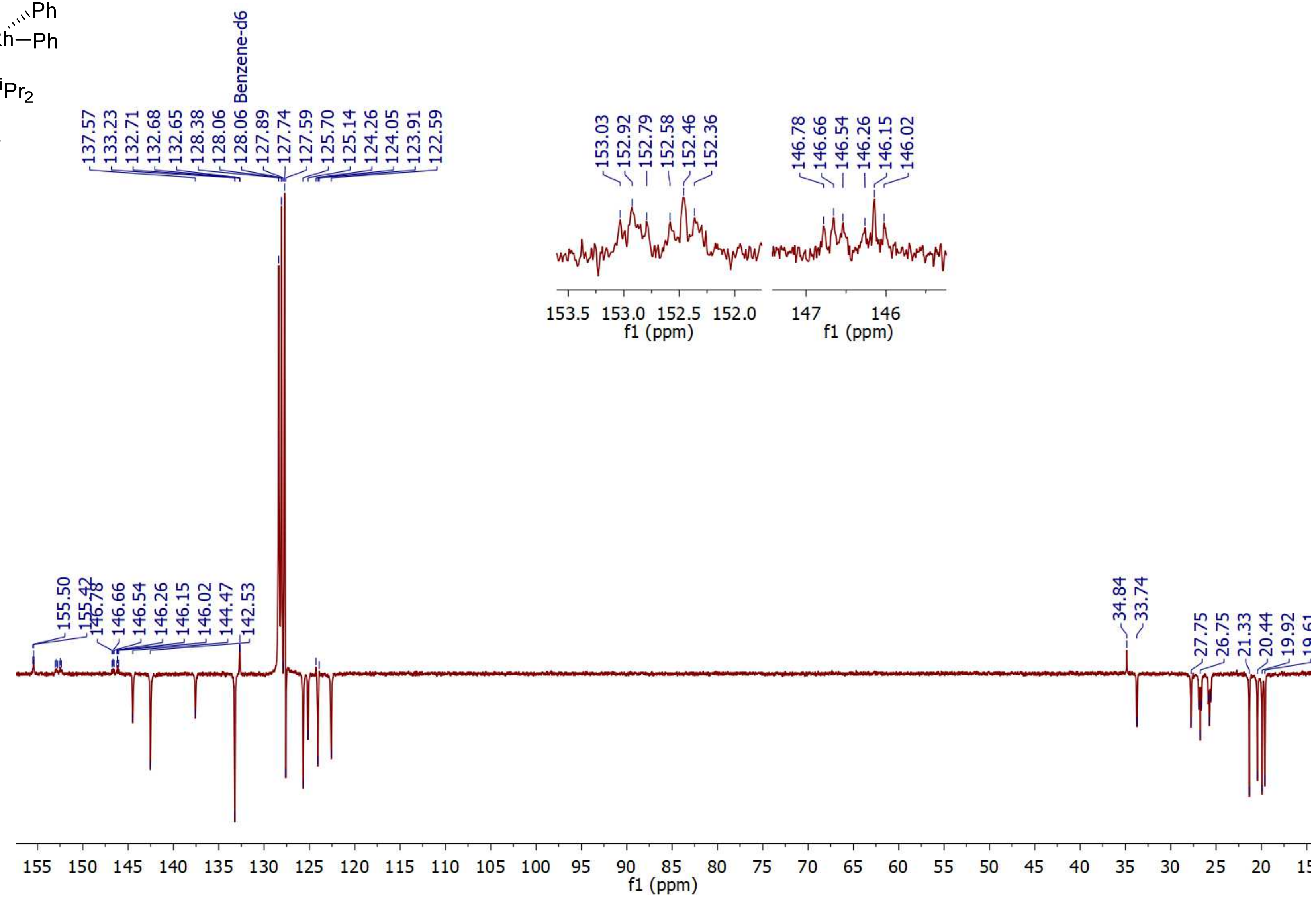

Figure S36. $^{13}C\{^{1}H\}$-apt NMR (75.48 MHz, C_6D_6, 298 K) of $RhPh_2Cl\{\kappa^3$-P,O,P-[xant($P^iPr_2)_2$]} (**3**).

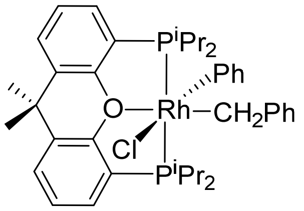

4

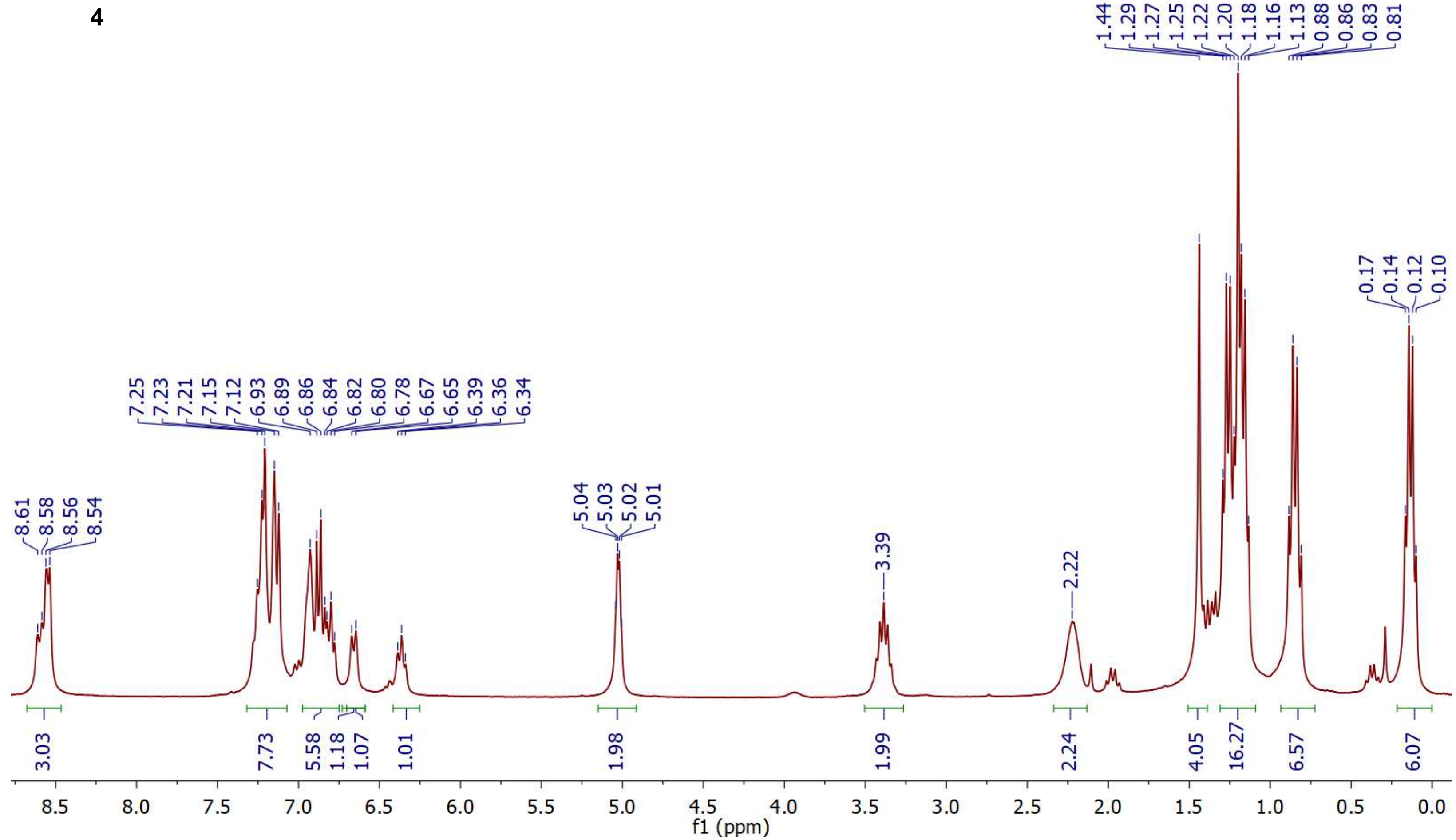

Figure S37. ^{1}H NMR spectrum (300.13 MHz, C_6D_6, 298 K) of RhPh(CH_2Ph)Cl{κ^3-P,O,P-[xant(P^iPr_2)$_2$]} (**4**).

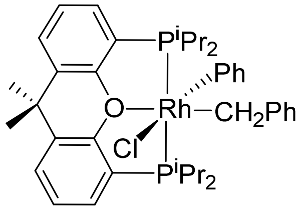

4

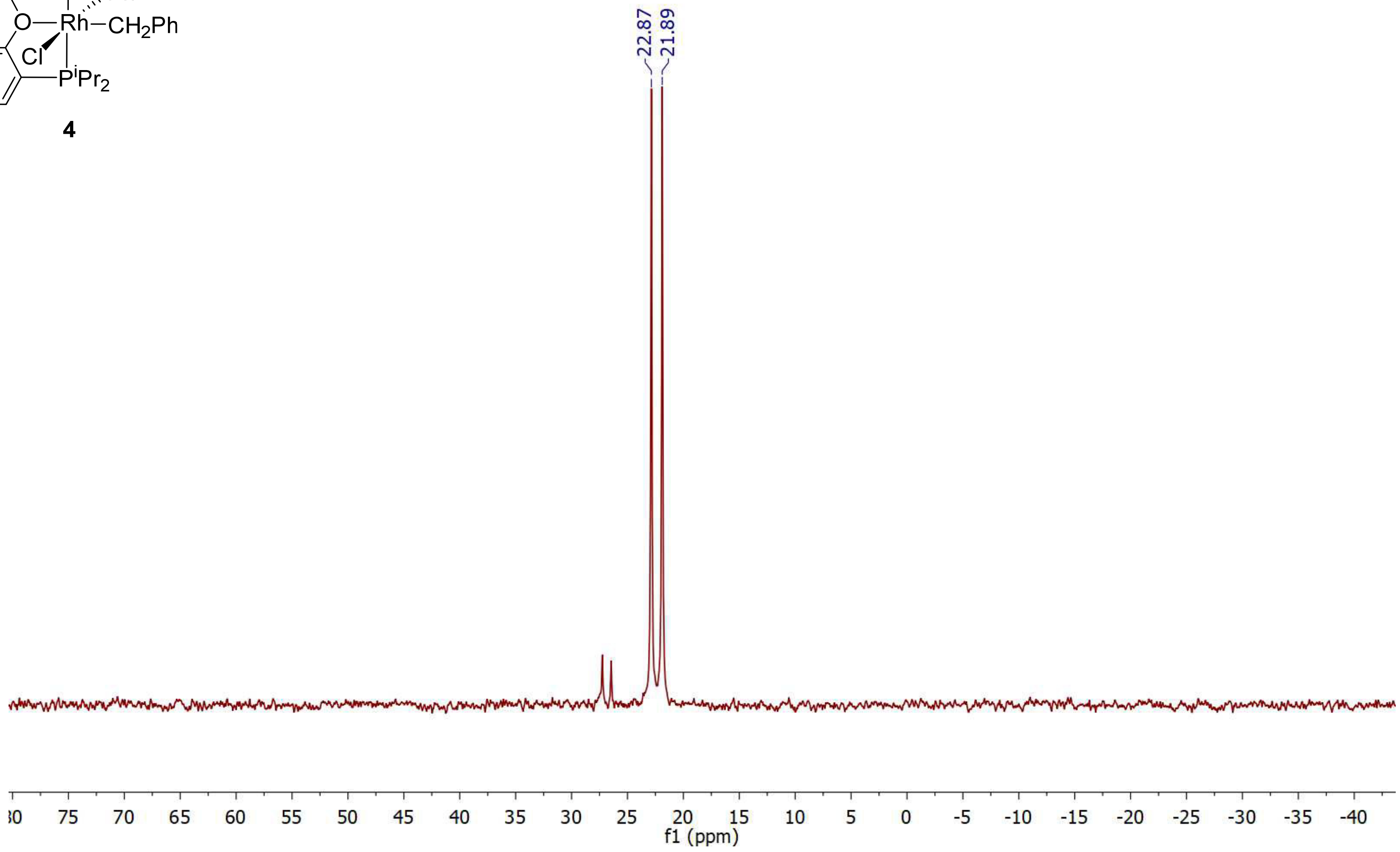

Figure S38. $^{31}P\{^1H\}$ NMR (121.49 MHz, C_6D_6, 298 K) of $RhPh(CH_2Ph)Cl\{\kappa^3$-P,O,P-[xant($P^iPr_2)_2$]} (**4**).

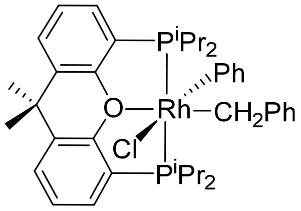

4

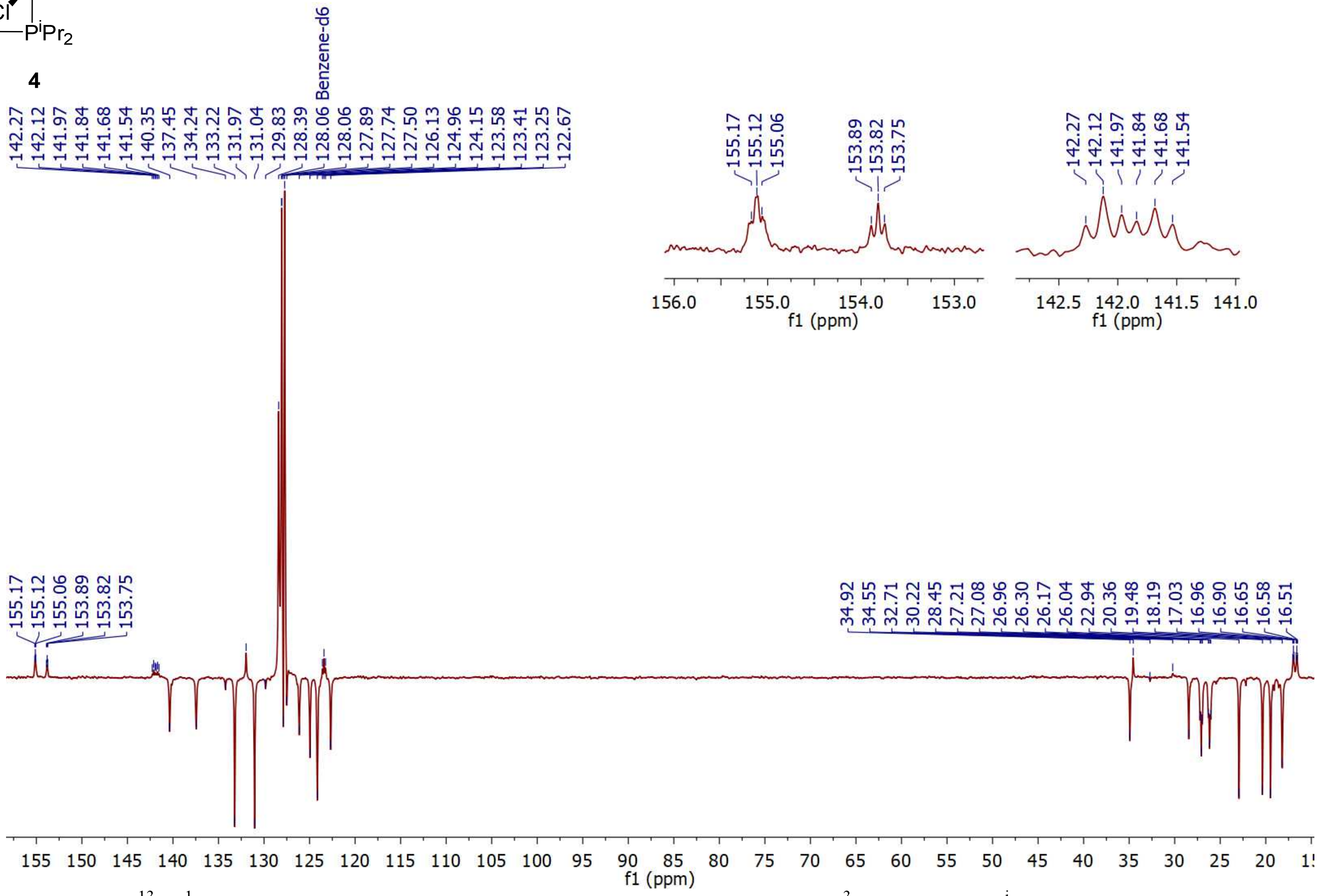

Figure S39. $^{13}C\{^{1}H\}$-apt NMR (75.48 MHz, C_6D_6, 298 K) of $RhPh(CH_2Ph)Cl\{\kappa^3$-P,O,P-[xant($P^iPr_2)_2$]} (**4**).

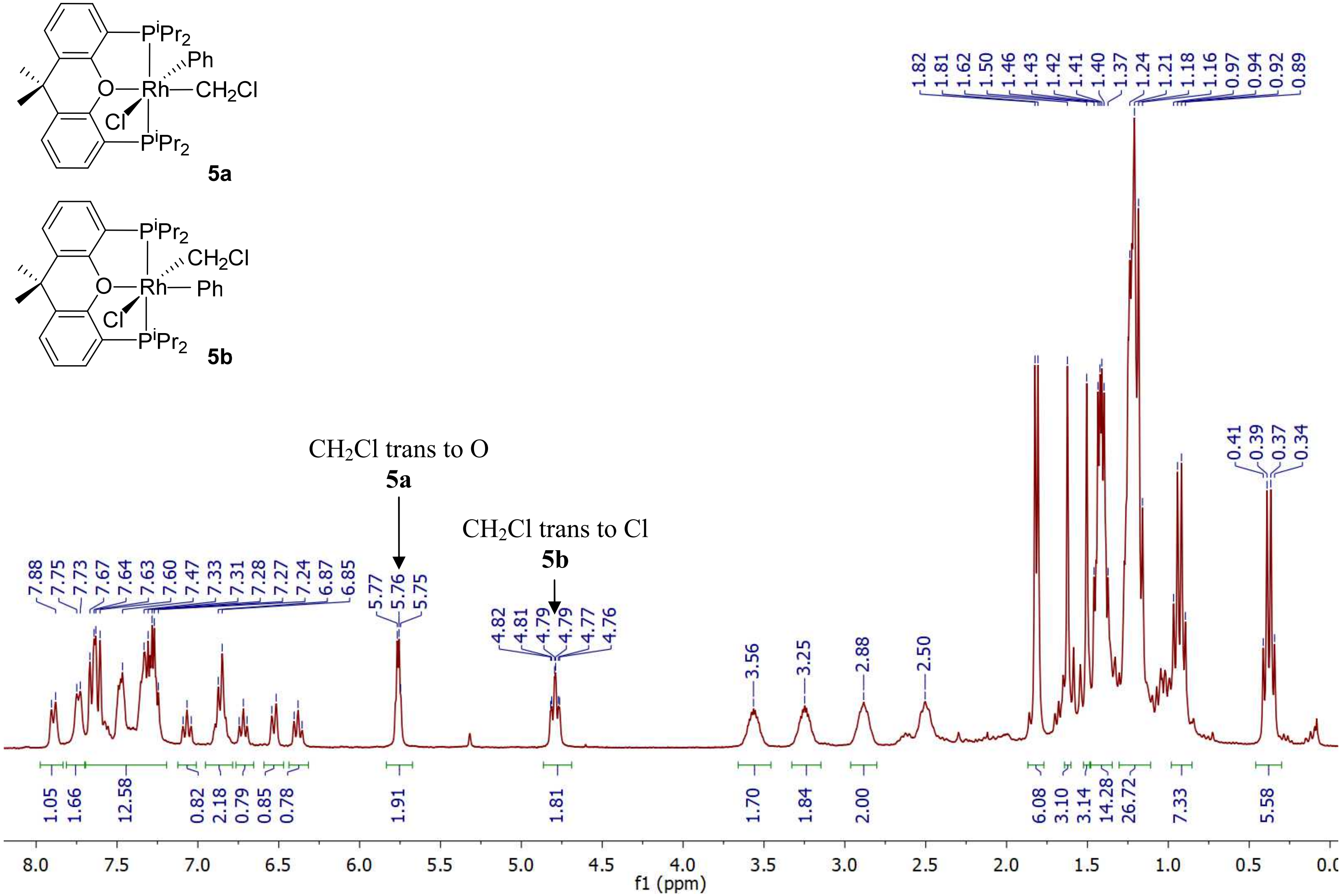

Figure S40. ^{1}H NMR spectrum (300.13 MHz, CD_2Cl_2, 298 K) of RhPh(CH_2Cl)Cl{κ^3-P,O,P-[xant(P^iPr_2)$_2$]} (**5a**,**5b**).

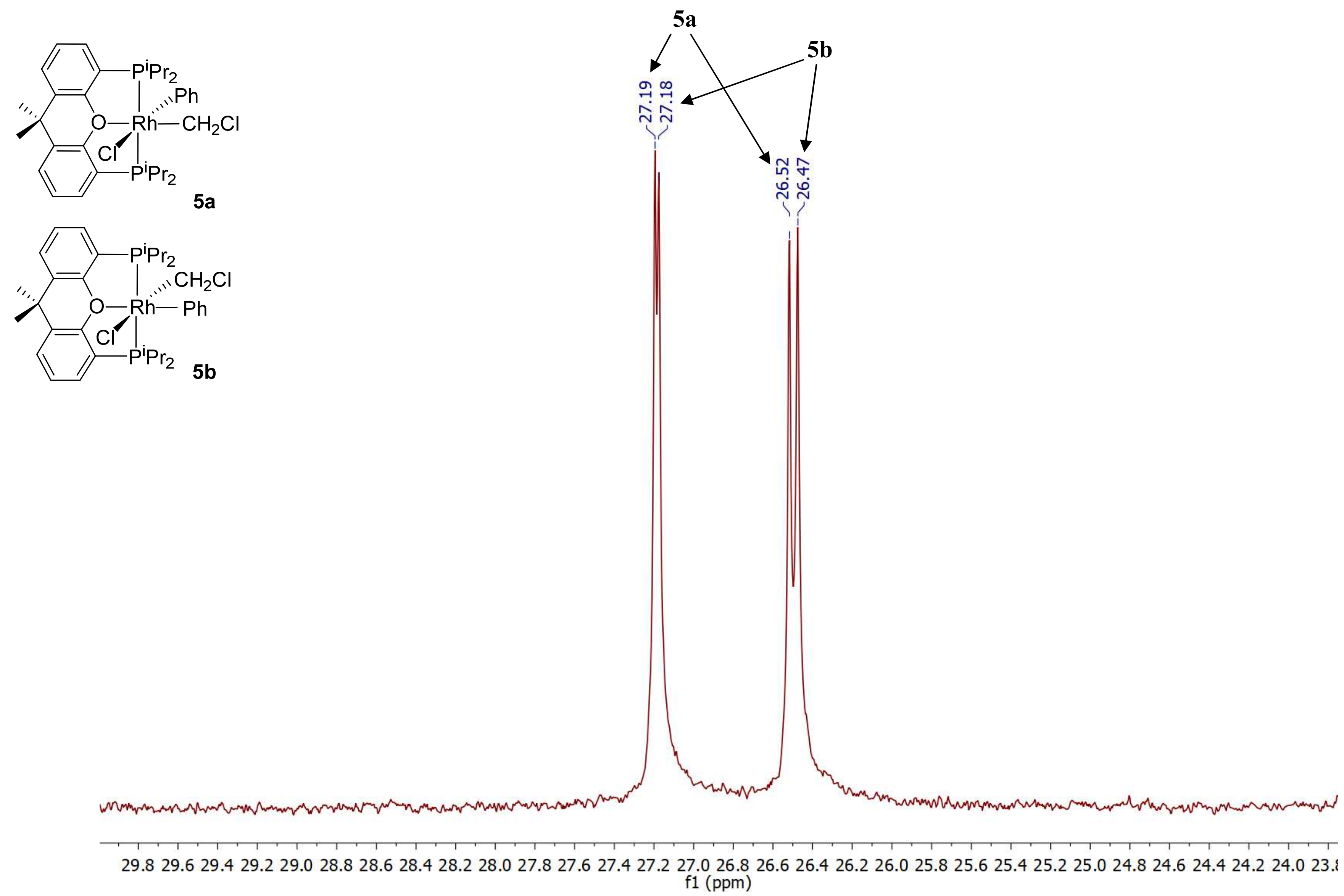

Figure S41. $^{31}P\{^1H\}$ NMR spectrum (161.99 MHz, CD_2Cl_2, 298 K) of RhPh(CH_2Cl)Cl{κ^3-P,O,P-[xant(P^iPr_2)$_2$]} (**5a**,**5b**).

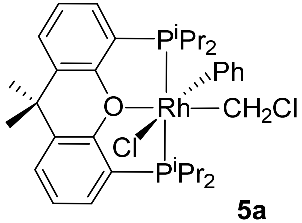

5a

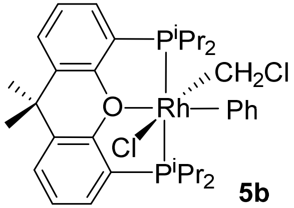

5b

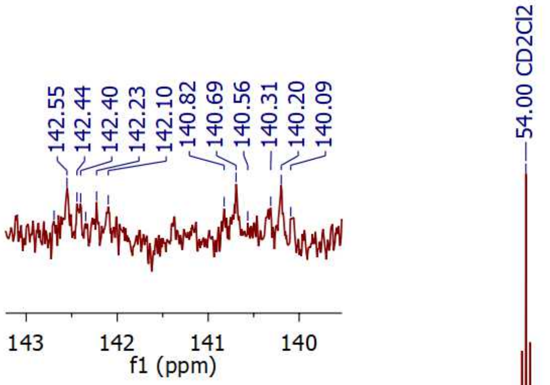
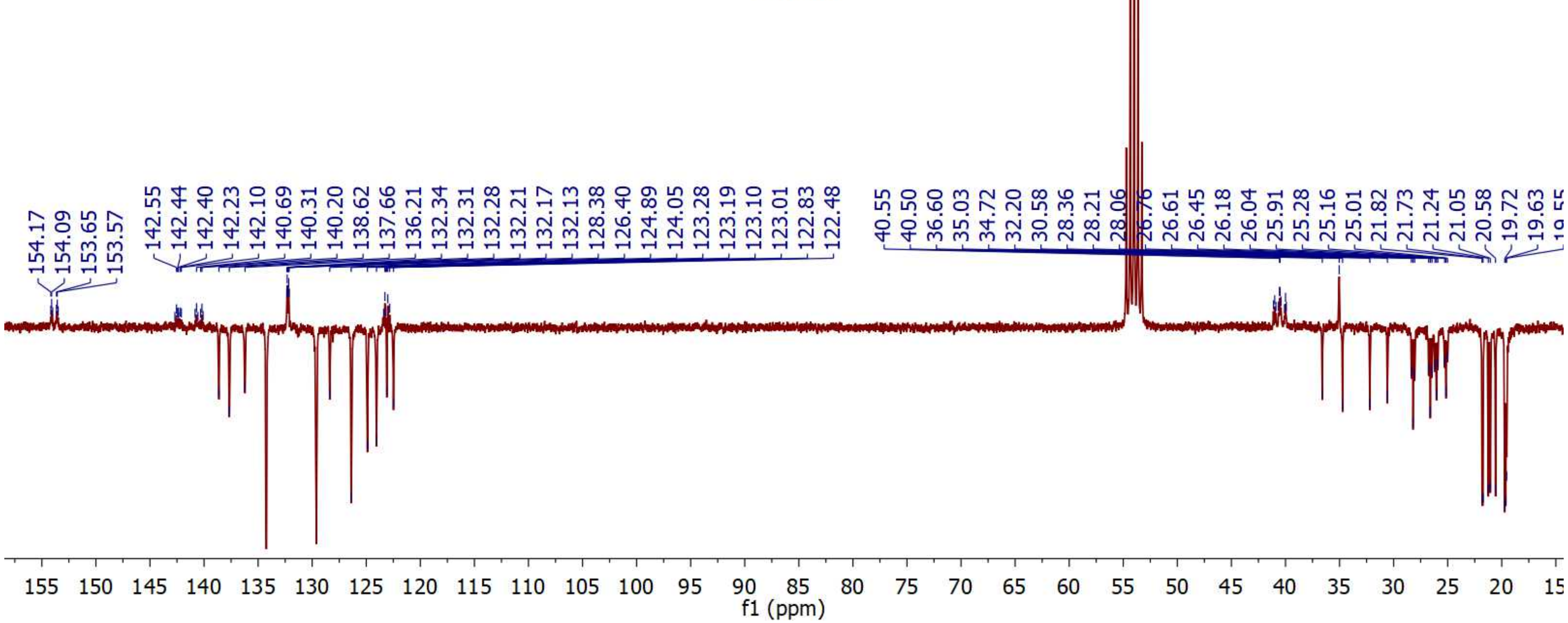

Figure S42. $^{13}C\{^1H\}$-apt NMR spectrum (75.48 MHz, CD_2Cl_2, 298 K) of RhPh(CH_2Cl)Cl{κ^3-P,O,P-[xant(P^iPr_2)$_2$]} (**5a**,**5b**).

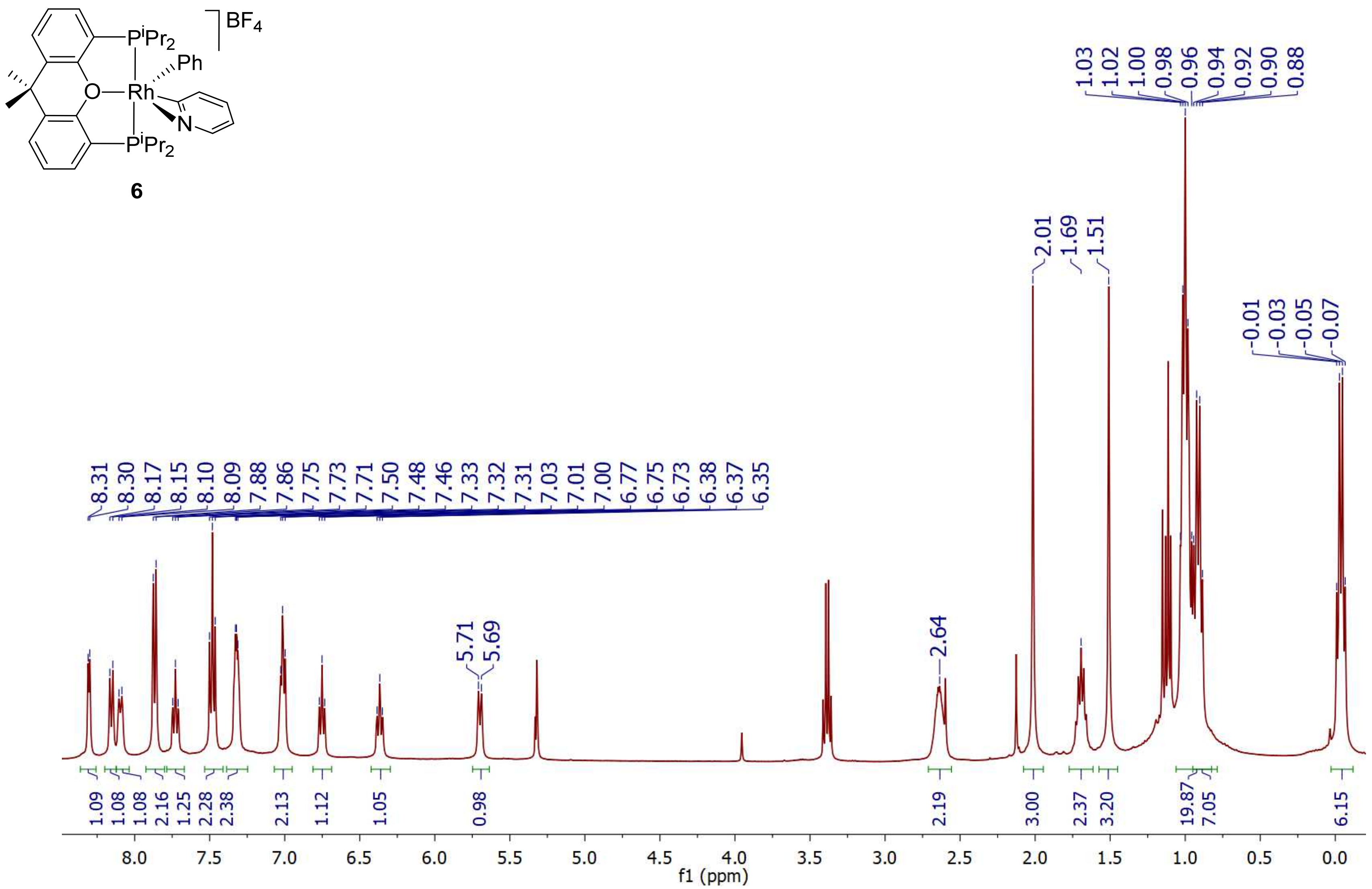

Figure S43. ^{1}H NMR spectrum (400.13 MHz, CD_2Cl_2, 233 K) of [RhPh(η^2-C,N-NC_5H_4){κ^3-P,O,P-[xant(P^iPr_2)$_2$]}]BF_4 (**6**).

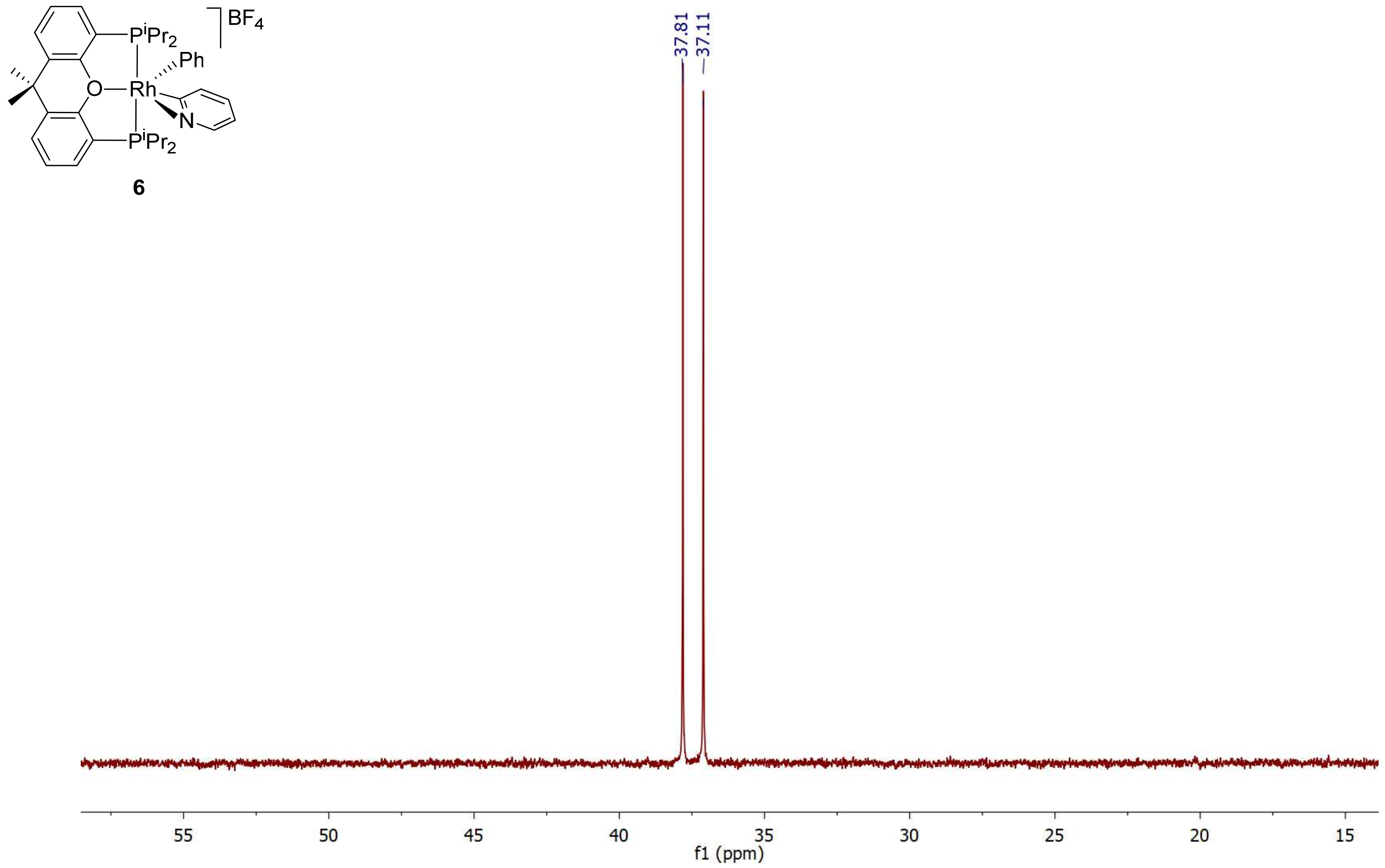

Figure S44. $^{31}P\{^{1}H\}$ NMR spectrum (161.98 MHz, CD_2Cl_2, 233 K) of [RhPh(η^2-C,N-NC_5H_4){κ^3-P,O,P-[xant(P^iPr_2)$_2$]}]BF_4 (**6**).

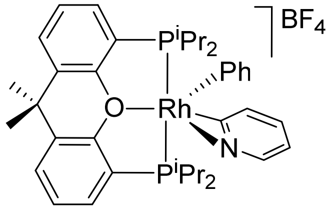

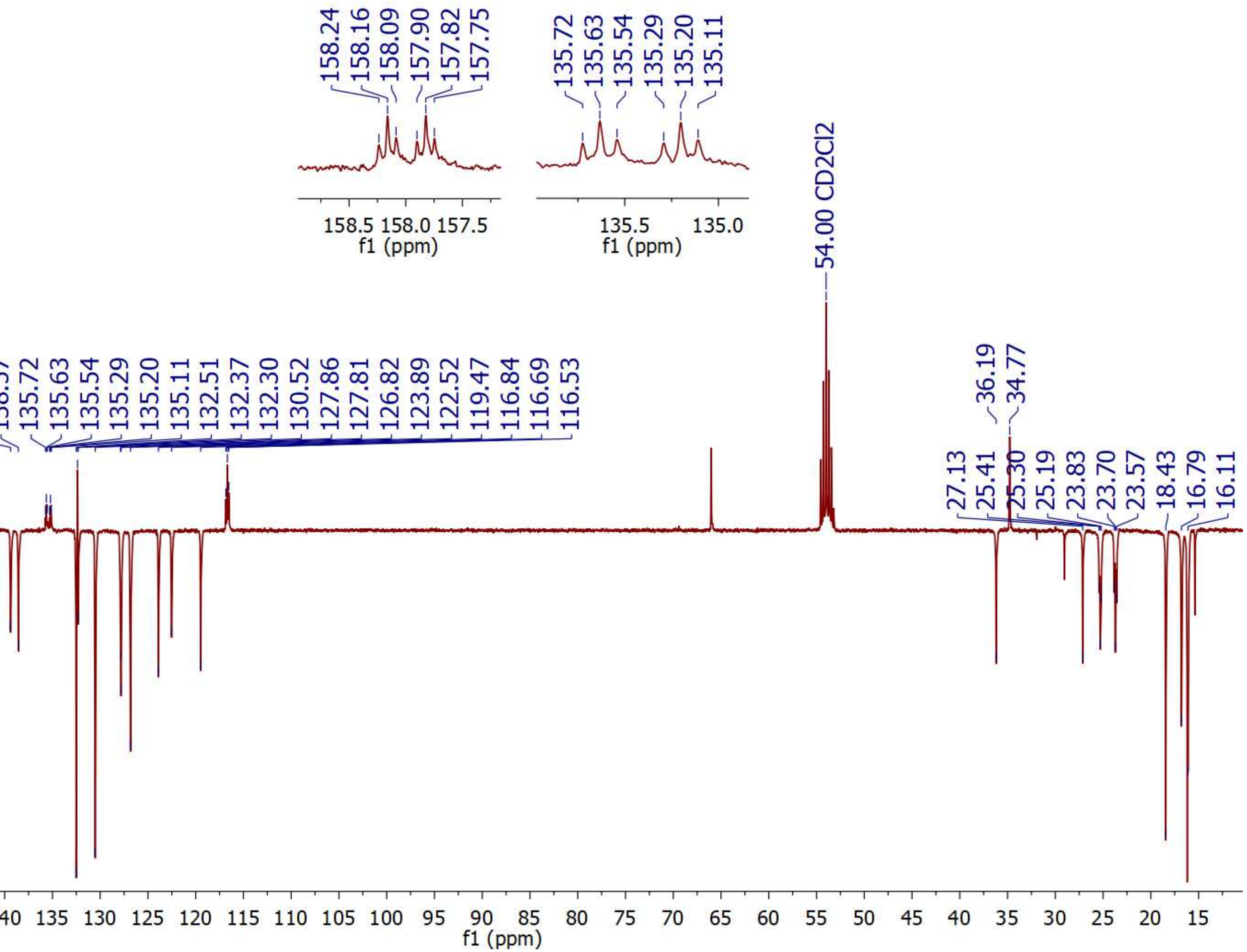

Figure S45. $^{13}C\{^1H\}$-apt NMR spectrum (100.62 MHz, CD_2Cl_2, 233 K) of [RhPh(η^2-C,N-NC_5H_4){κ^3-P,O,P-[xant(P^iPr_2)$_2$]}]BF_4 (**6**).

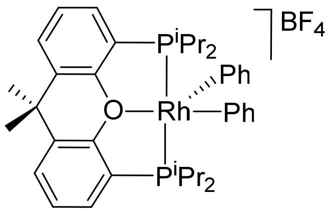

7

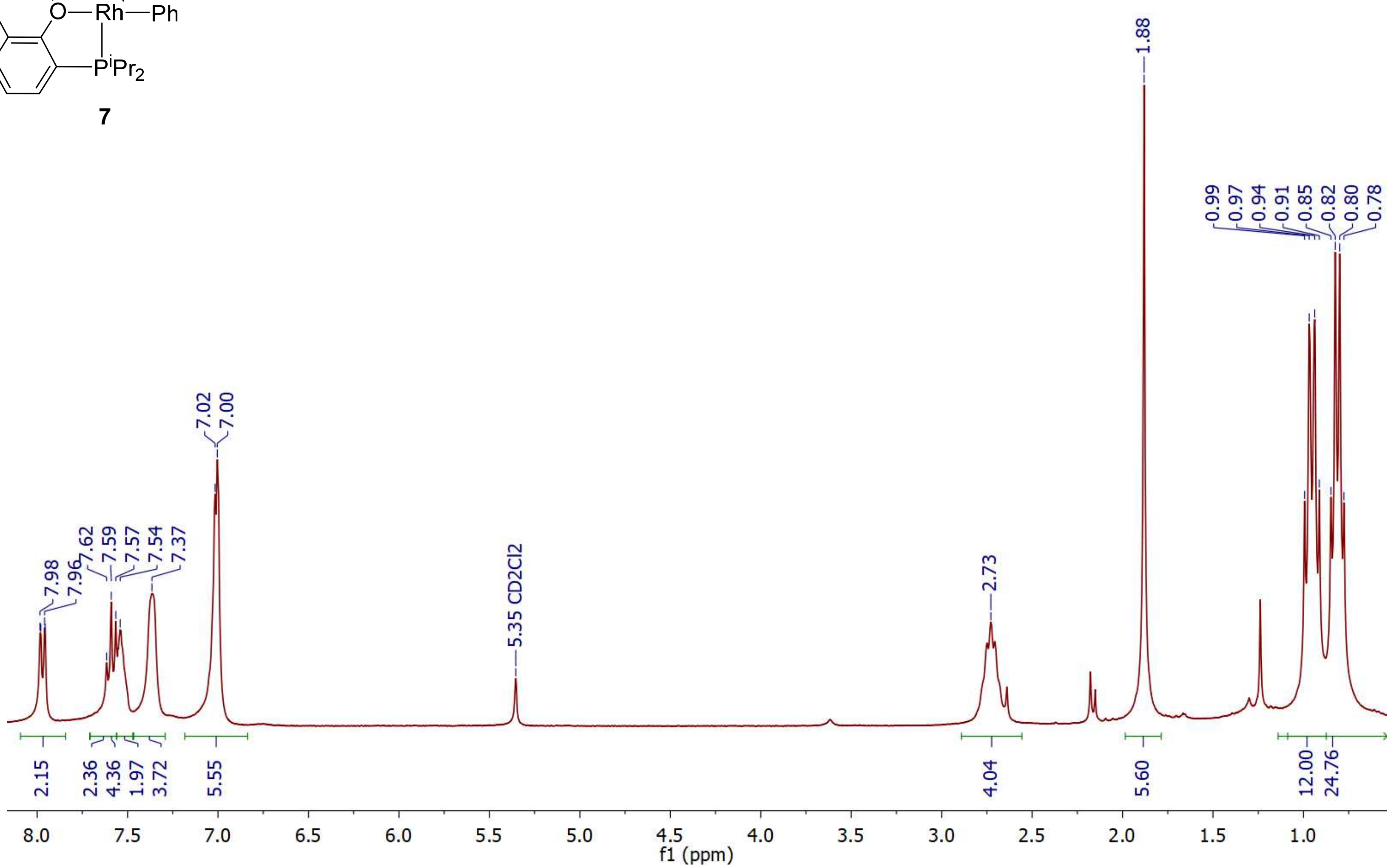

Figure S46. ^{1}H NMR spectrum (300.13 MHz, CD_2Cl_2, 298 K) of [RhPh$_2${κ^3-P,O,P-[xant(P^{i}Pr$_2$)$_2$]}]BF$_4$ (**7**).

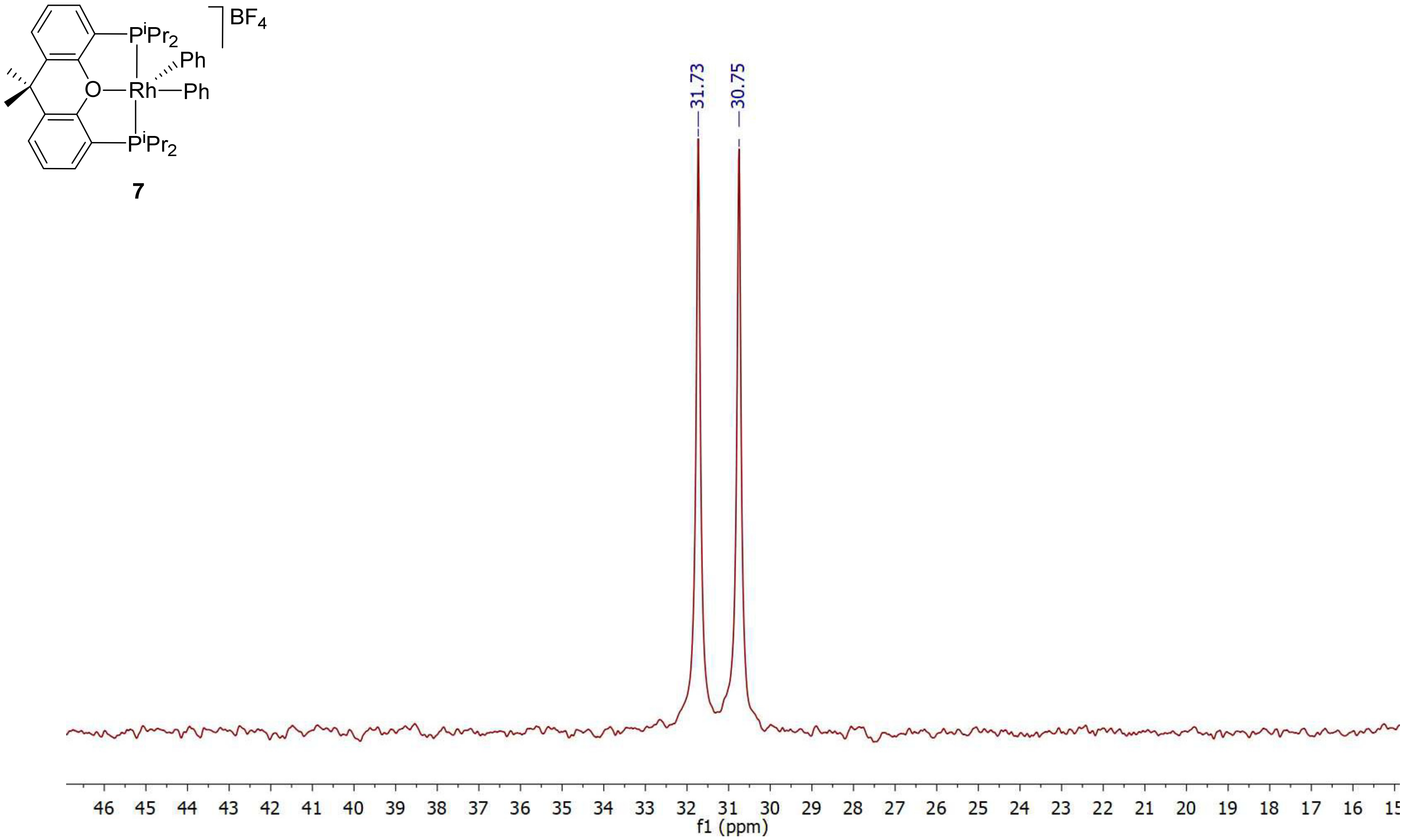

Figure S47. $^{31}P\{^1H\}$ NMR spectrum (121.50 MHz, CD_2Cl_2, 298 K) of $[RhPh_2\{\kappa^3$-P,O,P-[xant($P^iPr_2)_2$]}]BF_4 (**7**).

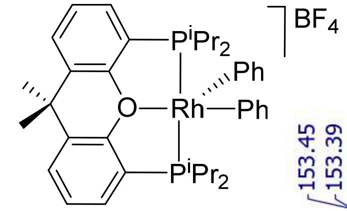

7

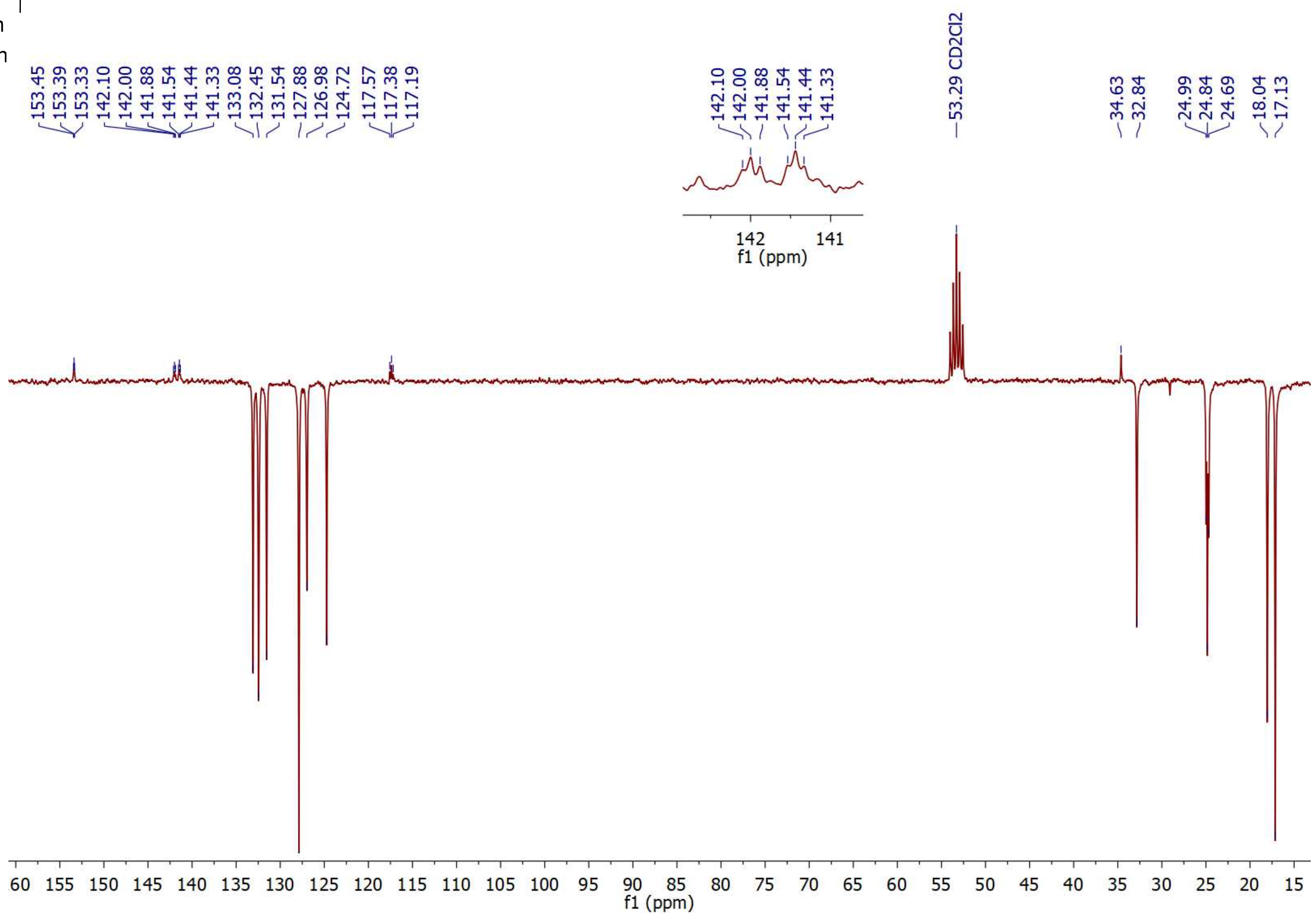

Figure S48. $^{13}C\{^1H\}$-apt NMR spectrum (75.48 MHz, CD_2Cl_2, 298 K) of $[RhPh_2\{\kappa^3\text{-P,O,P-}[xant(P^iPr_2)_2]\}]BF_4$ (**7**).

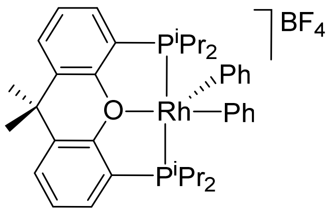

7

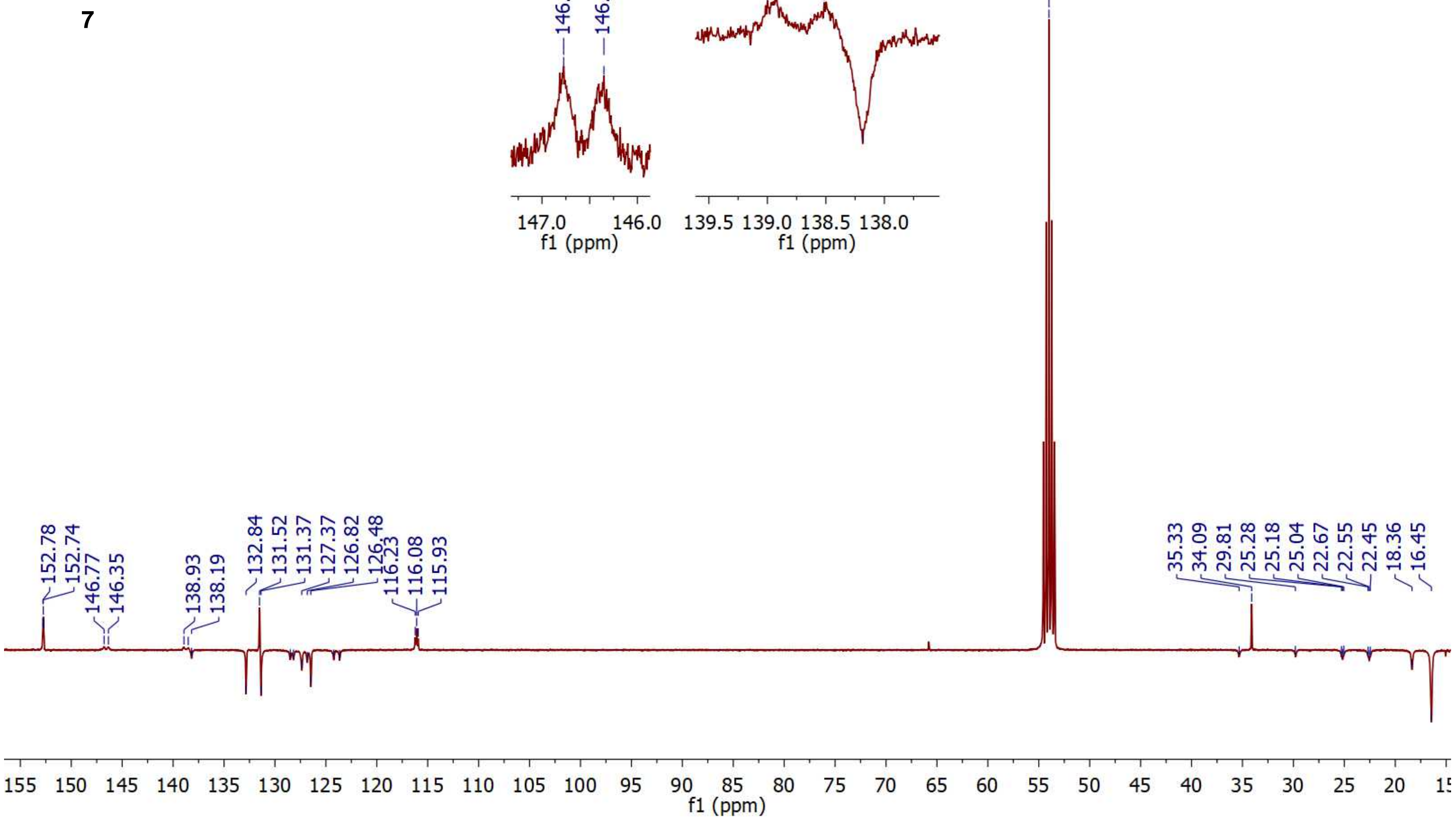

Figure S49. $^{13}C\{^{1}H\}$-apt NMR spectrum (100.62, CD_2Cl_2, 183 K) of $[RhPh_2\{\kappa^3$-P,O,P-[xant($P^iPr_2)_2]\}]BF_4$ (**7**).

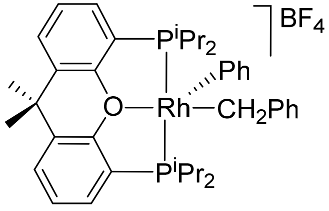

8

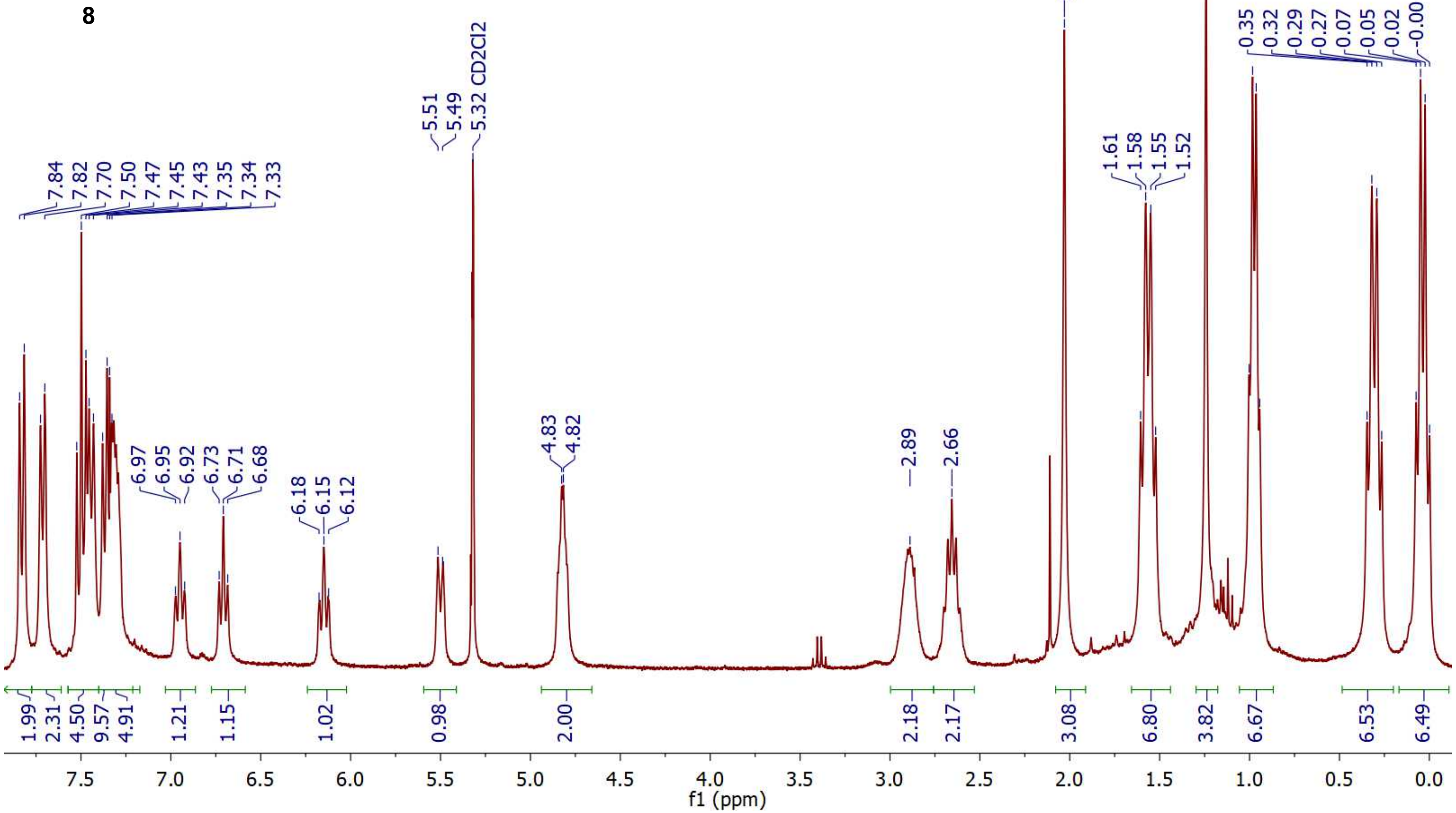

Figure S50. ^{1}H NMR spectrum (300.13 MHz, CD_2Cl_2, 233 K) of [RhPh(CH_2Ph){κ^3-P,O,P-[xant(P^iPr_2)$_2$]}]BF_4 (**8**).

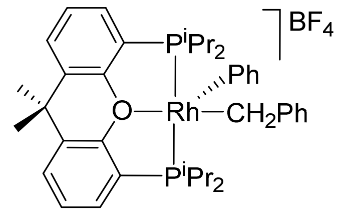

8

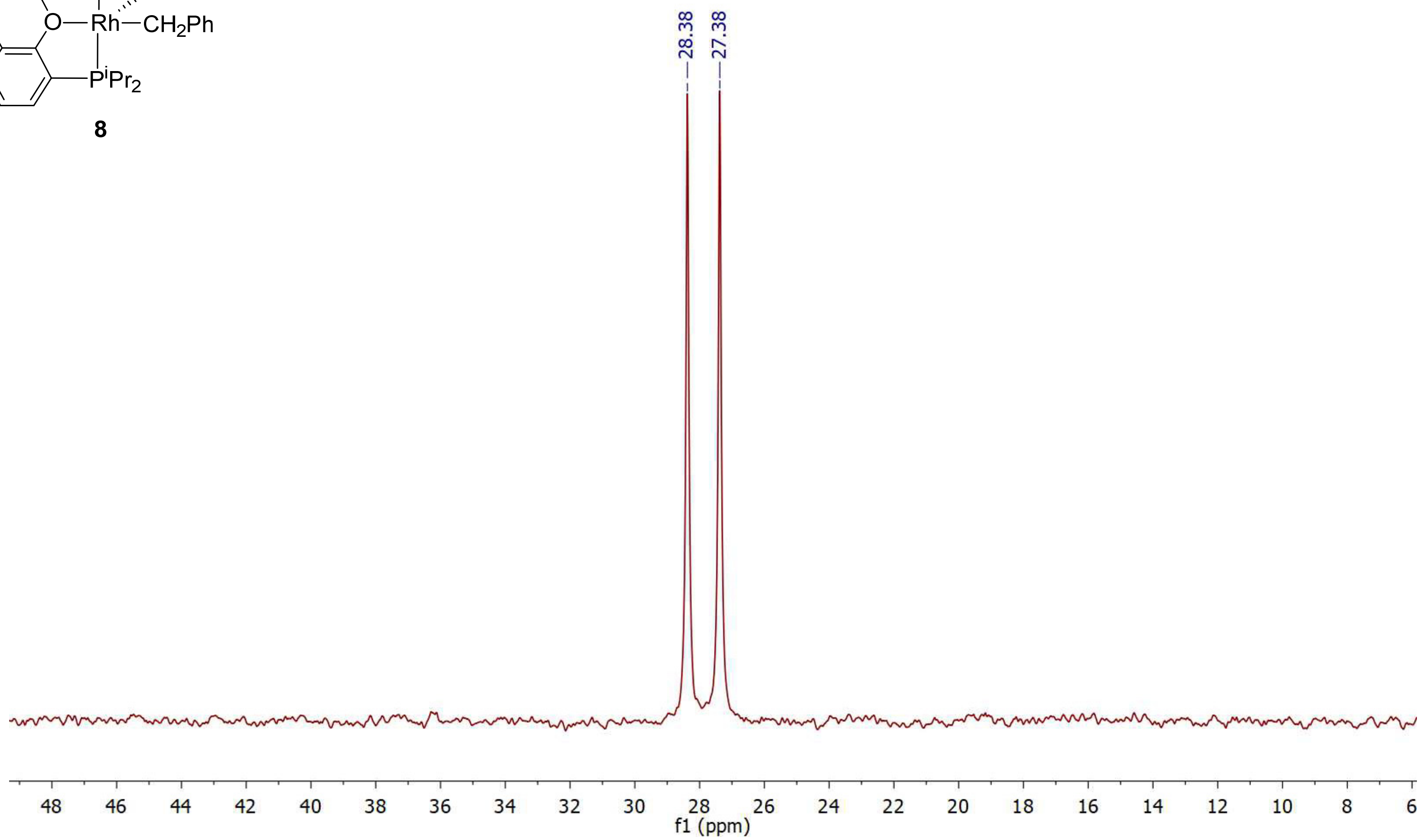

Figure S51. $^{31}P\{^1H\}$ NMR spectrum (121.50 MHz, CD_2Cl_2, 298 K) of $[RhPh(CH_2Ph)\{\kappa^3\text{-}P,O,P\text{-}[xant(P^iPr_2)_2]\}]BF_4$ (**8**).

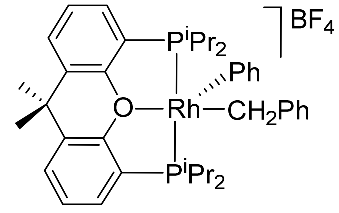

8

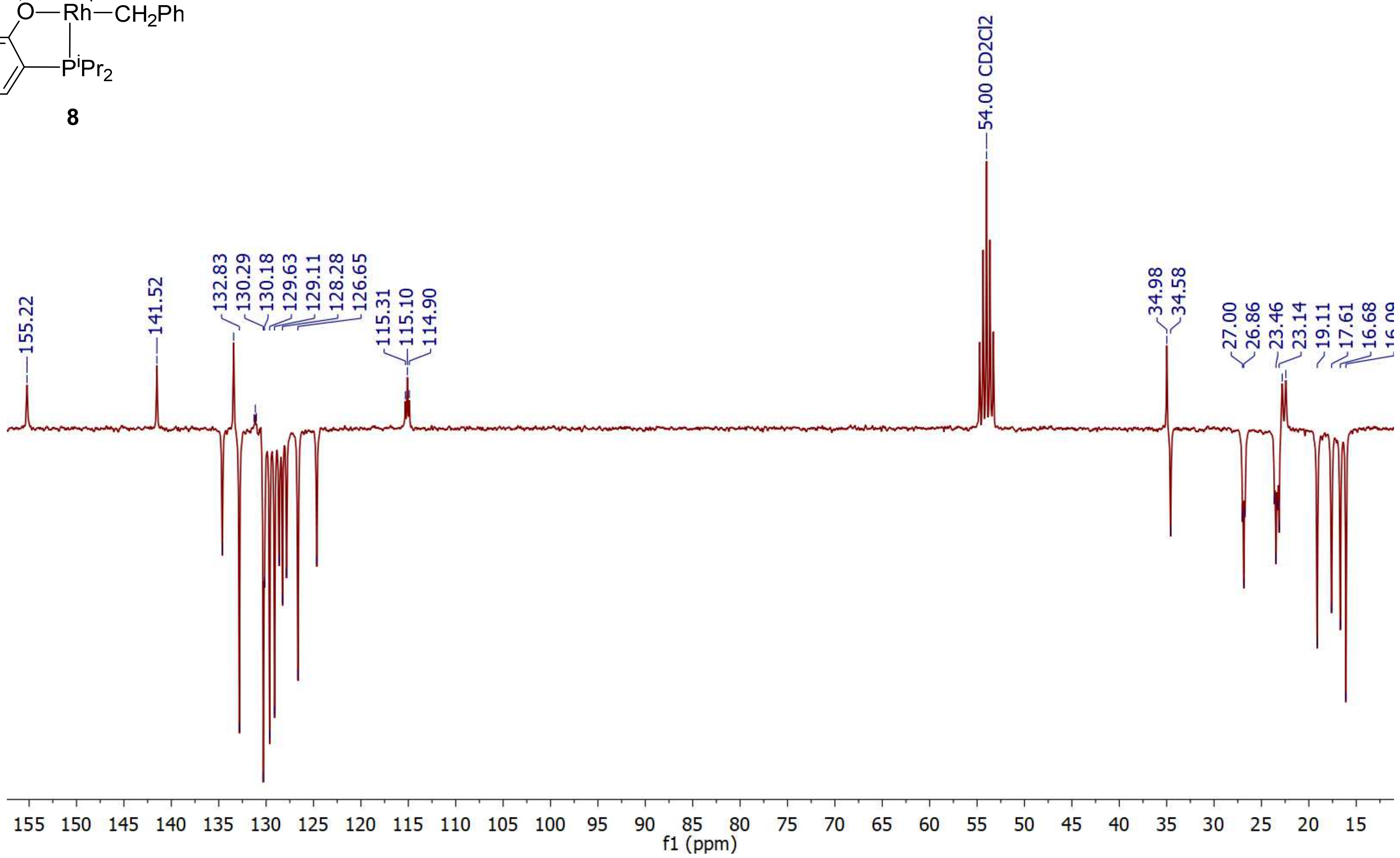

Figure S52. $^{13}C\{^{1}H\}$-apt NMR spectrum (75.48 MHz, CD_2Cl_2, 233 K) of $[RhPh(CH_2Ph)\{\kappa^3\text{-P,O,P-}[xant(P^iPr_2)_2]\}]BF_4$ (**8**).

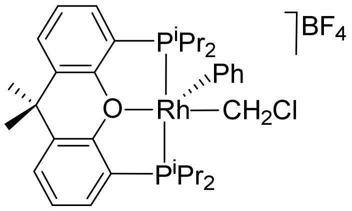

9

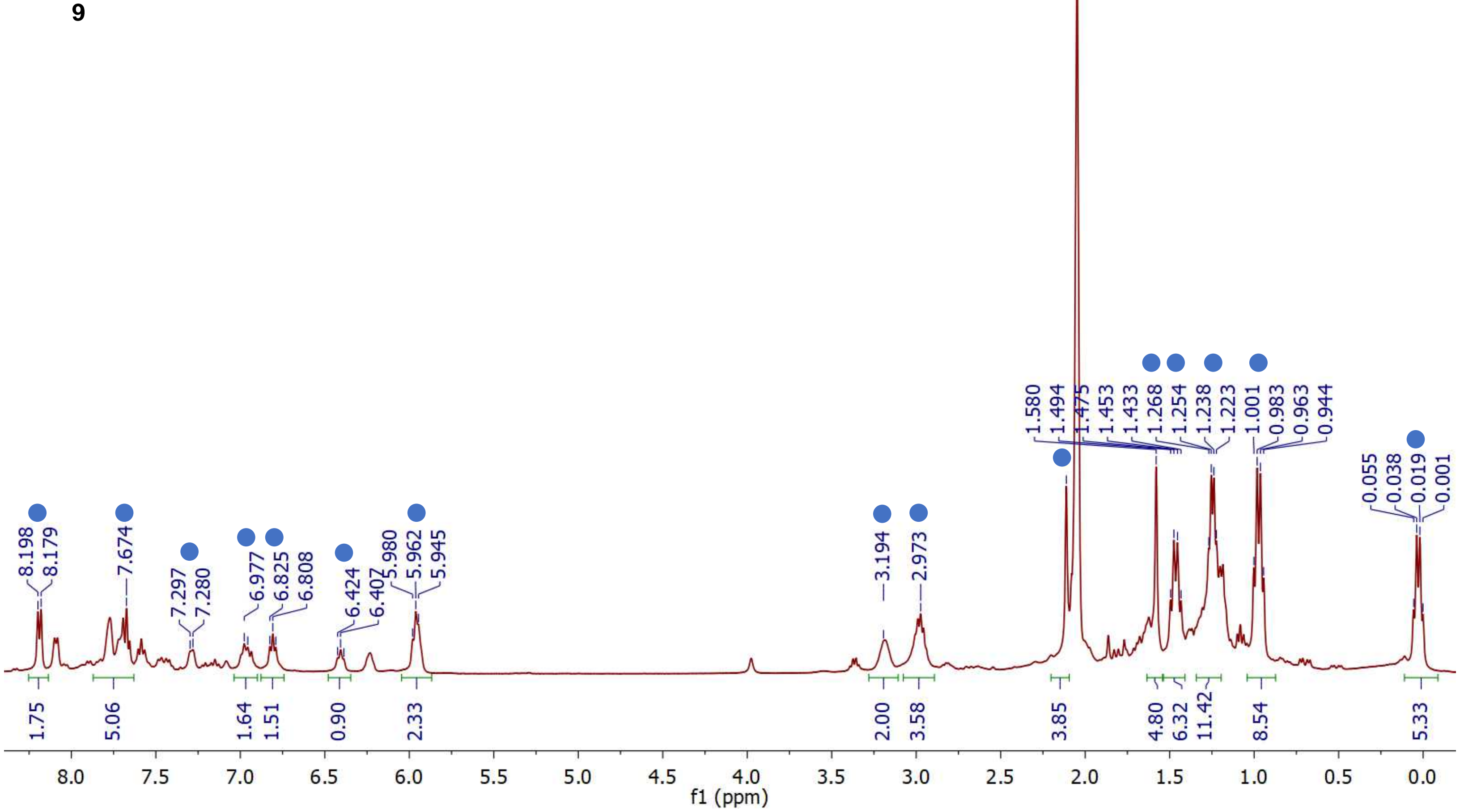

Figure S53. ^{1}H NMR spectrum (400.13 MHz, acetone-d_6, 243 K) of the solid resulting from the abstraction of chloride of RhPh(CH$_2$Cl)Cl{κ^3-P,O,P-[xant(P^{i}Pr$_2$)$_2$]} (**5a**,**b**). Resonances assigned to [RhPh(CH$_2$Cl){κ^3-P,O,P-[xant(P^{i}Pr$_2$)$_2$]}]BF$_4$ (**9**) are marked with a blue spot.

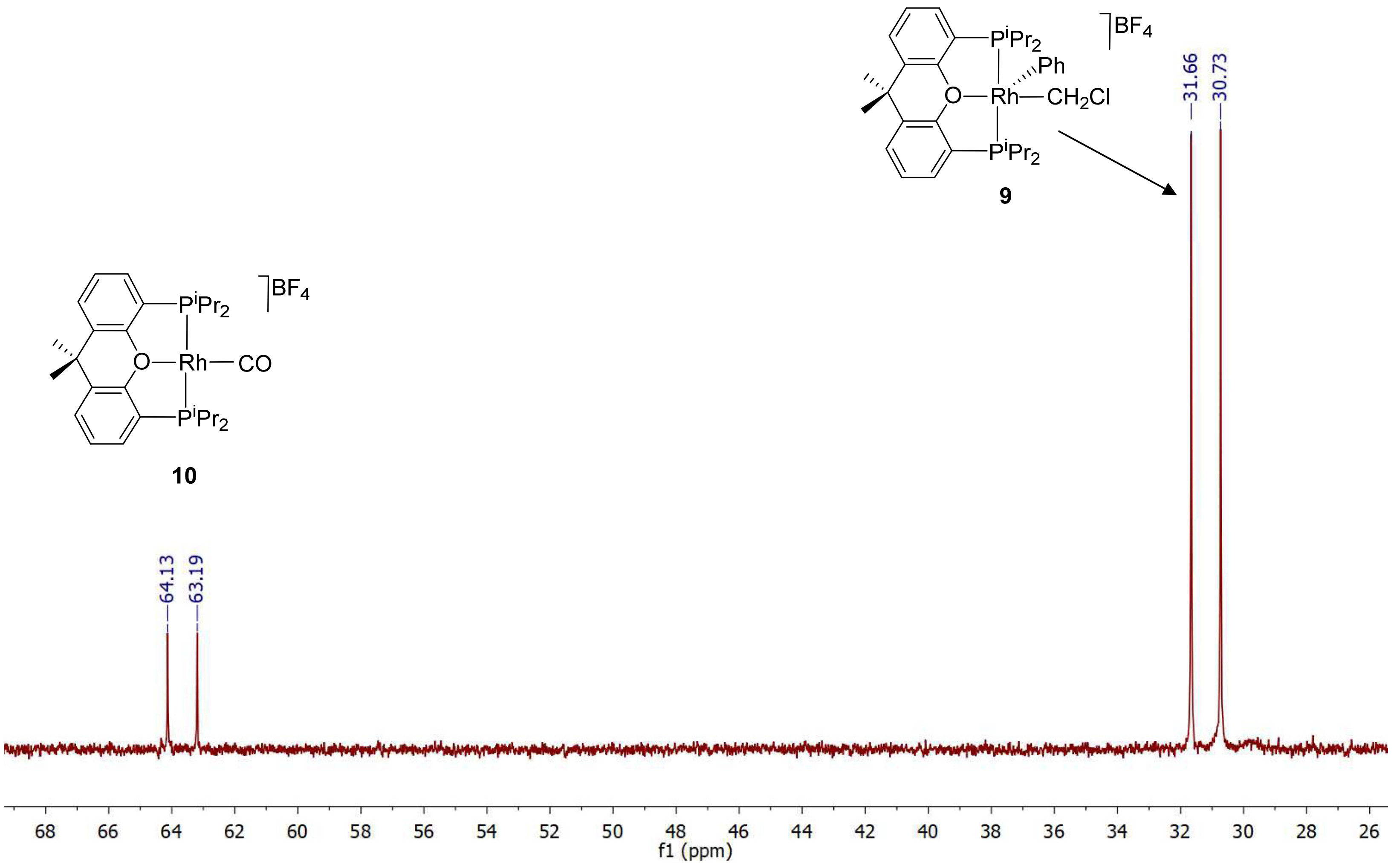

Figure S54. $^{31}P\{^{1}H\}$ NMR spectrum (161.98 MHz, acetone-d_6, 243 K) of the solid resulting from the abstraction of chloride of RhPh(CH_2Cl)Cl{κ^3-P,O,P-[xant(P^iPr_2)$_2$]} (**5a**,**b**).

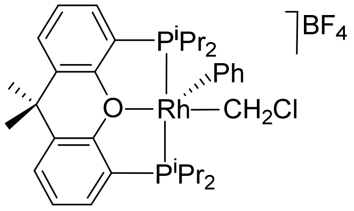

9

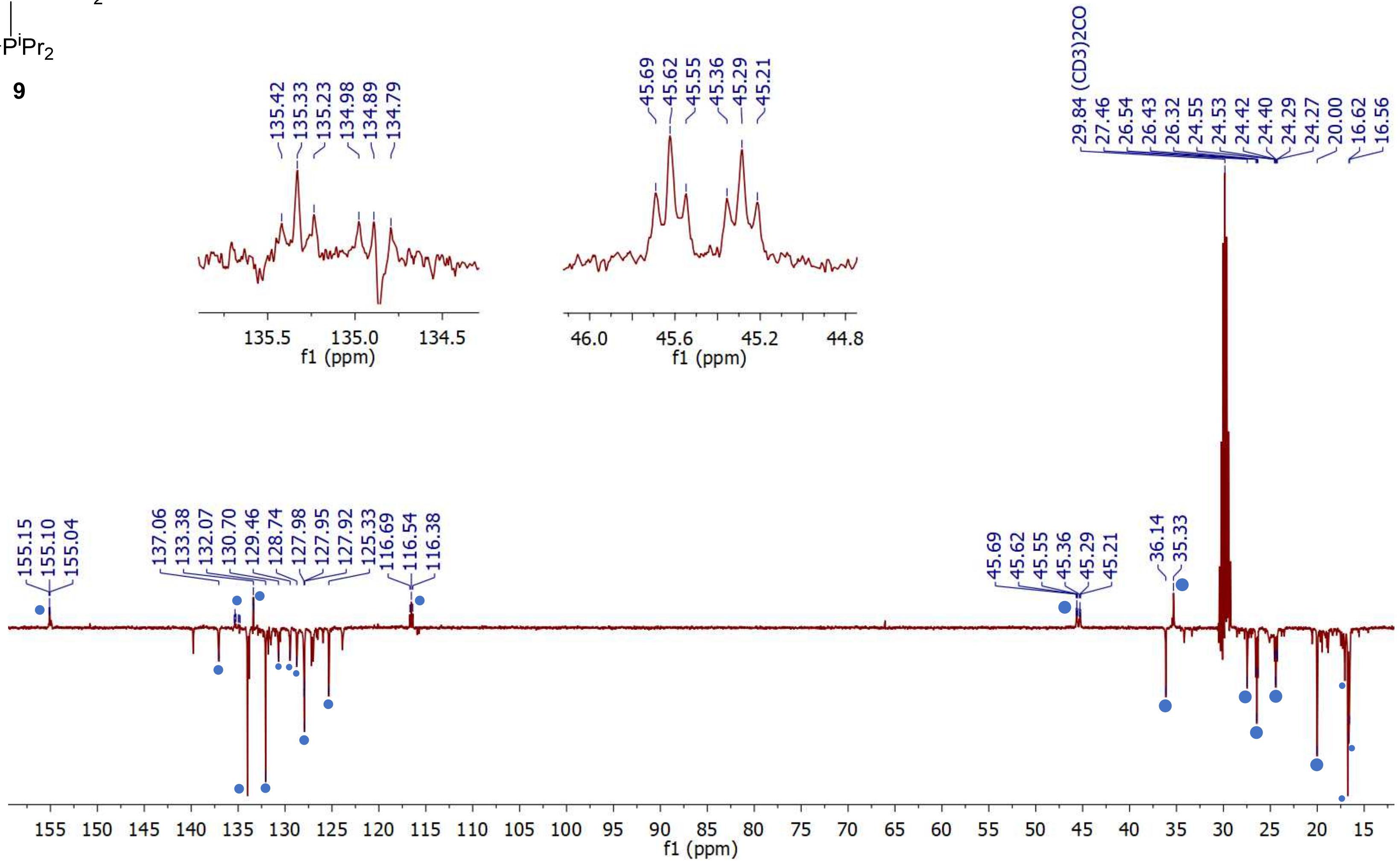

Figure S55. $^{13}C\{^1H\}$-apt NMR spectrum (100.62 MHz, acetone-d_6, 253 K) of the solid resulting from the abstraction of chloride of RhPh(CH_2Cl)Cl{κ^3-P,O,P-[xant(P^iPr_2)$_2$]} (**5a**,**b**). Resonances assigned to [RhPh(CH_2Cl){κ^3-P,O,P-[xant(P^iPr_2)$_2$]}]BF_4 (**9**) are marked with a blue spot.

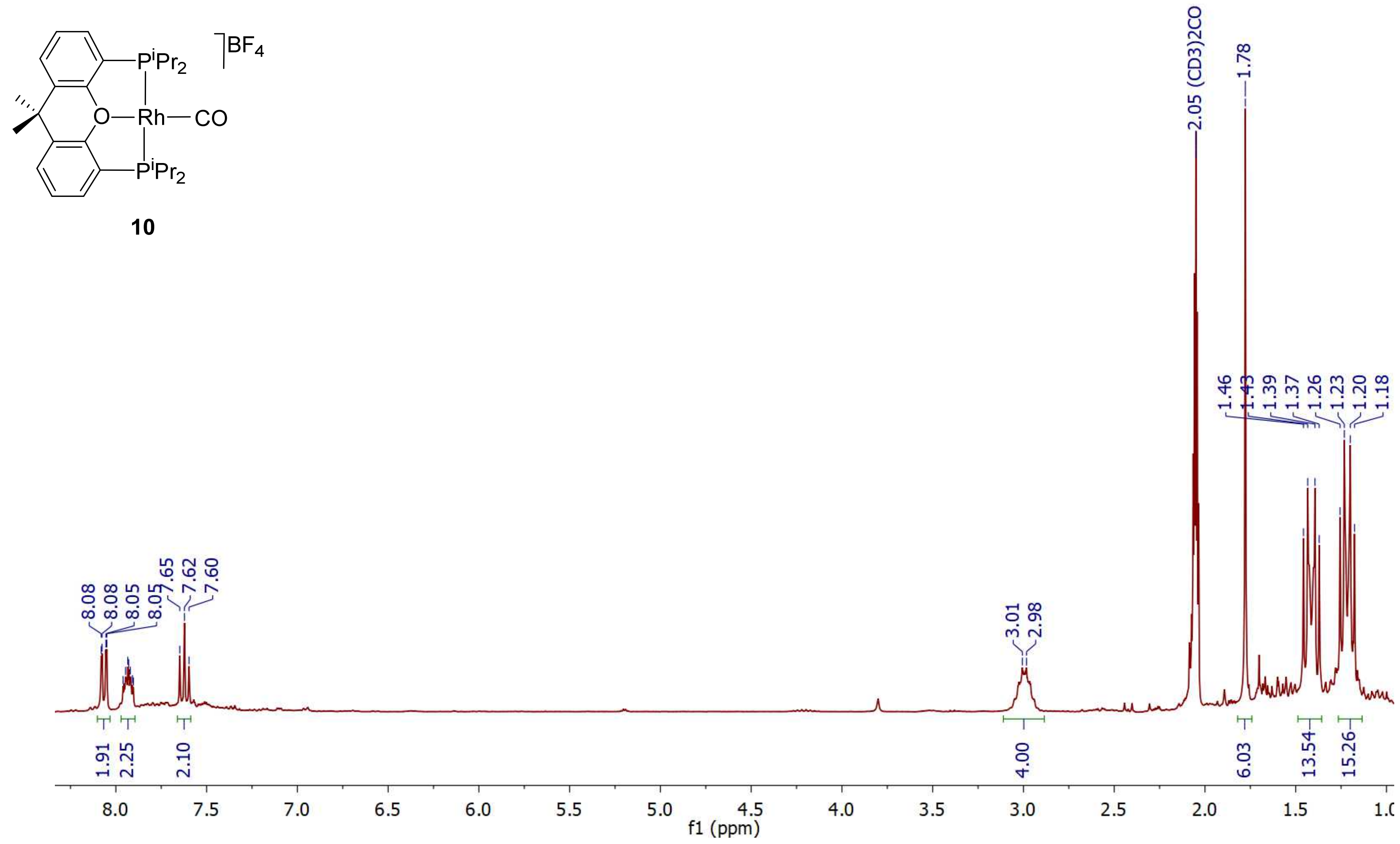

Figure S56. ^{1}H NMR spectrum (300.13 MHz, acetone-d_6, 273 K) of [Rh(CO){κ^3-P,O,P-[xant(P^{i}Pr$_2$)$_2$]}]BF$_4$ (**10**).

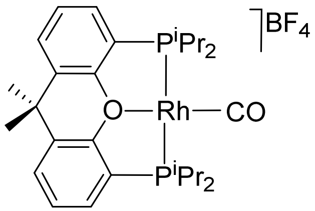

10

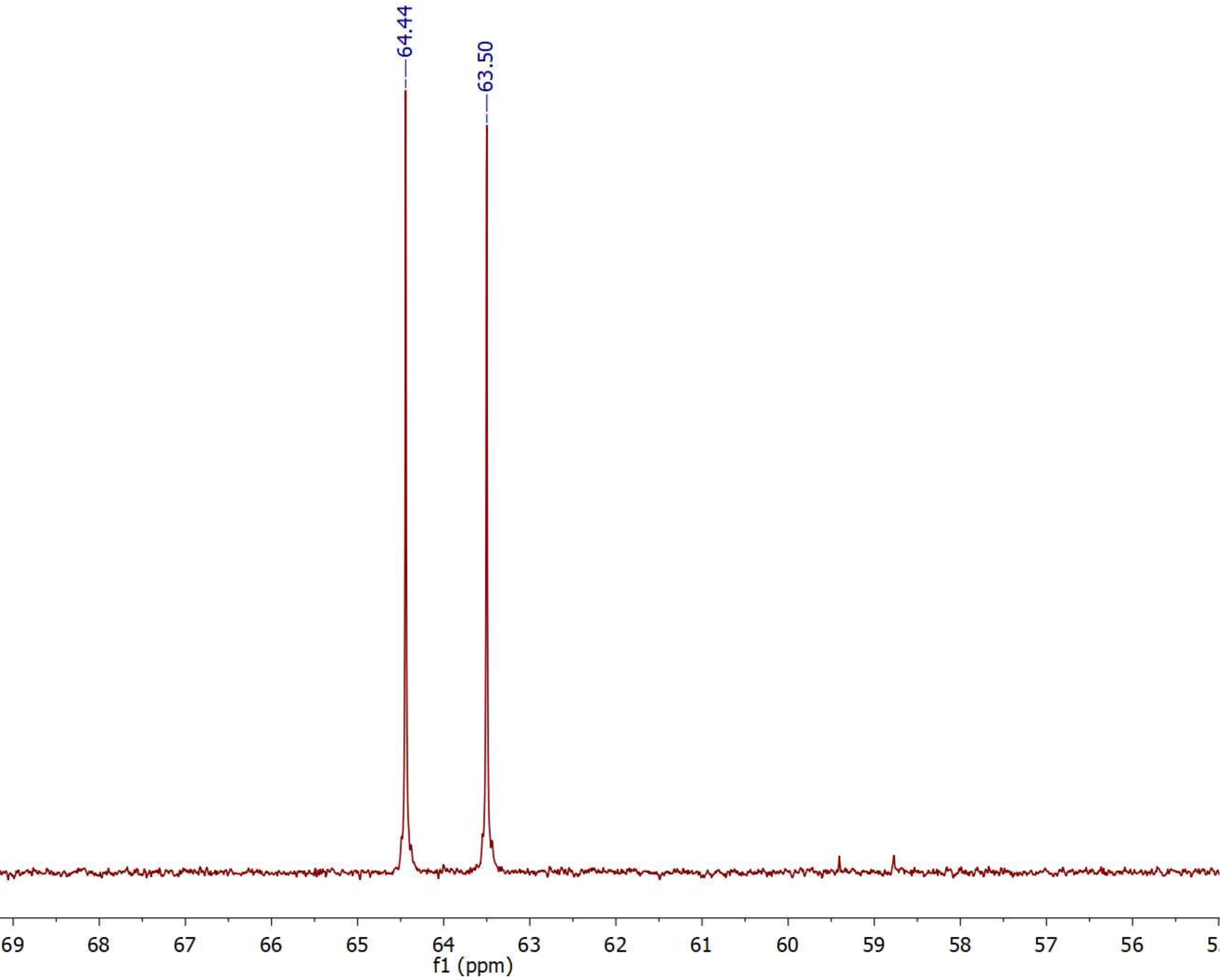

Figure S57. $^{31}P\{^1H\}$ NMR spectrum (121.50 MHz, acetone-d_6, 273 K) of $[Rh(CO)\{\kappa^3\text{-}P,O,P\text{-}[xant(P^iPr_2)_2]\}]BF_4$ (**10**).

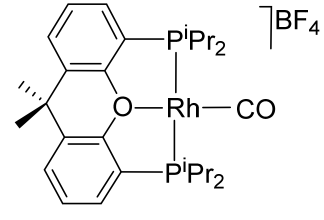

10

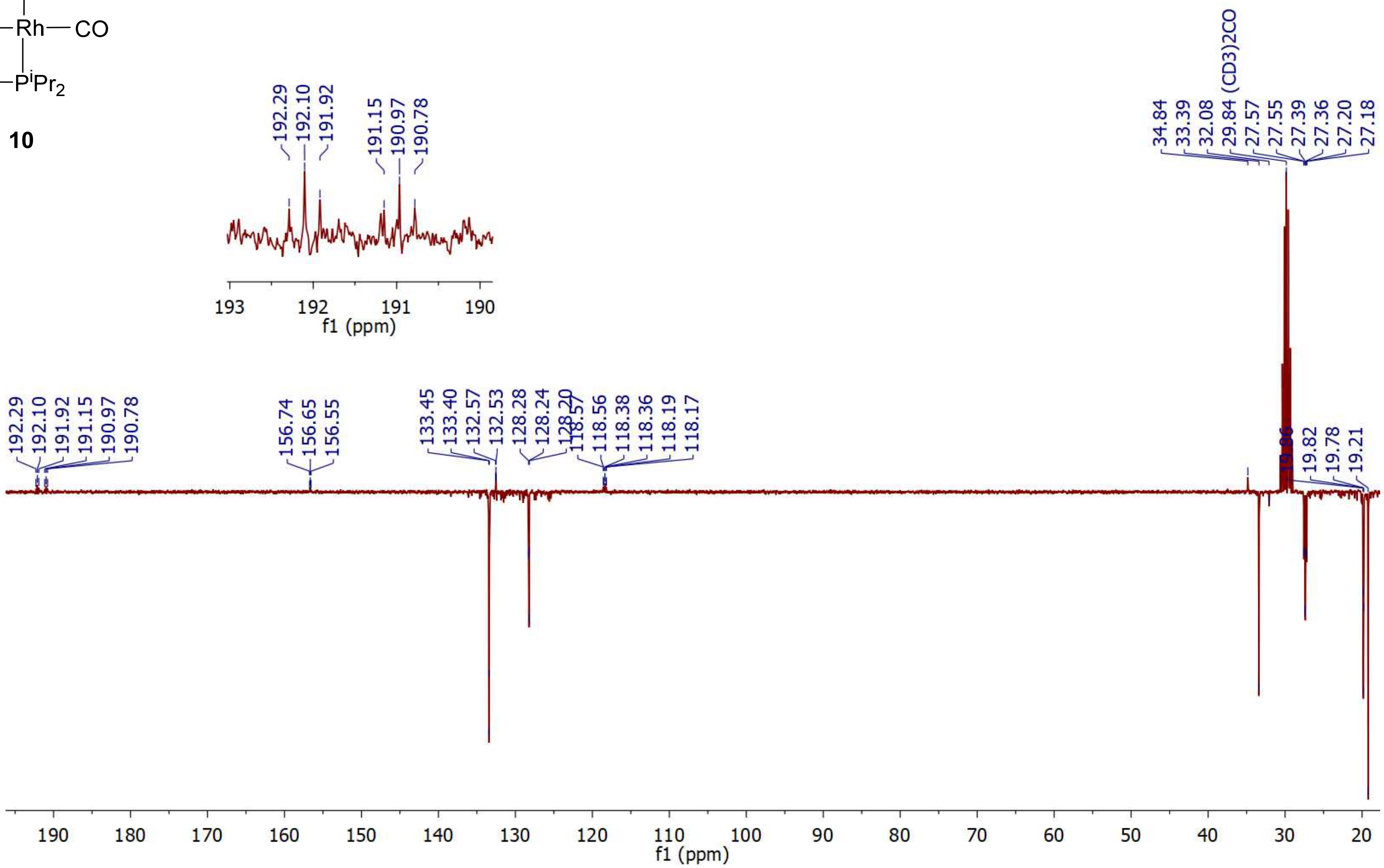

Figure S58. $^{13}C\{^{1}H\}$-apt NMR spectrum (75.48 MHz, acetone-d_6, 273 K) of $[Rh(CO)\{\kappa^3\text{-P,O,P-}[xant(P^iPr_2)_2]\}]BF_4$ (**10**).

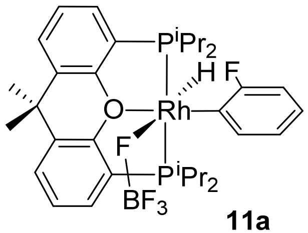

11a

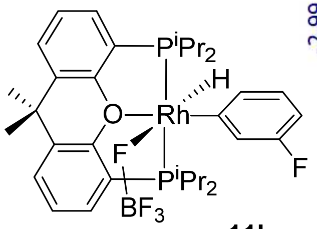

11b

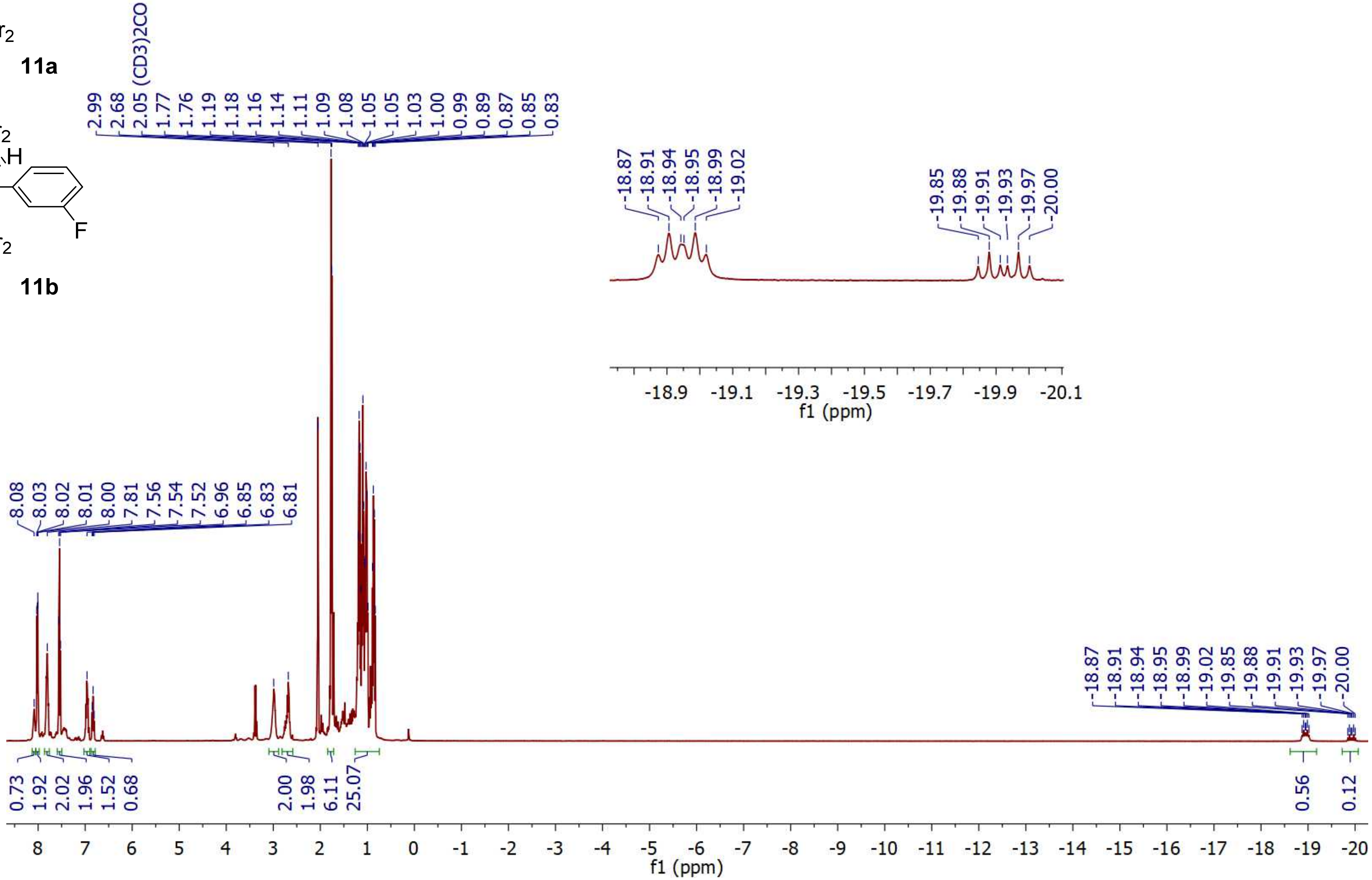

Figure S59. ^{1}H NMR spectrum (400.13 MHz, acetone-d_6, 273 K) of the isomeric mixture 70:30 of RhH(o-C_6H_4F)(κ^1-FBF_3){κ^3-P,O,P-[xant(P^iPr_2)$_2$]} (**11a**) and RhH(m-C_6H_4F)(κ^1-FBF_3){κ^3-P,O,P-[xant(P^iPr_2)$_2$]} (**11b**).

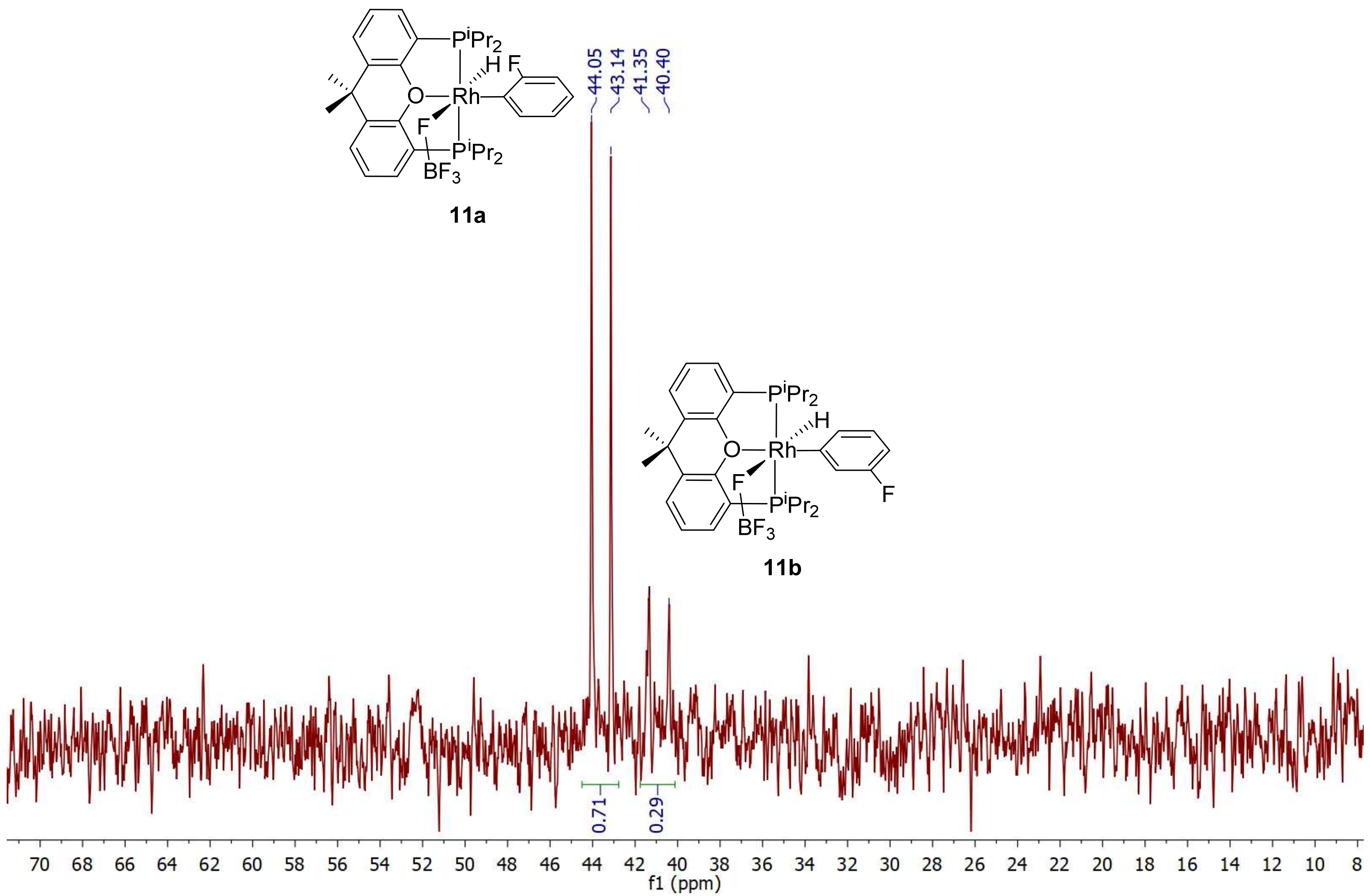

Figure S60. $^{31}P\{^1H\}$ NMR spectrum (121.4 MHz, acetone-d_6, 298 K) of the isomeric mixture 70:30 of RhH(*o*-C_6H_4F)(κ^1-FBF_3)\{κ^3-P,O,P-[xant(P^iPr_2)$_2$]\} (**11a**) and RhH(*m*-C_6H_4F)(κ^1-FBF_3)\{κ^3-P,O,P-[xant(P^iPr_2)$_2$]\} (**11b**).

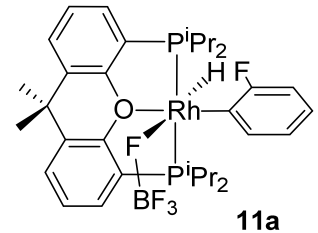

11a

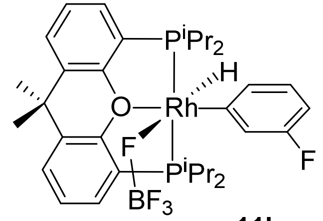

11b

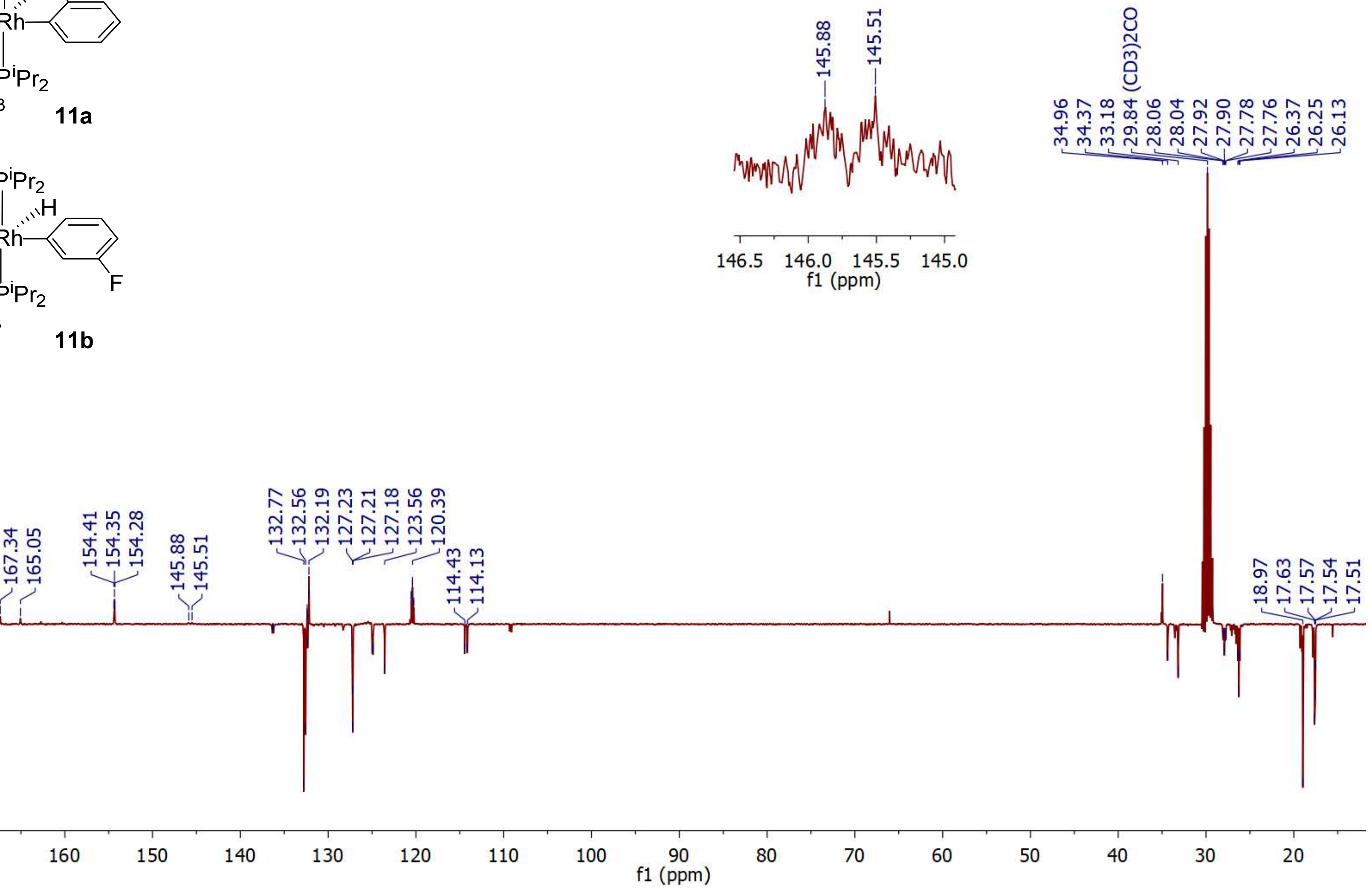

Figure S61. $^{13}C\{^1H\}$-apt NMR spectrum (100.63 MHz, acetone-d_6, 273 K) of the isomeric mixture 70:30 of RhH(o-C_6H_4F)(κ^1-FBF_3)\{κ^3-P,O,P-[xant(P^iPr_2)$_2$]\} (**11a**) and RhH(m-C_6H_4F)(κ^1-FBF_3)\{κ^3-P,O,P-[xant(P^iPr_2)$_2$]\} (**11b**).

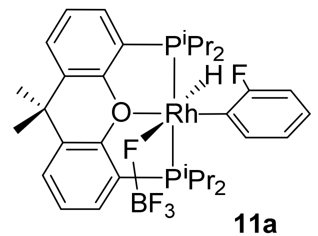

11a

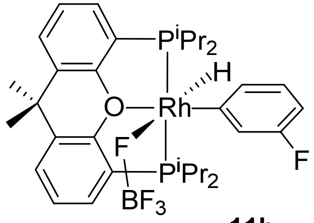

11b

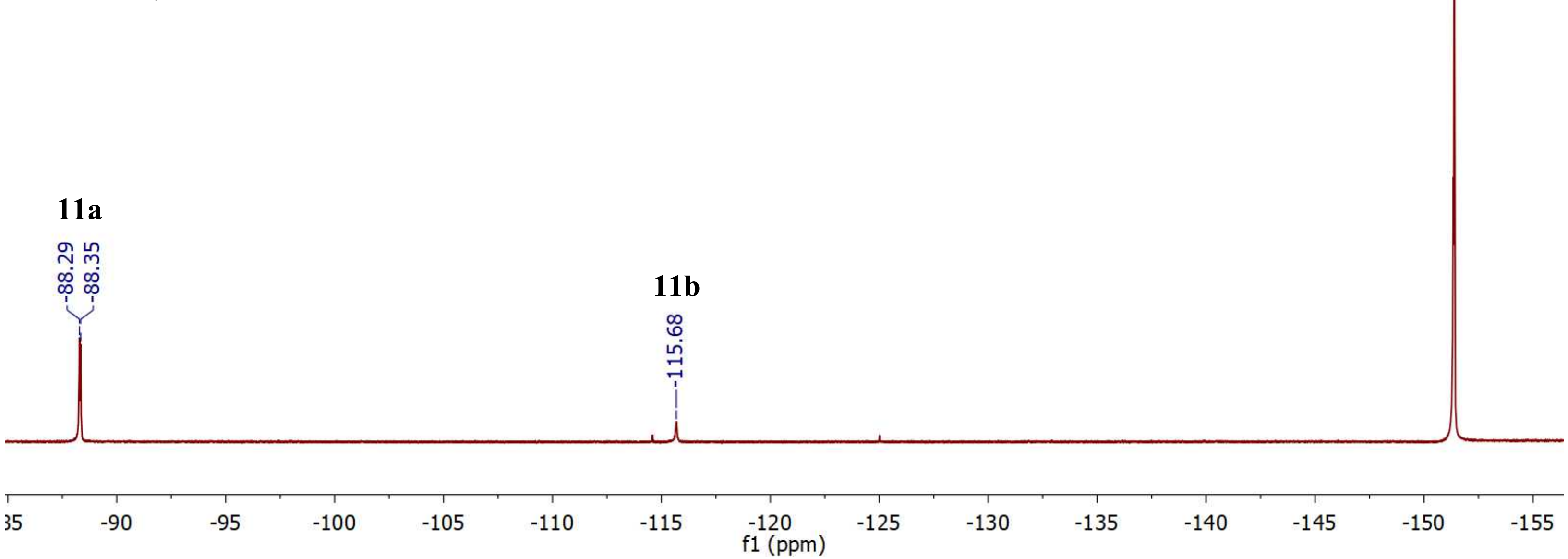

Figure S62. $^{19}F\{^1H\}$ NMR (376.46 MHz, acetone-d_6, 273 K) of the isomeric mixture 70:30 of RhH(o-C_6H_4F)(κ^1-FBF_3){κ^3-P,O,P-[xant(P^iPr_2)$_2$]} (**11a**) and RhH(m-C_6H_4F)(κ^1-FBF_3){κ^3-P,O,P-[xant(P^iPr_2)$_2$]} (**11b**).

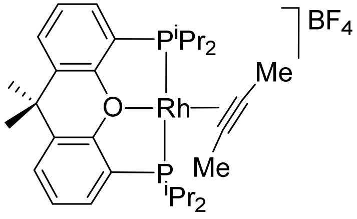

13

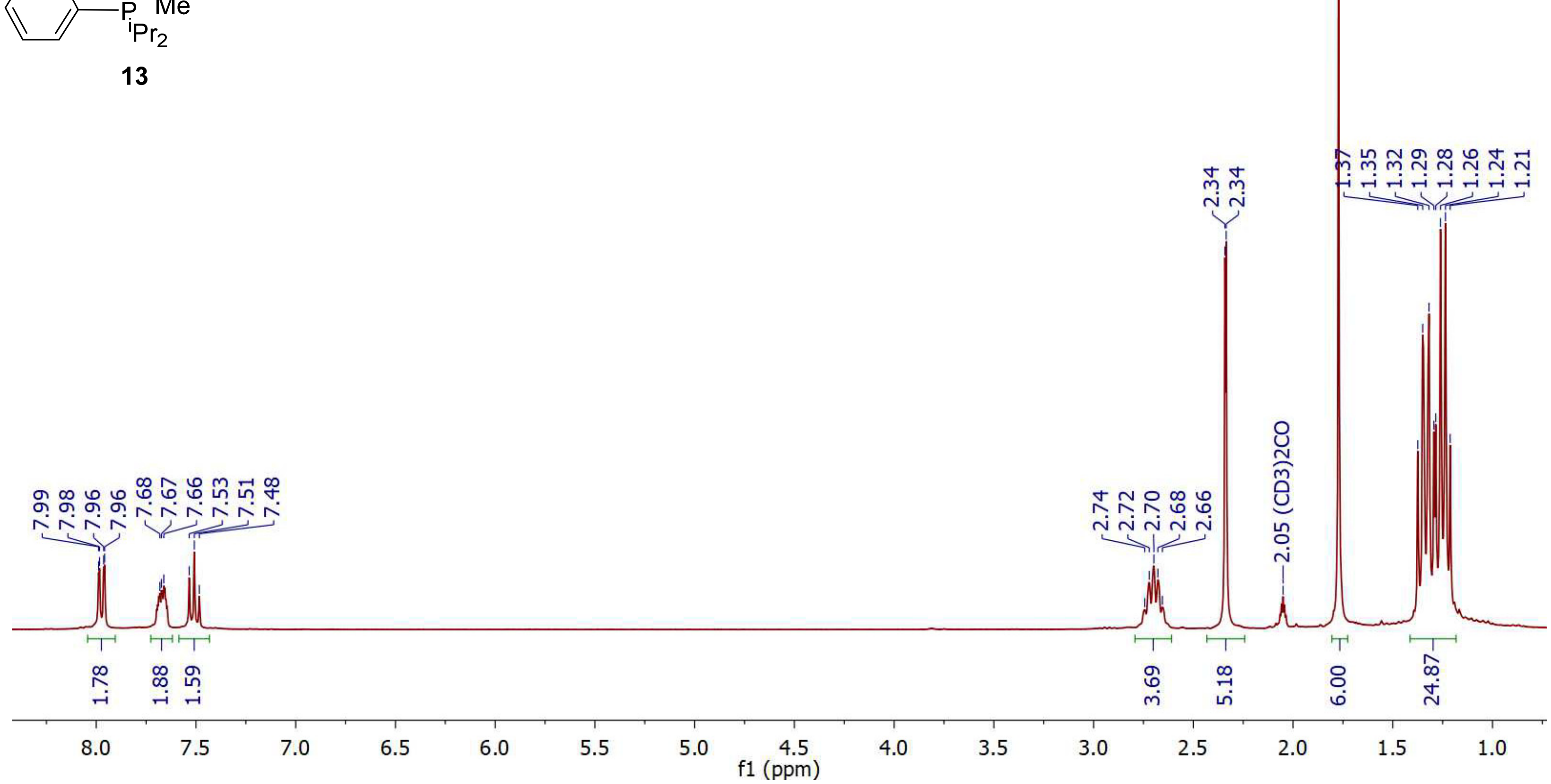

Figure S63. ^{1}H NMR spectrum (300.13 MHz, acetone-d_6, 298 K) of [Rh(η^2-MeC≡CMe){κ^3-P,O,P-[xant(P^{i}Pr$_2$)$_2$]}]BF$_4$ (**13**).

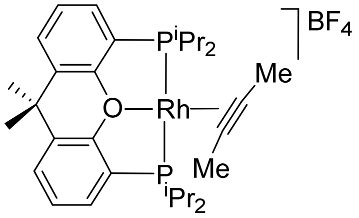

13

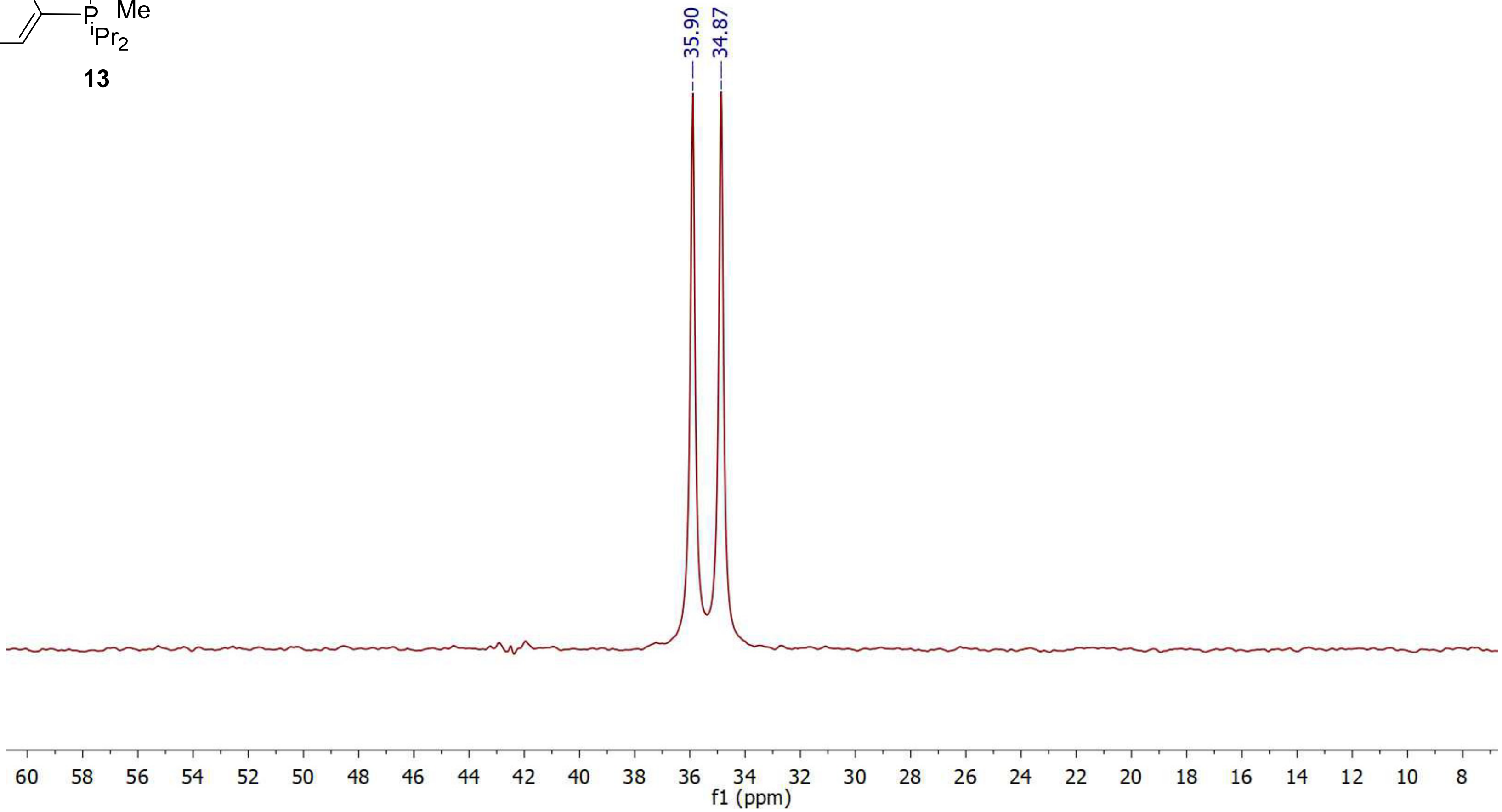

Figure S64. $^{31}P\{^{1}H\}$ NMR spectrum (121.49 MHz, acetone-d_6, 298 K) of $[Rh(\eta^2\text{-}MeC{\equiv}CMe)\{\kappa^3\text{-}P,O,P\text{-}[xant(P^iPr_2)_2]\}]BF_4$ (**13**).

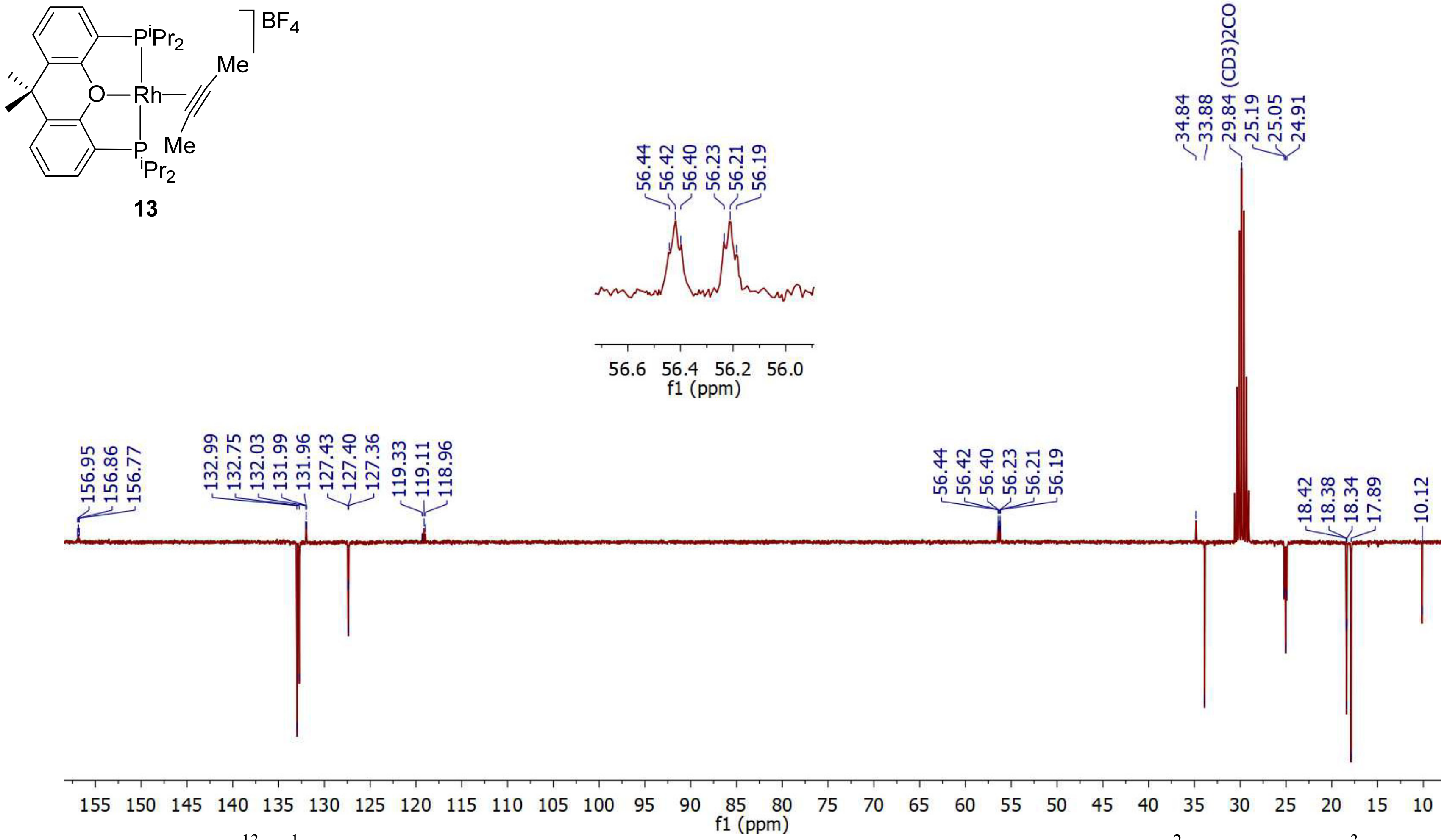

Figure S65. $^{13}C\{^{1}H\}$-apt NMR spectrum (75.48 MHz, acetone-d_6, 273 K) of [Rh(η^2-MeC≡CMe){κ^3-P,O,P-[xant(P^{i}Pr$_2$)$_2$]}]BF_4 (**13**).

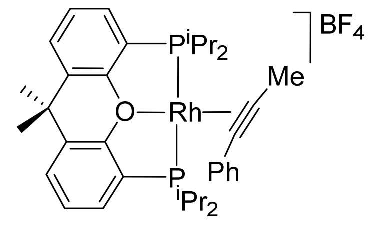

14

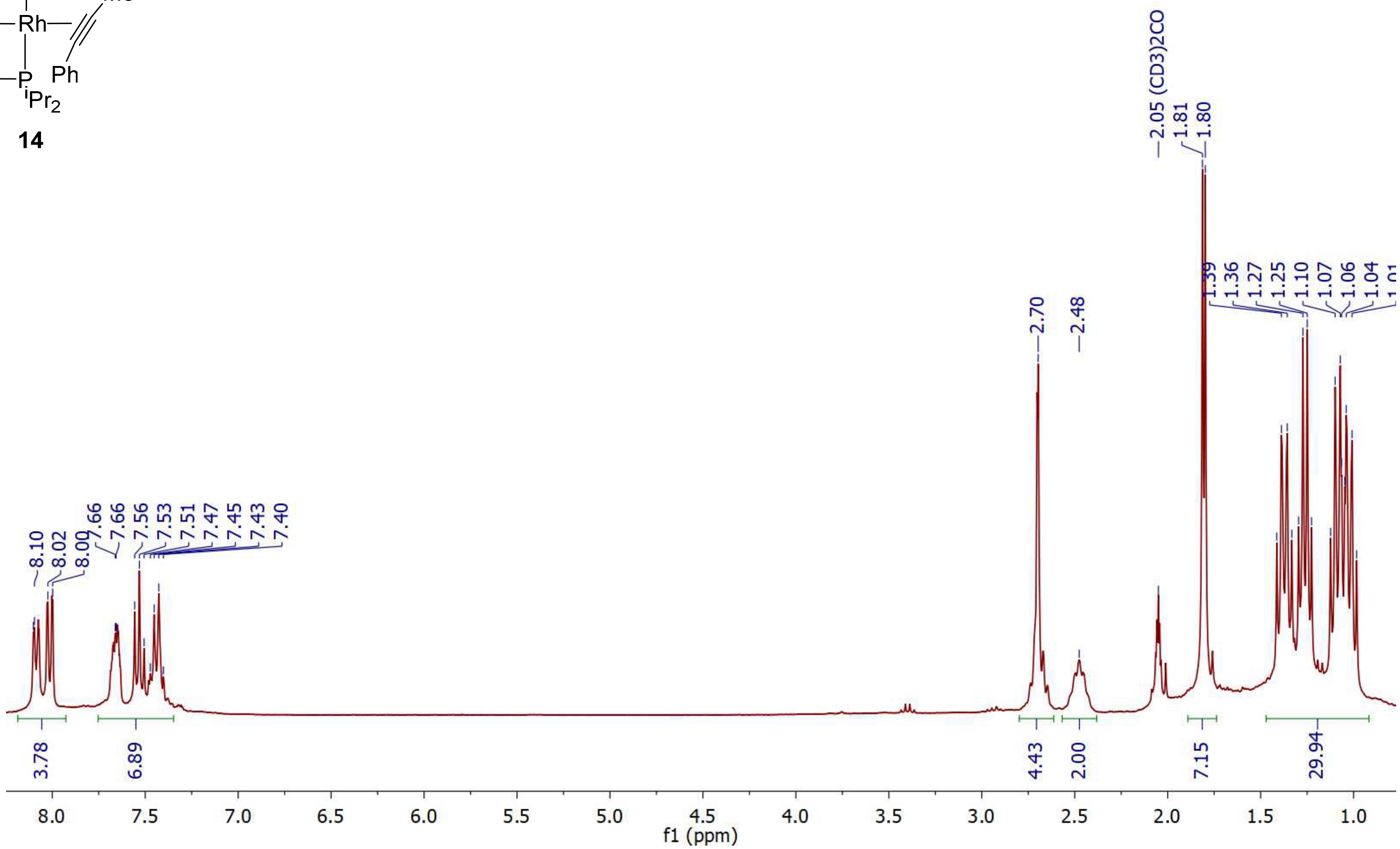

Figure S66. ^{1}H NMR spectrum (300.13 MHz, acetone-d_6, 298 K) of [Rh(η^2-PhC≡CMe){κ^3-P,O,P-[xant(P^{i}Pr$_2$)$_2$]}]BF_4 (**14**).

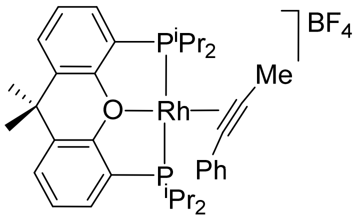

14

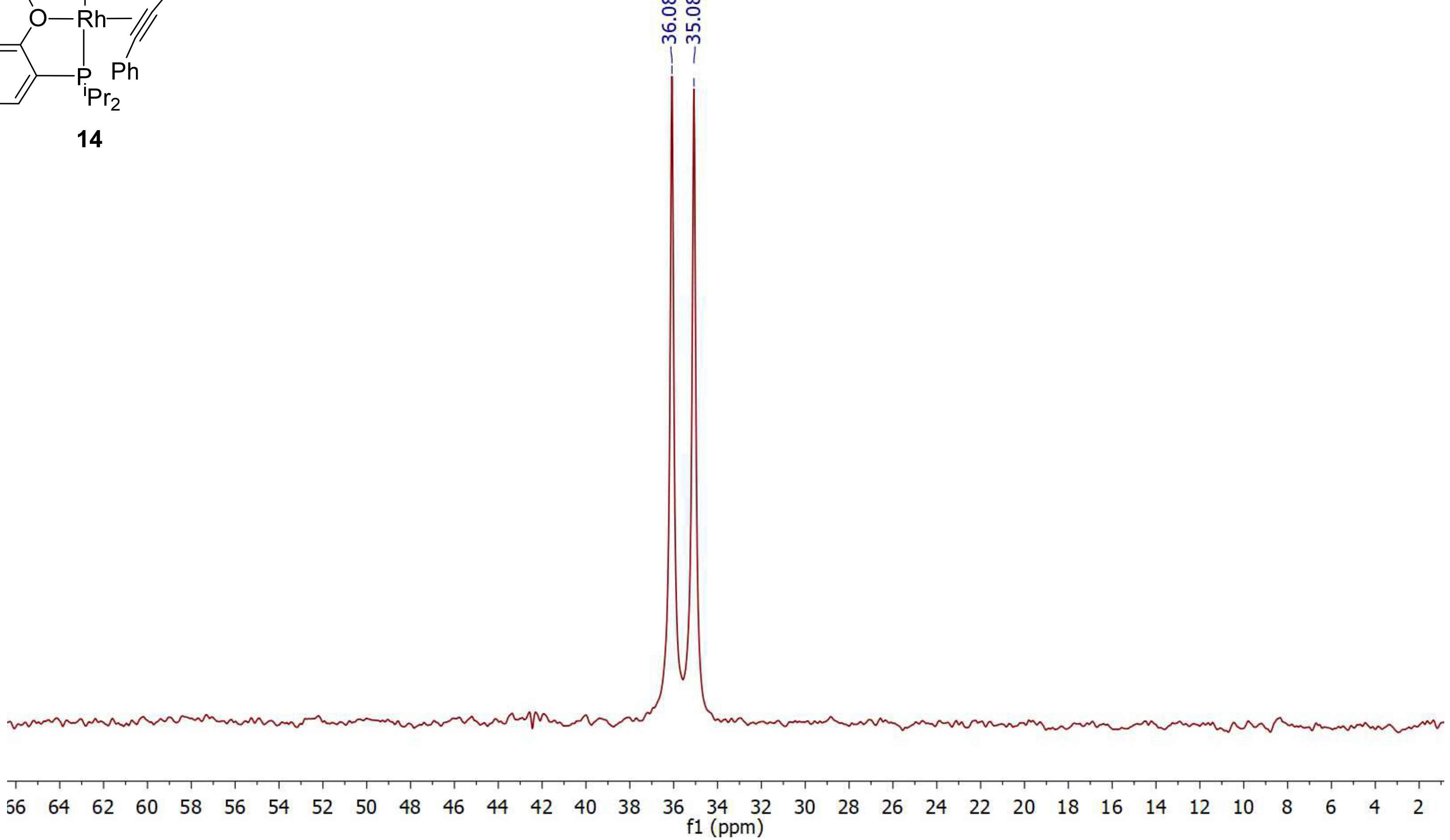

Figure S67. $^{31}P\{^1H\}$ NMR spectrum (121.49 MHz, acetone-d_6, 298 K) of [Rh(η^2-PhC≡CMe){κ^3-P,O,P-[xant(P^{i}Pr$_2$)$_2$]}]BF_4 (**14**).

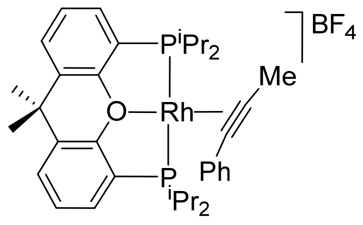

14

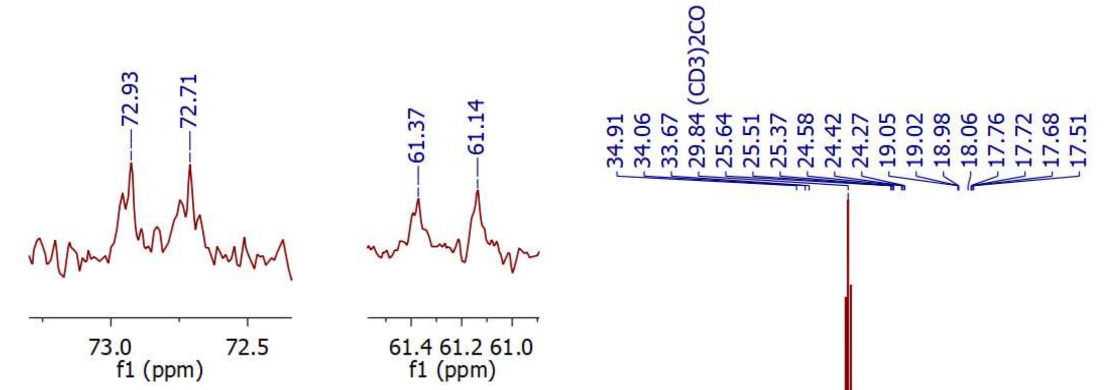
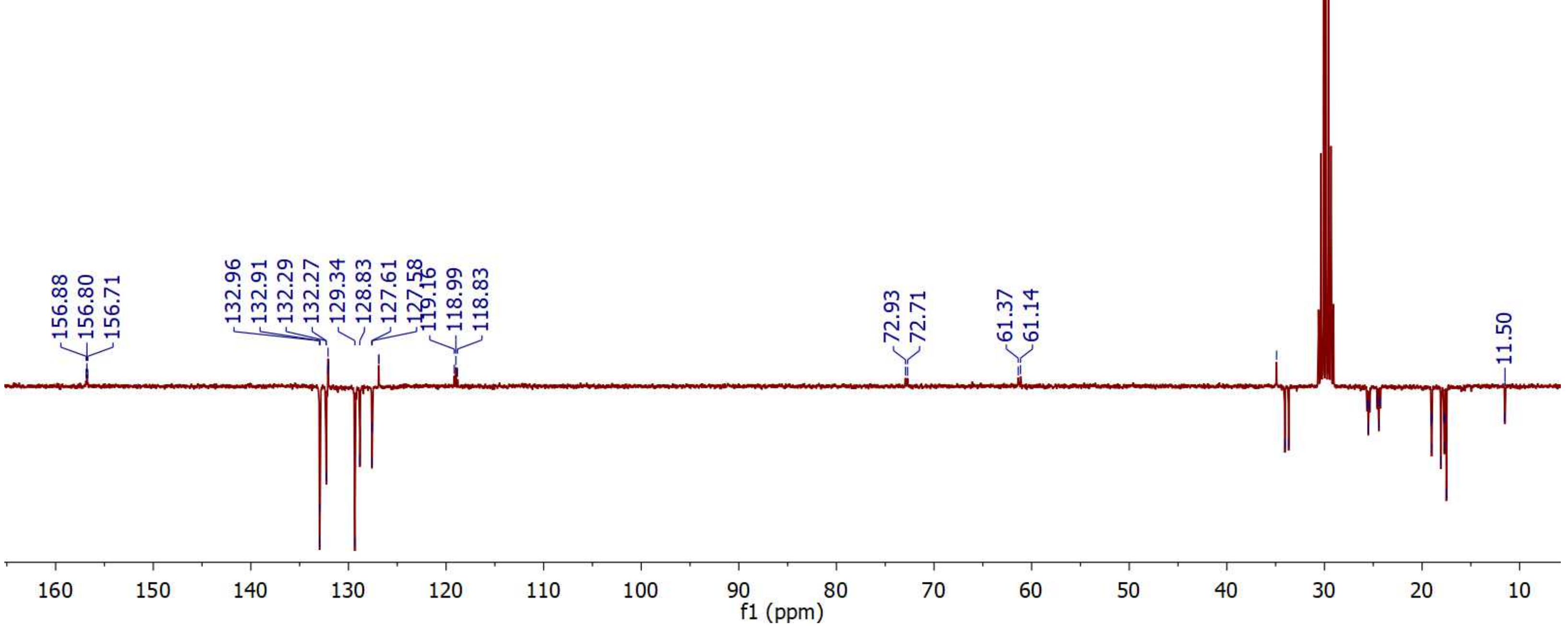

Figure S68. $^{13}C\{^1H\}$-apt NMR spectrum (75.48 MHz, acetone-d_6, 298 K) of [Rh(η^2-PhC≡CMe){κ^3-P,O,P-[xant(P^iPr_2)$_2$]}]BF_4 (**14**).
